# Small Molecule MIF Modulation Enhances Ferroptosis by Impairing DNA Repair Mechanisms

**DOI:** 10.1002/advs.202403963

**Published:** 2024-06-25

**Authors:** Deng Chen, Chunlong Zhao, Jianqiu Zhang, Catharina W. J. Knol, Angelina Osipyan, Nad'a Majerníková, Tingting Chen, Zhangping Xiao, Jeaunice Adriana, Andrew J. Griffith, Abel Soto Gamez, Petra E. van der Wouden, Robert P. Coppes, Amalia M. Dolga, Hidde J. Haisma, Frank J. Dekker

**Affiliations:** ^1^ Department of Chemical and Pharmaceutical Biology Groningen Research Institute of Pharmacy (GRIP) University of Groningen Antonius Deusinglaan 1 Groningen 9713 AV The Netherlands; ^2^ Research School of Behavioural and Cognitive Neuroscience University of Groningen Groningen 9713 AV The Netherlands; ^3^ Department of Pathology and Medical Biology University Medical Centre Groningen University of Groningen Groningen 9713 GZ The Netherlands; ^4^ Department of Molecular Pharmacology Groningen Research Institute of Pharmacy University of Groningen Groningen 9713 AV The Netherlands; ^5^ Department of Biomedical Sciences of Cell & Systems Section Molecular Cell Biology University Medical Center Groningen University of Groningen Groningen 9712 CP The Netherlands; ^6^ Department of Radiation Oncology University Medical Center Groningen Hanzeplein 1 Groningen 9713 GZ Netherlands

**Keywords:** DNA repair, ferroptosis, homologous recombination, inhibitor, macrophage migration inhibitory factor (MIF)

## Abstract

Ferroptosis is a form of regulated cell death that can be modulated by small molecules and has the potential for the development of therapeutics for oncology. Although excessive lipid peroxidation is the defining hallmark of ferroptosis, DNA damage may also play a significant role. In this study, a potential mechanistic role for MIF in homologous recombination (HR) DNA repair is identified. The inhibition or genetic depletion of MIF or other HR proteins, such as breast cancer type 1 susceptibility protein (BRCA1), is demonstrated to significantly enhance the sensitivity of cells to ferroptosis. The interference with HR results in the translocation of the tumor suppressor protein p53 to the mitochondria, which in turn stimulates the production of reactive oxygen species. Taken together, the findings demonstrate that MIF‐directed small molecules enhance ferroptosis via a putative MIF‐BRCA1‐RAD51 axis in HR, which causes resistance to ferroptosis. This suggests a potential novel druggable route to enhance ferroptosis by targeted anticancer therapeutics in the future.

## Graphical abstract

The development of therapeutics to modulate ferroptosis has potential for oncology. Although excessive lipid peroxidation is the defining hallmark of ferroptosis, DNA damage may also play a role. Here, the data demonstrate that MIF‐ and homologous recombination (HR)‐directed small molecules enhance ferroptosis via a putative MIF‐BRCA1‐RAD51 axis in HR, which suggests a potential novel druggable route to enhance ferroptosis.

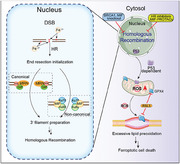

## 1 Introduction

Ferroptosis is a type of programmed cell death^[^ ^1^ ^]^ that has gained significant attention in recent years due to its role in a number of human diseases, including neurodegenerative diseases, organ injury, and cancers.^[^ ^2^, ^3^ ^]^ A key process in ferroptosis is the iron (II) catalyzed nonenzymatic Fenton reaction to provide lipid peroxides.^[^ ^4^ ^]^ It is commonly perceived that high levels of lipid peroxides cause membrane rupture and subsequent cell death.^[^ ^5^ ^]^ Ferroptosis represents a unique form of regulated cell death (RCD) that differs from parthanatos, apoptosis, and necroptosis.^[^ ^1^ ^]^ Moreover, it does not appear to be sensitive to the development of chemotherapy resistance.^[^ ^6^ ^]^ Therefore, the induction of ferroptosis, by the inhibition of its protection systems, holds promise to overcome therapy resistance in cancer. To date, several ferroptosis defense systems have been elucidated. First, glutathione peroxidase 4 (GPX4) utilizes glutathione (GSH) to reduce excessive lipid peroxidation.^[^ ^1^, ^7^, ^8^ ^]^ This process is initiated by the biosynthetic conversion of cysteine to GSH, which can be generated from cysteine. System X_c_^−^ is a cystine/glutamate antiporter that facilitates the uptake of extracellular cystine and enhances GSH biosynthesis, thus contributing to ferroptosis suppression.^[^ ^9^, ^10^ ^]^ Second, ferroptosis suppressor protein 1 (FPS1) inhibits ferroptosis by generating two natural antioxidants, ubiquinol (CoQQH2) and vitamin K hydroquinone (VKH_2_).^[^ ^11^, ^12^, ^13^ ^]^ Third, GTP cyclohydrolase 1 (GCH1) antagonizes ferroptosis by its metabolite, tetrahydrobiopterin (BH_4_).^[^ ^14^ ^]^ To fully exploit the role of ferroptosis induction, the identification of novel ferroptosis enhancers enables the investigation of the molecular mechanisms involved and provides new modalities for drug discovery.

A crucial process in ferroptosis is Fenton chemistry,^[^ ^15^ ^]^ which initiates lipid peroxidation. In addition to lipid peroxidation, the formation of oxidative adducts^[^ ^16^ ^]^ and strand breaks^[^ ^17^ ^]^ is a further consequence of Fenton chemistry. The Fenton reaction is catalyzed by iron, which is increased in ferroptotic cells as a consequence of the activation of ferritinophagy^[^ ^18^ ^]^ and dysregulation of iron metabolism.^[^ ^19^ ^]^ In this context, it is reasonable to presume a mechanistic link between Fenton chemistry, DNA damage, and ferroptosis, which justifies the hypothesis that DNA damage repair mechanisms counteract ferroptosis.

Our understanding of DNA damage repair has grown considerably over recent years. Homologous recombination (HR) is a highly accurate mechanism for repairing DNA damage,^[^ ^20^ ^]^ in which the breast cancer type 1 susceptibility protein (BRCA1) and the DNA repair protein RAD51 homolog 1 (RAD51) play indispensable roles. In contrast, nonhomologous end joining (NHEJ) is a rapid but imprecise DNA repair pathway that may result in chromosomal aberrations and other errors in the repaired DNA.^[^ ^21^ ^]^ The TP53‐binding protein 1 (53BP1) facilitates NHEJ by impeding the resection of DNA double‐strand breaks (DSB).^[^ ^22^, ^23^ ^]^ DNA ligase IV (LIG4) is responsible for the final step of NHEJ, namely ligation.^[^ ^24^ ^]^ Base excision repair (BER) is a cellular mechanism that repairs DNA adducts. The generation of an apurinic/apyrimidinic (AP) site by AP endonuclease 1 (APE1) is a vital step in BER.^[^ ^25^ ^]^ Overall, in this study, we aim to identify DNA damage repair pathways that enable protection against ferroptosis. This provides opportunities to develop inhibitors for these routes of protection in drug discovery projects in oncology.

The repair of DNA damage requires the coordinated action of a number of proteins that are implicated in the binding, cleavage, and ligation of DNA. A growing number of such proteins are currently being identified. A recent addition to the proteins involved in DNA processing is macrophage migration inhibitory factor (MIF). MIF has recently been identified as a nuclease, which belongs to the large PD‐D/E(X)K nuclease superfamily and has both 3′‐5′ exonuclease and endonuclease activity.^[^ ^26^, ^27^, ^28^ ^]^ The primary function of MIF as a nuclease has been demonstrated through the binding to apoptosis‐inducing factor (AIF) and subsequent cleavage of DNA in parthanatos.^[^ ^26^ ^]^ This has led to the suggestion that this process may represent a druggable target in the context of Parkinson's disease.^[^ ^28^ ^]^ Moreover, the MIF protein has been demonstrated to interact with mutated forms of the DNA polymerase δ, thereby facilitating DNA replication by restoring the proofreading ability of the replisome.^[^ ^27^ ^]^ The results of recent studies indicate that MIF may play a role in DNA processing. However, no direct function in DNA repair has been described. A DNA repair protein, Mre11, exhibits a comparable 3′‐5′ exonuclease activity to that described for MIF, which is essential for HR, particularly during the processing of end resection.^[^ ^29^, ^30^ ^]^ It is of interest to determine whether MIF could serve as a functional alternative protein to Mre11. This provides a novel avenue for further investigation into the potential roles of MIF proteins, which have traditionally been identified as a pleiotropic extracellular cytokine.^[^ ^31^ ^]^

The present study employs a range of DNA damage repair inhibitors and small molecule MIF modulators in order to ascertain which DNA repair pathways are capable of counteracting ferroptosis and to investigate the potential involvement of MIF in DNA repair. The aim of this study was to investigate the response of cells to ferroptosis when different DNA repair pathways are disrupted through the use of small molecular inhibitors, in addition to the application of MIF proteolysis targeting chimeras (PROTACs). Our findings indicate that only HR inhibitors and MIF‐PROTACs enhance ferroptosis, which is confirmed by genetic deletions of BRCA1 or MIF. We also demonstrated that HR inhibitors and the MIF‐PROTACs suppress homology‐driven repair in reporter cell lines. The data indicate the presence of a MIF‐dependent DNA repair mechanism (MIF‐BRCA1‐RAD51 axis) that confers resistance to ferroptosis. Furthermore, we were able to identify a small molecule inhibitor of this particular function of MIF that enhances ferroptosis upon GPX4 inhibition. This suggests that this specific MIF function may be a promising target for the development of novel anticancer therapeutics, with the potential application for indications such as triple‐negative breast cancer (TNBC).

## 2 Results and Discussion

### 2.1 Homologous Recombination (HR) but Not Nonhomology end Joining (NHEJ) is Indispensable for Cellular Protection Against Ferroptosis

**Figure 1:**
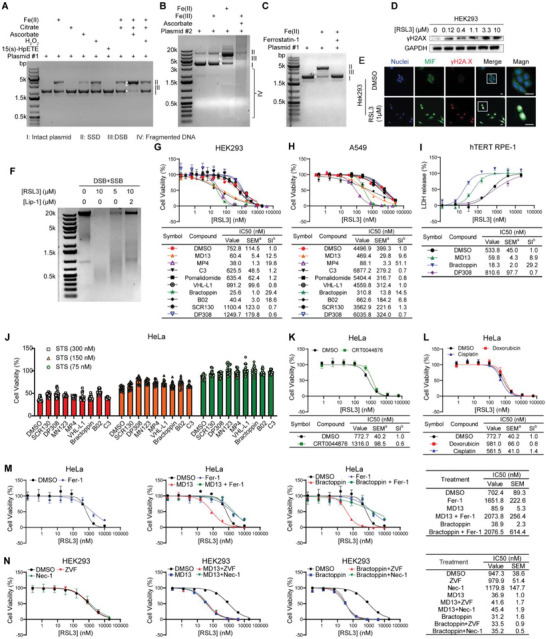
Homologous recombination regulates ferroptosis resistance. A,B) Double‐strand DNA damage induced by iron (II)‐mediated the Fenton reaction in vitro. Samples were separated by an agarose gel. The concentration of each component in the reaction mixtures: plasmid 400 ng, Fe (II) 200 µm, citrate 1 mm; sodium ascorbate 200 µm, H_2_O_2_ 200 µM, 15(s)‐HpETE 30 µm. The incubation periods were 30 min A) and 60 min B), respectively. C) Agarose gel electrophoresis of Fer‐1 inhibition of iron (II)‐mediated double‐strand DNA damage. D) Immunoblotting analysis of the level changes of γH2A.X, a DSB biomarker, upon treatment of RSL3. E) Confocal images of the upregulated γH2A.X level and the nuclear translocation of MIF after RSL3 treatment. The white color in the merged pictures indicates the overly of MIF, γH2A.X, and DAPI in the nucleus. Scale bars, 15 µm. Images are from one representative of three independent experiments. F) Agarose gel electrophoresis of RLS3‐induced genome DNA damage inhibited by Lip‐1. G,H) Dose‐dependent toxicity of RSL3 in HEK293 G) and A549 H) treated with or without indicated inhibitors (10 µm) or PROTACs (10 µm). I) Lactate dehydrogenase (LDH) released by hTERT RPE‐1 cells for analysis of dose‐dependent cytotoxicity of RSL3 combined with or without indicated compounds (10 µm). J) Dose‐dependent toxicity of staurosporine (STS) in HeLa treated with or without indicated inhibitors (10 µm) or PROTACs (10 µm). K) Dose‐dependent toxicity of RSL3 in HeLa treated with or without a BRE inhibitor, CRT0044876 (10 µm). L) Dose‐dependent toxicity of RSL3 in HeLa treated with or without doxorubicin (500 nm) or cisplatin (10 µm). M) The antiferroptosis efficiency of Fer‐1 in cells treated with RSL3 only I) or in combination with MD13 J) or bractoppin K). M) The antideath efficiency of ZVF and Nec‐1 in cells treated with RSL3 only L) or in combination with MD13 M) or bractoppin N). G–N) Data are mean ± SEM. ^a^SEM, standard error of the mean; ^b^SI, sensitizing index. n≥3. A–F) Uncropped images are shown in Figures S7 and S8, (Supporting Information).

The objective of this study was to investigate the relationship between DNA damage and ferroptosis. To this end, the role of iron and ferroptosis inducers in DSB was examined. In accordance with the previous literature,^[^ ^17^ ^]^ iron(II) was found to induce double‐strand DNA damage in vitro, whereas iron(III) did not. The addition of a reductant, such as sodium ascorbate (**Figure** **1**A,B; Figure S1A,B, Supporting Information) was found to enhance this effect. However, the influence of iron(II) was attenuated by a superoxide, 15(s)‐HpETE, or a radical‐trapping antioxidant (RTA),^[^ ^32^ ^]^ ferrostatin‐1 (Figure 1C). In cells, ferroptosis inducers stimulated DSB in a dose‐dependent manner (Figure 1D; Figure S1C, Supporting Information). Confocal microscopy revealed that following treatment with the GPX4 inhibitor RSL3, the γH2A.X level was elevated (Figure 1E) in comparison to the untreated group, suggesting that RSL3 induces DSB in cells. Moreover, the cellular DNA damage induced by RSL3 was attenuated by Lip‐1, a well‐characterized ferroptosis inhibitor (Figure 1F). Surprisingly, MIF was translocated to the nucleus and co‐localized with γH2A.X in the RSL3‐treated group (Figure 1E), which indicates that MIF might play a role in DNA damage in ferroptosis. The collective data indicate that ferroptosis may contribute to DSB in cells, with MIF potentially playing a role in this process.

As a subsequent step, we investigated the potential of modulating various DNA damage repair pathways to protect against ferroptosis. To this end, we employed the following small molecule inhibitors that interfere with DNA repair pathways (**Table** **1**). Bractoppin^[^ ^33^ ^]^ and B02^[^ ^34^ ^]^ are two HR inhibitors, which inhibit the recruitment of BRCA1 to DNA breaks and disrupt the binding of RAD51 to DNA, respectively. SCR130^[^ ^35^ ^]^ is a specific ligase IV inhibitor with 20‐fold higher potency than SCR7 in cells. DP308^[^ ^36^ ^]^ is a 53BP1 inhibitor that interferes with the recruitment of 53BP1 to DSB sites. In addition, MIF‐directed PROTACs were employed to assess the impact of MIF downregulation on ferroptosis protection. Toward this aim, we employed the previously developed MIF‐PROTAC MD13,^[^ ^37^ ^]^ which contains a Cereblon (CRBN) E3 ligase ligand. Meanwhile, we have developed a novel series of MIF‐PROTACs bearing the VHLL1 E3 ligase ligand (Figure S1D, Supporting Information). The VHL‐L1‐based MIF‐PROTACs were used to revalidate the results acquired with the pomalidomide‐based MIF‐PROTAC, MD13. Furthermore, the CRBN ligand in MD13 exhibits fluorescence properties, which precludes the utilization of fluorescence‐based detection techniques, such as flow cytometry and confocal microscopy. The VHL‐L1‐based MIF‐PROTACs lack intrinsic fluorescence, which enables their use in assays based on fluorescence‐based detection. The MIF degradation efficiency of VHL‐MIF‐PROTACs (Figure S1E,F, Supporting Information) was validated by immunoblotting, which demonstrated intracellular MIF degradation at 2 µm. The use of HR inhibitors, such as bractoppin and B02, as well as MIF‐PROTACs, MD13, and MP4, has been demonstrated to effectively sensitize cells to ferroptosis in a range of cell lines, including HEK293 and other carcinoma or noncarcinoma cell lines (Figure 1G,H; Figure S1G–J, Supporting Information). The sensitizing index (SI) was found to be greater than ten in the majority of cases. The ferroptosis‐enhancing effect of the aforementioned compounds has been revalidated with the use of another ferroptosis inducer, ML210, which demonstrated a similar enhancing profile (Figure S1K,L, Supporting Information). Moreover, the LDH release assay indicated that the ferroptotic enhancing effects of the HR inhibitors and the MIF‐PROTACs were comparable. (Figure 1I). In contrast, the NHEJ inhibitors, SCR130 and DP308, demonstrated no sensitizing effects in the majority of cell lines, and in fact, exhibited protective effects against ferroptosis in cell lines, such as in HEK293 (Figure 1G) and hTERT RPE1 (Figure S1H, Supporting Information). The antiferroptosis effects of SCR130 and DP308 may be explained by a mechanism in which the inhibition of NHEJ promotes HR.^[^ ^38^, ^39^, ^40^ ^]^ The MIF tautomerase inhibitor compound 3,^[^ ^37^, ^41^ ^]^ the CRBN ligand pomalidomide, and the VHL ligand VHL‐L1 were employed as control compounds for MIF‐PROTACs. All of the compounds demonstrated no effects on all tested cell lines. In contrast to ferroptosis, HR inhibitors, MIF‐PROTACs, and NHEJ inhibitors were unable to enhance staurosporine (STS)‐induced apoptosis (Figure 1J). Moreover, a specific base excision repair (BRE) inhibitor,^[^ ^42^ ^]^ CRT0044876, did not exhibit any ferroptotic cell death‐enhancing effect (Figure 1K), suggesting that DNA adducts may not be a lethal type of DNA damage in ferroptosis, although ROS‐induced DNA adduct the formation is widely reported. Furthermore, the combination of doxorubicin and cisplatin with RSL3 did not result in a greater degree of cell death (Figure 1L), indicating that cross‐linking DNA strands and inhibiting DNA replication has no significant acute impact on ferroptosis. Together, these results indicate that HR DNA damage repair and the MIF protein play essential roles in protecting cells from ferroptosis, whereas the contribution of BER and NHEJ damage repair and DNA crosslinking appears to be relatively limited.

**Table 1:** The list and description of the main compounds used in this paper.

Compound				
Full name	Abbreviation	Target	Description	Reference
B02		DNA repair protein RAD51 homolog 1 (RAD51)	Homologous recombination (HR) inhibitor	
Bractoppin		Breast cancer type 1 susceptibility protein (BRCA1)	HR inhibitor	
Cisplatin		DNA	Binding to DNA and inhibiting DNA replication	
Compound 3	C3	Macrophage migration inhibitory factor (MIF)	Competitive MIF tautomerase inhibitor (a control for MD13 and MP3)	
CRT0044876		Apurinic/Apyrimidinic endonuclease 1 (APE1)	Base excision repair (BER) inhibitor	
Doxorubicin		DNA	Binding to DNA and inhibiting DNA replication	
DP308		p53‐binding protein 1 (53BP1)	Non‐homologous end joining (NHEJ) inhibitor	
Erastin		System xc‐ cystine/glutamate antiporter	Ferroptosis inducer	
Ferrostatin‐1	Fer‐1	Reactive Oxygen Species (ROS)	Radical‐trapping antioxidant (RTA)	
Liproxstatin‐1	Lip‐1	Reactive Oxygen Species (ROS)	RTA	
MD13		MIF	MIF proteolysis targeting chimera (PROTAC) (pomalidomide‐based)	
MP1‐MP5		MIF	MIF PROTAC (VHL‐L1‐based)	This paper
MN123		MIF	MIF allosteric tautomerase inhibitor	This paper
Necrostatin‐1	Nec‐1	Receptor‐interacting protein kinase 1 (RIPK1)	Necroptosis inhibitor	
Pomalidomide		Cereblon (CRBN)	Ligand of CRBN (a control for MD13)	^]^
RSL3		Glutathione peroxidase 4	Ferroptosis inducer	^]^
SCR130		DNA ligase 4 (LIG4)	NHEJ inhibitor	^]^
VHL ligand 1	VHL‐L1	von Hippel–Lindau (VHL)	Ligand of VHL (a control for MP3)	^]^
Z‐VAD(OMe)‐FMK	ZVF	pan‐caspase	Apoptosis inhibitor	

Four inhibitors targeting either ferroptosis, apoptosis, or necroptosis were employed to distinguish between different types of cell death. The inhibitors were applied in combination with the HR inhibitors and MIF‐PROTACs to ascertain whether enhanced cell death was caused by ferroptosis or the activation of other regulated cell death pathways (RCD). Two ferroptosis inhibitors, Fer‐1 and Lip‐1,^[^ ^32^ ^]^ effectively rescued cells from ferroptosis to similar levels in both the RSL3 single treatment and combination treatment groups (Figure 1M; Figure S1M, Supporting Information). However, ZVF^[^ ^43^ ^]^ and nec‐1^[^ ^44^ ^]^ failed to rescue cells from any treatment (Figure 1N). Besides, the cytotoxicity analysis demonstrated that the tested compounds were nontoxic to cell lines (Figure S1N,O, Supporting Information). These data indicate that HR inhibitors and MIF‐PROTACs increased cell vulnerability toward RSL3 via ferroptosis, rather than the activation of apoptosis or necroptosis.

### 2.2 MIF and Other HR Proteins Have Protective Roles in Ferroptosis

**Figure 2:**
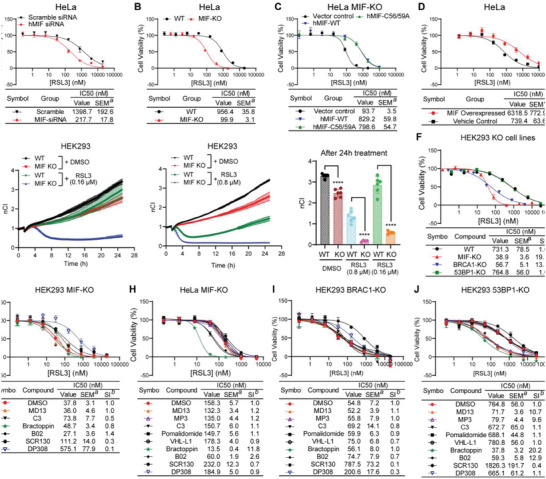
HR proteins play an essential role in the defense against ferroptosis and MIF is on the same pathway as BRCA1 and RAD51. A) Dose‐dependent toxicity of RSL3 in MIF‐siRNA or scramble‐siRNA treated cells. B) Dose‐dependent toxicity of RSL3 in wild‐type (WT) and MIF knockout (MIF‐KO) HeLa. C) Dose‐dependent toxicity of RSL3 in MIF‐rescued and MIF‐KO cells. D) Dose‐dependent toxicity of RSL3 in MIF‐overexpressed HeLa. E) xCELLigence real‐time cell analysis of MIF‐KO and WT cells in response to low (left) or moderate (middle) dose of RSL3. The quantification of xCELLigence data after 24 h treatment of RSL3 (eight). Data are mean ± SEM of *n* = 6 wells of a 96‐well plate from one representative of three independent experiments. G) one‐way ANOVA with Tukey's test. *n* ≥ 5. F) Dose‐dependent toxicity of RSL3 in wild‐type (WT), MIF knockout (MIF‐KO), BRCA1 knockout (BRCA1‐KO), and 53BP1 knockout (53BP1‐KO). G,H) Dose‐dependent toxicity of RSL3 in MIF‐KO HEK293 G) and HeLa H) treated with or without indicated compounds (10 µm). I) Dose‐dependent toxicity of RSL3 in BRCA1‐KO cells treated with or without indicated compounds (10 µm). J) Dose‐dependent toxicity of RSL3 in 53BP1‐KO cells treated with or without indicated compounds (10 µm). A–D, F–J) Data are mean ± SEM. ^a^SEM, standard error of the mean; ^b^SI, sensitizing index. *n* ≥ 3.

The relationship between cellular MIF levels, HR, and susceptibility to ferroptosis was further elucidated through the integration of genetic and chemical methodologies. Toward this aim, siRNA‐mediated MIF knock‐down cells (**Figure** **2**A) and CRISPR/Cas9‐mediated MIF knock‐out cells (Figure 2B,C; Figure S2A, Supporting Information) were generated. RSL3 treatment showed strongly enhanced ferroptosis thus indicating that MIF is essential for protection against ferroptosis. The introduction of MIF plasmids into MIF‐KO cells resulted in the restoration of ferroptosis tolerance of cells (Figure 2C). The overexpression of MIF was found to increase the tolerance of cells to ferroptosis (Figure 2D). However, the exogenous addition of bacterial‐produced recombinant MIF to the culture medium did not afford protection from ferroptosis (Figure S2C, Supporting Information), although it was capable of being taken up into the cytoplasm and even into the nucleus (Figure S2D, Supporting Information). In addition, MIF‐KO cells had a higher background level of DSB than intact cells of two different cell lines (Figure S2A, Supporting Information). MIF‐KO cells were also more prone to induce DSB than wild‐type cells following RSL3 treatment (Figure S2B, Supporting Information). Altogether, these genetic methods show that MIF plays a crucial role in the defense against ferroptosis. This is confirmed by the use of the MIF‐PROTAC, MD9, which serves to reduce cellular MIF levels. This PROTAC exhibits the highest potency at 10 µm (DC_max_) with a DC_50_ of 1.5 µm.^[^ ^49^ ^]^ However, concentrations exceeding 10 µm are less effective and result in the Hook effect.^[^ ^50^ ^]^ A combination of a fixed dose of erastin and a variable dose of MD9 was found to result in a decrease in cell viability, with the lowest viability observed at a concentration of 10 µm. Conversely, an increase in MD9 concentration resulted in a corresponding increase in cell viability (Figure S2E, Supporting Information). Taken together, these data demonstrated that the MIF levels were inversely proportional to the cells’ susceptibility to ferroptosis.

The results were validated using the xCELLigence real‐time cell analysis, which enables the investigation of time‐dependent responses of cells to ferroptosis. In MIF‐WT cells, 0.16 µm of RSL3 was insufficient to induce cell death or to stop cell proliferation. In contrast, in MIF‐KO cells, 0.16 µm of RSL3 induced cell death within 4 h and reached the maximum killing effect within 8 h (Figure 2E, left and right). The cell proliferation was significantly inhibited in the period following 8 h, despite the fact that RSL3 was unable to induce cell death further. When the concentration of RSL3 was increased to 0.8 µm, both MIF‐WT and MIF‐KO cells were killed, although MIF‐KO cells responded faster and more intensely than MIF‐WT cells (Figure 2E, middle and right). Notably, 0.8 µm of RSL3 did not stop the proliferation of MIF‐WT cells. The maximum killing effect of RSL3 in MIF‐WT cells was achieved after ≈6 h, after which the surviving MIF‐WT cells started to proliferate again, in contrast to MIF‐KO cells. In conclusion, MIF‐KO cells responded faster and more strongly to GPX4 inhibition than parental cells, and the effect of GPX4 inhibition lasted much longer.

The MIF‐PROTACs and HR‐targeted inhibitors were further evaluated in knock‐out cell lines to validate the effects and to check for potential off‐target effects. Tests were performed with MIF‐KO, BRCA1‐KO, and 53BP1‐KO (Figure S2F, Supporting Information) cell lines. The MIF‐PROTACs MD13 and MP3 lost their effect on both HEK293 MIF‐KO (Figure 2G) and HeLa MIF‐KO (Figure 2H) cells, thus indicating that the MIF‐PROTACs have no apparent ferroptosis‐related off‐target effects. In HEK293 MIF‐KO cells, both HR inhibitors and the MIF‐PROTAC lost their ability to sensitize cells to ferroptosis, suggesting that MIF may be involved in the same pathway as BRCA1 and RAD51 (Figure 2G). However, in HeLa MIF‐KO cells, the effect of MIF‐PROTACs was abolished, while the effect of HR inhibitors was only attenuated (Figure 2H). This suggests that although MIF, BRCA1, and RAD51 are in the same pathway, there may also be a protein or protein complex in HeLa that substitutes for the function of MIF. Like MIF‐KO cells, BRCA1‐KO cells were more susceptible to ferroptosis than wild‐type cells (Figure 2I). In addition, BRCA1‐KO caused a loss of function of both MIF‐PROTACs and HR inhibitors (Figure 2I), providing further evidence to confirm that MIF, BRCA1, and RAD51 are in the same pathway. In contrast, 53BP1‐KO cells retained the same resistance to ferroptosis as parental cells (Figure 2J). The effect of these compounds on 53BP1‐KO cells was the same as on wild‐type cells. Taken together, these data demonstrate that MIF is involved in a pathway in which BRCA1 and RAD51 play essential roles.

### 2.3 Inhibition of MIF and HR Proteins Induces Overproduction of Superoxide (SOX) in Mitochondria, Resulting in a More Intense Response to GPX4 Inhibition in Cells

**Figure 3:**
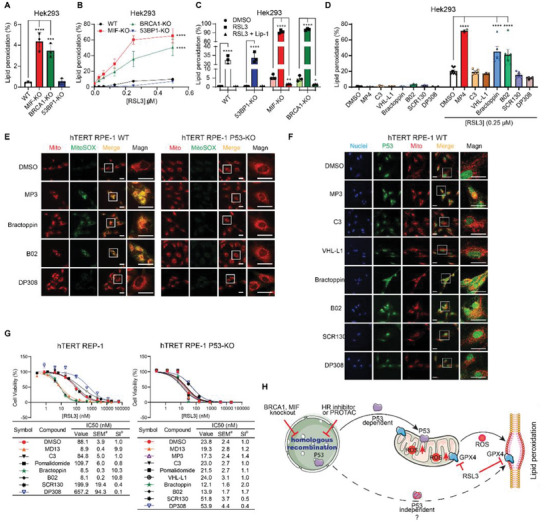
Genetic depletion of HR enhances excessive lipid peroxidation production. A) The background lipid peroxidation level of wild‐type, MIF‐KO, BRCA1‐KO, and 53BP1‐KO cells indexed by BODIPY 581/591 C11 staining. Data are mean ± SEM. ^****^ *p* < 0.0001, one‐way ANOVA with Tukey's test, *n* = 3. B) The lipid peroxidation level of wild‐type, MIF‐KO, BRCA1‐KO, and 53BP1‐KO cells in response to RSL3 treatment (4h). Data are mean ± SEM. ^****^ *p* < 0.0001, two‐way ANOVA with Dunnett's test, *n* = 3. C) The lipid peroxidation level of wild‐type, MIF‐KO, BRCA1‐KO, and 53BP1‐KO cells induced by RSL3 (0.25 µm, 4h) while attenuated by Lip‐1. Data are mean ± SEM. ^****^ *p* < 0.0001, two‐way ANOVA with Dunnett's test, *n* = 3. D) Lipid peroxidation is induced either by only compounds (10 µm) or compounds in combination with RSL3 (0.25 µm, 4h) as indicated. Data are mean ± SEM. ^**^ *p* < 0.0021, ^****^ *p* < 0.0001, two‐way ANOVA with Dunnett's test, *n* ≥ 5. E) Representative images of superoxide over‐production in mitochondria induced by indicated compounds (10 µm, overnight) in hTERT RPE‐1 WT (left) and P53‐KO (right) cells. Scale bars, 30 µm. The yellow color in the merged pictures indicates the co‐localization of mitochondria and mitochondrial SOX. Images are from one representative of three independent experiments. F) Representative images of P53 translocation to mitochondria in hTERT RPE‐1 WT cells treated with indicated compounds (10 µm, overnight). Scale bars, 30 µm. The yellow color in the merged pictures indicates the co‐localization of mitochondria and P53 in the cytoplasm. Images are from one representative of three independent experiments. G) Dose‐dependent toxicity of RSL3 in hTERT RPE‐1 WT (left) and P53‐KO (right) treated with or without indicated compounds (10 µm). Data are mean ± SEM, *n* = 3. ^a^SEM, standard error of the mean; ^b^SI, sensitizing index. H) Graphical abstract depicting that P53 promotes lipid peroxidation in HR deficient cells via translocating to mitochondria, and consequently inducing ROS production.

We then investigated the mechanism by which ferroptosis induction leads to increased cell death in HR and MIF‐deficient cells. To this end, the dye BODIPY 581/591 C11 was used to index lipid peroxidation in several genetically engineered cell lines.^[^ ^51^ ^]^ This shows that MIF‐KO and BRCA1‐KO cells had higher background levels of lipid peroxidation compared to WT or 53BP1‐KO cells (**Figure** **3**A). In addition, RSL3 induced several times more lipid peroxidation in MIF‐KO and BRCA1‐KO cells than in parental or 53BP1‐KO cells under the same conditions (Figure 3B). Importantly, this enhanced lipid peroxidation in KO cell lines was attenuated to background levels by the ferroptosis inhibitor, Lip‐1 (Figure 3C), indicating that HR‐deficiency enhanced lipid peroxidation belongs to ferroptosis, confirming the conclusion derived from cell viability assays (Figure 1M; Figure S1M, Supporting Information). Similar results were observed in the inhibitor treatment experiments. The MIF‐PROTACs and the HR inhibitors, but not the NHEJ inhibitors, enhanced RSL3‐induced lipid peroxidation (Figure 3D; Figure S3A, Supporting Information). Consistent with this, the MIF‐PROTAC and the HR inhibitors enhanced ionizing radiation (IR)‐induced lipid peroxidation (Figure S3B, Supporting Information).

Subsequently, confocal microscopy images showed that MIF‐PROTAC MP3 and HR inhibitors stimulated SOX overproduction in mitochondria (Figure 3E, left). Meanwhile, the mitochondria in these groups shrank to surround the nuclei, indicating inhibitor‐induced mitochondrial damage.^[^ ^52^ ^]^ In contrast, in the negative control and the DP308‐treated groups, mitochondria were distributed in the cytosol with a tubular shape presumably because of their association with microtubules.^[^ ^53^ ^]^ However, the ROS production‐inducing effects of the MIF‐PROTAC and the HR inhibitors were abolished in P53‐KO cells (Figure 3E, right), while the regular tubular‐like distribution of mitochondria remained. In addition, confocal imaging showed that VHL‐MIF‐PROTAC and the HR inhibitors promoted P53 translocation to the mitochondria (Figure 3F; Figure S3C, Supporting Information), a phenomenon that has been described to be responsible for stimulating mitochondrial ROS production.^[^ ^52^ ^]^ As expected, the dose‐dependent GPX4 inhibition by RSL3 shows that the ferroptosis‐promoting effects of the MIF‐PROTACs and the HR inhibitors were attenuated, but not abolished in P53‐KO and P53‐null cells (Figure 3G; Figure S3D, Supporting Information). This provides a Sensitization Index (SI) for the MIF‐PROTACs and the HR inhibitors that was reduced to only 2–3 compared to the above‐mentioned index of ten in normal cells. Taken together, these data show that P53 is an essential responder in HR‐deficient cells in response to ferroptosis inducers, but there are also other P53‐independent pathways that contribute to the residual ferroptosis enhancement in HR‐deficient cells (Figure 3I).

### 2.4 MIF Participates in HR

**Figure 4:**
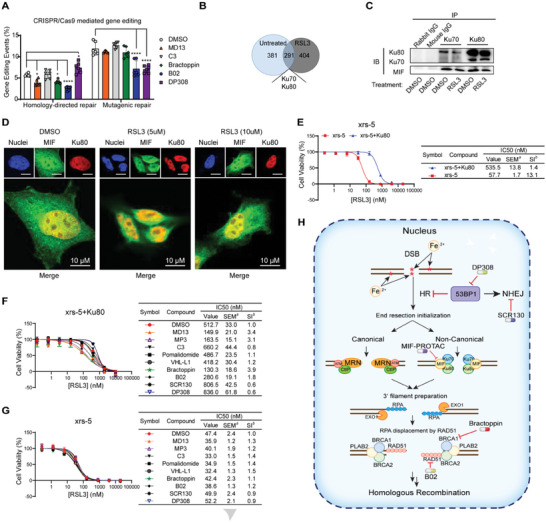
MIF cooperates with Ku70 and Ku80 facilitating HR in ferroptosis. A) Gene editing frequency determined by flow cytometry on H27 treated with indicated compounds (10 µm) and transfected with an EGFP‐sgRNA, Cas9‐WT, and ssDNA template. Data are mean ± SEM. ^*^ *p* < 0.0332, ^**^ *p* < 0.0021, ^***^ *p* < 0.0002, ^****^ *p* < 0.0001, two‐way ANOVA, *n* = 6. B) Mass spectrometry analysis of MIF‐interacting proteins in HeLa. C) Representative immunoblotting of co‐IP of Ku70, Ku80 in HEK293 treated with or without RSL3 (4 µm, 2 h). D) Representative confocal images of co‐localization of MIF, and Ku80 in HEK293 treated with or without RSL3 (4 µm, 2 h). MIF was detected by the antibody. The yellow color (arrowed) in the merged pictures indicates the co‐localization of Ku80, and MIF. E) Dose‐dependent toxicity of RSL3 in Xrs‐5 and Xrs‐5+Ku80 cells. F) Dose‐dependent toxicity of RSL3 in Xrs‐5+Ku80 H) and Xrs‐5 G) treated with or without indicated compounds (10 µm). Data are mean ± SEM, *n* = 3; ^a^SEM, standard error of the mean; ^b^SI, sensitizing index. H) Graphical abstract depicting that MIF collaborates with Ku70/Ku80 to prepare 3’ filament at DSB loci facilitating HR. C,E) Uncropped images were shown in Figures S7 and S10 (Supporting Information).

The above results indicate that MIF participates in the same pathway as BRCA1 and RAD51 and that interfering with the levels of these proteins sensitizes cells to ferroptosis. We then moved on to provide more direct evidence that MIF knockdown affects HR efficiency. Toward this aim, we used the GFP‐to‐BFP conversion assay,^[^ ^53^ ^]^ which is a widely used assay to investigate whether DNA repair efficiency is mediated by homology‐driven repair (HDR) or by other mutagenic repair mechanisms. The results demonstrated that HDR efficiency was significantly decreased upon treatment with the MIF‐PROTAC or the HR inhibitors in two different reporter cell lines (**Figure** **4**A; Figure S4A,B, Supporting Information), indicating that MIF is involved in HR. To understand the molecular mechanism of MIF recruitment to DSB sites, we immunoprecipitated MIF in HeLa cells, followed by mass spectrometry analysis to identify MIF‐interacting proteins (Figure 4B; Figure S4C, Supporting Information). MIF‐KO HeLa was used as a negative control. Among the MIF‐interacting proteins, 291 proteins, including Ku70, and Ku80, were identified under both physiological and ferroptotic conditions. The finding of a MIF/Ku70/Ku80 interaction is consistent with previous literature,^[^ ^27^ ^]^ although previously the function of MIF in this protein complex has not been investigated. To validate the results of the mass spectrometry analysis, we performed co‐IP experiments in HEK293 cells with MIF, Ku70, or Ku80 antibodies, followed by immunoblot analysis (Figure 4C; Figure S4D, Supporting Information). Rabbit and mouse IgG antibodies were used as the negative controls. We found that in all co‐IP experiments, endogenous MIF was associated with endogenous Ku70 and Ku80. Confocal images further confirmed that the MIF/Ku70/Ku80 complex is recruited to DSB sites under ferroptotic and physiological conditions (Figure 4D; Figure S4E,F, Supporting Information). In addition, a Ku80‐null cell line, Xrs‐5, showed an increased sensitivity to GPX4 inhibition compared to a Ku80‐rescued cell line, Xrs‐5+Ku80 (Figure 4E). HR inhibitors and MIF‐PROTACs had no effect on Xrs‐5 cells (Figure 4F). In contrast, the ferroptosis‐promoting effect of these compounds was restored in Xrs5+Ku80 cells (Figure 4G). Taken together, the data suggest that Ku70/Ku80 may be responsible for recruiting MIF to DSB sites to perform end resection initialization to facilitate HR in ferroptosis, which could be a functional alternative complex to replace MRE11‐RAD50‐NBS1 (MRN)‐mediated end resection initialization (Figure 4H).

### 2.5 Identification and Characterization of MIF Allosteric Inhibitor

**Figure 5:**
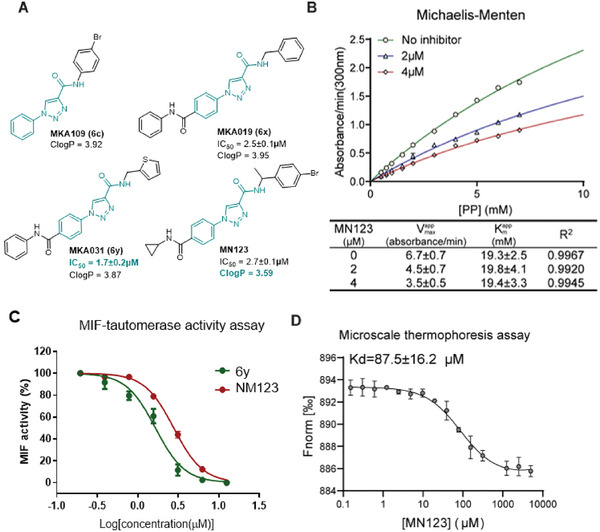
Identification and characterization of a MIF inhibitor MN123. A) Chemical structures of an MIF inhibitor MN123 and MIF allosteric inhibitors described in our previous work. The design of MN123 was inspired by the most active compounds with the goal of improving solubility and cell permeability. B) Michaelis–Menten plots of MIF tautomerase activity in the presence of 0, 2, and 4 µm of MN123. C) Dose‐response curve for inhibition of MIF tautomerase activity by MN123 with an IC_50_ of 2.7 ± 0.1 µm and 6y as a reference. Data are mean ± SEM, *n* = 3. D) Microscale thermophoresis measurements of binding affinity of MN123 to MIF. Form (Normalized fluorescence) = Fhot/Fcold. Data are mean ± SEM, *n* = 2.

As a next step, we set out to identify small molecules that bind to MIF and interfere with its function in ferroptosis. Toward this aim, we used a classical MIF tautomerase activity assay. Using this assay, a compound library containing a 1‐phenyl‐1*H*‐1,2,3‐triazole‐4‐carboxamide core was screened for inhibition. The MIF tautomerase inhibition assay^[^ ^54^ ^]^ was performed and showed that MN123 inhibited MIF tautomerase activity with micromolar potency (**Figure** **5**A,B). The design of compound MN123 was improved over the previous library based on theoretical calculations of ClogP. Presumably, compound MN123 would have better solubility due to the introduction of a methyl group and thus a nonplanar shape compared to 6y (MKA031). Despite comparable IC_50_ values, MN123 showed improved cellular accessibility compared to 6y and, therefore, MN123 was further used in the cell‐based studies. A jump dilution assay was performed to demonstrate that MN123 binds reversibly to MIF (Figure S5A, Supporting Information). Meanwhile, the Michaelis‐Menten plots showed that MN123 noncompetitively inhibited MIF tautomerase activity (Figure 5B). MIF binding was confirmed by MST as an orthogonal assay, this revealed direct binding of MN123 to MIF with a K_d_ of 87.5 µm (Figure 5D).

In addition, we developed a nuclease enzyme activity assay using fluorescence resonance energy transfer (FRET). We employed a single‐strand oligonucleotide (MS33) that was designed as an MIF nuclease substrate according to prior literature.^[^ ^21^ ^]^ However, we have not been able to reproduce previous reports on active site mutants of the putative MIF nuclease active site that lack nuclease activity (Figure S5b‐E, Supporting Information).^[^ ^26^, ^27^, ^28^ ^]^ The MIF nuclease enzyme activity was not consistently present among different batches of the MIF that we produced as compared to the MIF tautomerase enzyme activity (Figure S5b‐A, Supporting Information). This indicates that MIF nuclease enzyme activity and its inhibition cannot be confirmed in this context. Nevertheless, we have characterized MN123 as a new non‐competitive inhibitor of MIF tautomerase activity.

### 2.6 MN123 Enhances Ferroptosis

**Figure 6:**
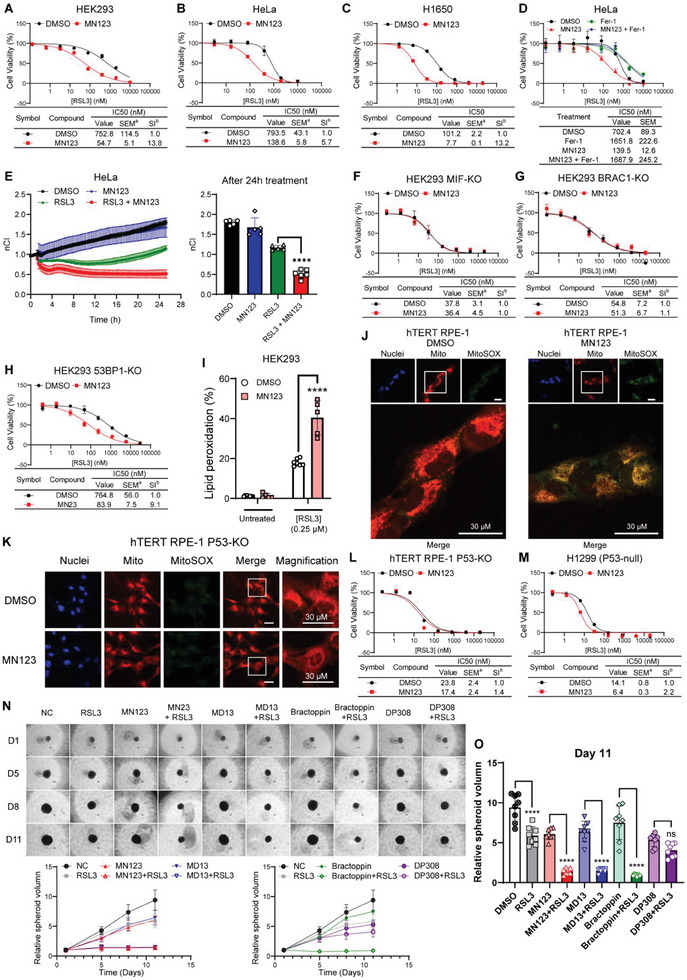
MN123 enhances ferroptosis in various types of cells. A–C) Dose‐dependent toxicity of RSL3 in HEK293 A), HeLa B), and H1650 C) treated with or without MN123 (10 µm). Data are mean ± SEM, *n* ≥ 3. D) The antiferroptosis efficiency of fer‐1 in cells treated with RSL3 or RSL3 in combination with MN123 (10 µm). E) xCELLigence real‐time cell analysis of HeLa (left) in response to RSL3 (4 µm) or RSL3 in combination with MN123 (10 µm). Quantification of xCELLigence data after 24 h treatment (right). Data are mean ± SEM of *n* ≥ 5 wells of a 96‐well plate from one representative of three independent experiments. one‐way ANOVA with Tukey's test. *n* ≥ 5. F–H) Dose‐dependent toxicity of RSL3 in MIF‐KO G), BRCA1‐KO H), 53BP1‐KO I) treated with or without MN123 (10 µm). Data are mean ± SEM, *n* ≥ 3. I) Lipid peroxidation is induced either by MN123 (10 µm) or MN123 in combination with RSL3 (0.25 µm, 4 h). Lipid peroxidation was indexed by BODIPY 581/591 C11 staining. Data are mean ± SEM. ^****^ *p* < 0.0001, two‐way ANOVA with Dunnett's test, *n* ≥ 5. J,K) Representative images of superoxide production in mitochondria induced by MN123 (10 µm, overnight) in hTERT RPE‐1 WT K) and P53‐KO L) cells. Scale bars, 30 µm. The yellow color in the merged pictures indicates the co‐localization of mitochondria and mitochondrial SOX. Scale bars, 30 µm. Images are from one representative of three independent experiments. L,M) Dose‐dependent toxicity of RSL3 in hTERT RPE‐1 P53‐KO L) and H1299 M) treated with or without MN123 (10 µm). Data are mean ± SEM, *n* ≥ 3. N) Representative images and quantifications of 3D spheroid formation assay in HeLa treated with RSL3 (75 nm) with or without indicated compounds (10 µm). Data are mean ± SME, *n* ≥ 7. O) Quantification of M) at day 11. Data are mean ± SME, one‐way ANOVA with Tukey's test, *n* = 8.

The effect of the MIF binder MN123 on ferroptosis was evaluated by analyzing the dose‐dependent enhancement of RSL3 toxicity in several cell lines. MN123 treatment increased the sensitivity to ferroptosis in all cell lines tested by approximately a factor of 10 (**Figure** **6**A–C; Figure S6A,B, Supporting Information), which is comparable to MIF‐PROTACs and HR inhibitors in the same cell lines. The LDH release assay showed that MN123 enhanced the ferroptosis‐induced membrane damage (Figure S6C, Supporting Information). The ferroptosis‐enhancing effect of MN123 is dose‐dependent (Figure S6E,F, Supporting Information) with a measurable effect starting at 0.4 µm and a maximal effect at ≈5 µm. As expected, fer‐1 rescued cells from ferroptosis to the same extent in both RSL3‐only and RSL3+MN123 groups, indicating that MN123 induces more cell death by enhancing ferroptosis (Figure 6D). The xCELLigence real‐time cell analysis results of MN123‐treated HeLa (Figure 6E) were similar to those of MIF‐KO cells (Figure 2E). MIF inhibition induced more ferroptotic cell death. Combination treatment achieved a maximal killing within 10 h, after which the cell proliferation was persistently inhibited for a long time (Figure 6E). Single‐treatment groups with RSL3 or MN123 did not affect cell proliferation and their doubling time did not change compared to the vehicle‐treated group (Figure 6E). MN123 treatment lost its effects in MIF‐KO and BRCA1‐KO HEK293 (Figure 6F,G) while still enhancing ferroptosis in 53BP1‐KO cells (Figure 6H), which is comparable to the observations for the MIF‐PROTACs. Although MN123 had a slightly enhancing effect in MIF‐KO HeLa cells (Figure S6D, Supporting Information), this effect was largely attenuated compared to parental cells (Figure 6B). Taken together, these results indicate that MN123 has a good on‐target ferroptosis‐enhancing effect in various cancer and non‐cancer cell lines.

Next, we aimed to gain a mechanistic understanding of the ferroptosis‐enhancing effect of MN123 Lipid peroxidation staining revealed that MN123 dramatically increased RSL3‐induced lipid peroxidation (Figure 6I). Meanwhile, MN123 treatment stimulated SOX overproduction in mitochondria, resulting in mitochondria shrinkage and damage in P53‐WT (Figure 6J) but not in P53‐KO cells (Figure 6K). Like MIF‐PROTACs, MN123 also stimulated P53 mitochondrial translocation (Figure S6G, Supporting Information). The dose‐dependent toxicity results of RSL3 showed that the enhancing effect of MN123 was weak in P53‐KO and P53‐null cells (Figure 6L,M). Moreover, MN123 inhibited HDR frequency in the GFP‐to‐BFP conversion assay in two different reporter cell lines (Figure S6H,I, Supporting Information). These results indicate that MN123 enhances ferroptosis through HR inhibition and is mediated by P53 mitochondrial translocation, which is consistent with the mechanism demonstrated by HR inhibitors, MIF‐PROTACs, as well as gene‐edited cell lines

Since MIF deletion or inhibition in combination with RSL3 blocks cell proliferation, it is reasonable to evaluate the long‐term effect of the combination treatments. This may indicate whether our findings about HR inhibition and ferroptosis could be translated into a potential anticancer therapy. The long‐term effect was evaluated using a 3D spheroid formation assay, which better mimics tumor growth than in flat‐bottom plates. The results demonstrated that treatment with merely 75 nm of the GPX4 inhibitor RSL3 inhibited ≈40% of tumor growth (Figure 6N,O), while such a low dose of RSL3 showed no cytotoxicity in HeLa cells (Figure 6B). Even stronger effects were observed in the combination treatment groups. Even though the single treatment with either MN123, MD13, or blacktopping inhibited ≈40% of tumor growth, a low dose (75 nm) of RSL3 combined with one of these compounds nearly halted tumor growth (Figure 6N,O). Although inhibition of NHEJ by DP308 slowed down spheroid enlargement similarly to the HR inhibition groups, there is no synergistic effect in the RSL3+DP308 group. Similarly, in MDA‐MB‐231 cells, the combination treatment of MN123 and RSL3 dramatically slowed down tumor spheroid growth (Figure S6J,K, Supporting Information). Starting on day 12, the 3D spheroids in the combination treatment group began to degrade. By day 15, the spheroids had completely disintegrated (Figure S6J, Supporting Information). These data suggest that small‐molecule HR inhibition and treatment with MIF‐PROTACs or the MIF binder MN123 in combination with ferroptosis induction have a strong potential to inhibit the growth of cultured cancer cells.

## 3 Discussion

Ferroptosis was coined in 2012^[^ ^1^ ^]^ and is defined as a form of regulated cell death driven by the iron‐mediated Fenton reaction and characterized by excessive lipid peroxidation. The current paradigm in ferroptosis is that lipid peroxidation causes membrane rupture and cell death. To date, three major ferroptosis defense systems have been identified in mammals, namely GPX4,^[^ ^7^ ^]^ FSP1,^[^ ^12^, ^13^ ^]^ and DHODH.^[^ ^55^ ^]^ Inhibition of these systems results in ferroptosis‐induced cell death in both carcinoma and non‐carcinoma cells.^[^ ^2^, ^5^ ^]^ Complementing the current paradigm, we hypothesize that DNA damage and repair also play a key role in ferroptotic cell death, as iron(II) is known to induce DNA damage as one of the drivers of excessive lipid peroxidation.^[^ ^17^, ^56^ ^]^ Since it has been widely reported that induction of ferroptosis increases the Fe(II) levels in cells,^[^ ^52^, ^57^ ^]^ the possible relationship between iron(II)‐induced DSB and ferroptosis became a focus of our research interest.

In the current paper, by genetically deleting MIF or BRCA1, we showed that MIF is in the same pathway as BRCA1 and RAD51. Degradation of MIF or inhibition of other HR proteins decreased HDR efficiency in reporter cells. Furthermore, co‐IP and confocal microscopy data suggest that MIF/Ku70/Ku80 form a complex that is recruited to DSB sites upon ferroptosis, which may be responsible for end resection and 3’ filament preparation at ferrous‐induced DSB loci and subsequently facilitate the next steps of HR.

Another important finding is that HR, but not NHEJ, is an indispensable protective system against ferroptosis. We have shown that chemically induced degradation of MIF or inhibition of other HR proteins, such as BRCA1 and RAD51, dramatically sensitizes cells to ferroptosis. In contrast, inhibition of NHEJ proteins, such as 53BP1 and LIG4, does not impair and even enhance, the ferroptosis tolerance of cells, which may be because inhibition of NHEJ promotes HR in cells.^[^ ^58^, ^59^ ^]^ The distinct function of HR and NHEJ in ferroptosis is further confirmed by the genetic deletion of BRCA1 and 53BP1, which are proteins of fundamental importance in HR and NHEJ, respectively. Deletion of BRCA1 dramatically sensitizes cells to ferroptosis, whereas knocking out 53BP1 has no effect. A reasonable explanation for these observations is that NHEJ is unable to repair iron(II)‐induced DSBs. The DSB in ferroptosis could be the result of iron‐induced secondary reactions near single‐strand break (SSB) sites,^[^ ^17^, ^60^ ^]^ meaning that the DSB in ferroptosis is not simply a blunt‐end DSB but is surrounded by many SSBs. HR is better equipped to repair this type of DNA damage because it can remove damaged pieces of DNA near DSB sites, accurately repair the breaks, and restore the normal structure of the DNA molecule. The iron(II)‐induced DSB increases the difficulty of repairing by NHEJ but can be repaired by HR. Many reported ferroptosis inhibitors are either antioxidants^[^ ^32^ ^]^ or iron chelators^[^ ^61^ ^]^ that also inhibit iron‐mediated DNA damage. Thus, from the iron side, DNA damage and ferroptosis are closely related.

In the case of apoptosis, HR inhibitors, NHEJ inhibitors, and MIF‐PROTACs failed to enhance staurosporine‐induced apoptotic cell death. This may be explained by differences in DNA fragmentation in both types of cell death. In contrast to ferroptosis, apoptotic DNA fragmentation is mediated by caspase‐activated DNase (CAD), which cleaves genomic DNA into nucleosomal units. Apparently, such DNA damage is beyond the capabilities of the repair mechanisms studied here, which explains the limited effect of DNA repair inhibitors on apoptosis.

This study provides evidence for a mechanistic explanation of the relationship between HR inhibition and increased ferroptosis. HR inhibition stimulated P53 translocation to mitochondria and subsequently promoted excessive superoxide production in mitochondria, which significantly contributed to ferroptosis when GPX4 was inhibited. Previously, Zheng et al. found that mitochondrial P53 induces iron accumulation in mitochondria,^[^ ^52^ ^]^ which explains our observations that the mitochondrial translocation of P53 stimulates ROS production. However, the P53‐dependent pathway is not the only contributor to enhanced ferroptosis upon HR inhibition. Our study also suggests the existence of a P53‐independent mechanism in parallel to the P53‐dependent pathway to enhance ferroptosis in HR‐deficient cells.

We identified and characterized MN123 as an allosteric inhibitor of the MIF tautomerase active site, which significantly enhanced ferroptotic cell death. In contrast, a potent MIF binder that competitively inhibits MIF tautomerase activity, C3,^[^ ^41^ ^]^ failed to enhance ferroptosis. Importantly, MN123 lost its ferroptosis‐enhancing effect in MIF‐KO cells suggesting an on‐target MIF effect. Besides, MN123 inhibited HDR efficiency in reporter cells, which is consistent with the mechanism identified with MIF‐PROTACs and HR inhibitors. The long‐term antiproliferative effects of HR inhibition combined with ferroptosis suggest that pharmacological inhibition of HR and ferroptosis defenders or administration of ferroptosis inducers to HR‐deficient cancers could be an effective anticancer strategy. For example, for triple‐negative breast cancer (TNBC), only one targeted drug has been approved.^[^ ^62^ ^]^ Considering that the functional loss of BRCA1 or BRCA2 is widely identified in patients suffering from TNBC,^[^ ^63^ ^]^ our findings suggest that ferroptosis inducers could be selective against HR‐deficient TNBC, although the therapeutic window should be carefully determined to reduce the potential side effect. As a pleiotropic protein, MIF plays an important role in cancer growth, inflammation, and immune response by activating the signaling pathways of the CD74 and CXC chemokine receptors. Orthosteric MIF inhibitors have been reported to inhibit tumor cell proliferation,^[^ ^41^, ^64^, ^65^ ^]^ and neutrophil recruitment^[^ ^66^, ^67^ ^]^ ″MN123 is an allosteric MIF tautomerase that has yet to be investigated for interfering with the binding of MIF and its extracellular receptors.″ Moreover, future in vivo validation is indispensable to further support the proposed model and move this therapeutic strategy from bench to bedside. At present, there are increasing efforts to develop HR inhibitors as anticancer agents. Most recently, a RAD51 inhibitor, CYT‐0851, is being studied in Phase I/II trials^[^ ^68^ ^]^ as the first HR inhibitor targeting both B‐cell malignancies and advanced solid tumors. The Phase I results showed that CYT‐0851 had an acceptable safety and tolerability profile in cancer patients at the clinical active doses.^[^ ^69^ ^]^ Based on our findings, CYT‐0851 may have synergistic anti‐tumor effects in combination with ferroptosis inducers. In addition, MIF‐targeting molecules, such as MN123 and MIF‐PROTACs, may enhance the HR inhibition efficacy of CYT‐0851 since MIF and RAD51 are involved in the same signaling pathway.

In conclusion, our newly developed allosteric MIF tautomerase inhibitor MN123 and MIF‐PROTACs can enhance ferroptotic cell death and persistently inhibit tumor cell proliferation in combination with a non‐toxic dose of GPX4 inhibitor RSL3. Mechanistically, MIF associates with the Ku70/Ku80 complex and participates in HR to repair DSBs in ferroptosis, thereby counteracting ferroptotic cell death. Interfering with HR by small molecule inhibition or deletion of essential HR genes sensitizes cells to GPX4 inhibition. Upon HR inhibition, P53 was found to be a primary responder to stimulate mitochondrial ROS production, resulting in higher levels of lipid peroxidation when GPX4 was inhibited. These findings provide a rationale for targeting MIF and/or other proteins involved in HR to enhance ferroptosis for the treatment of human cancers.

## Conflict of Interest

The authors declare no conflict of interest.

## Supporting information

Supporting Information

## Data Availability

The data that support the findings of this study are available in the supplementary material of this article.
