# Supplementary material for: Small Molecule MIF Modulation Enhances Ferroptosis by Impairing DNA Repair Mechanisms
Source: Adv Sci (Weinh). 2024 Jun 25;11(32):2403963. doi: 10.1002/advs.202403963 (PMC11348242; doi:10.1002/advs.202403963)

**Small molecule MIF modulation enhances ferroptosis by impairing DNA repair mechanisms**

*Deng Chen^+^,*^[a]^ *Chunlong Zhao^+^,*^[a]^ *Jianqiu Zhang^+^,*^[a]^ *Catharina W.J. Knol,*^[a]^  *Angelina Osipyan,*^[a]^  *Nad’a Majerníková,*^[b][c][d]^ *Tingting Chen,*^[d]^ *Zhangping Xiao,*^[a]^ *Jeaunice Adriana,*^[a]^ *Andrew J. Griffith,*^[a]^ *Abel Soto Gamez,*^[e]^ *Petra E. van der Wouden,*^[a]^ *Robert P. Coppes,*^[e]^ *Amalia M. Dolga,*^[b][d]^ *Hidde J. Haisma,*^[a]^*Frank J. Dekker**^[a]^

**Table of Contents**

1. Experimental Procedures

Chemical Synthesis

Iron-Mediated dsDNA Damage Detection

CRISPR/Cas9-Mediated Gene Knockout

Cell Viability Assays

LDH Release Detection

Impedance-Based Real Time Detection of Cellular Viability

In Vitro MIF Nuclease Assay for End Resection Analysis

Co-Immunoprecipitation (co-IP)

Immunoblotting Analysis

Immunofluorescence Staining

Homology-Driven Repair (HDR) and Mutagenic Repair (MR) Detection

Lipid Peroxidation Detection

Cell Irradiation

3D Spheroid Formation Assay

Recombinant MIF Production and Purification

Microscale Thermophoresis (MST) Assay

MIF Nuclease Activity (MIF-FRET) Assay

MIF Tautomerase Activity Assay

Jump Dilution Assay

2. Supporting figures

Figure S1. Ferroptosis stimulates double-strand break, which mainly relies on homologous recombination to repair.

Figure S2. MIF is important for cells against ferroptosis while bacterial produced recombinant MIF has no such function.

Figure S3. HR inhibition stimulates excessive lipid peroxidation through mitochondrial P53.

Figure S4. MIF cooperates with Ku70 and Ku80 facilitating HR in ferroptosis.

Figure S5. Optimization of the MIF-FRET assay and characterization of MN123.

Figure S6. MN123 enhances ferroptosis in various types of cells.

Figure S7-S10. Uncropped gels and blots for the indicated Figures.

3. The NMR spectra of target compounds

4. The HPLC spectra of target compounds

5. The HRMS spectra of target compounds

**Experimental Procedures**

Chemical Synthesis

Unless otherwise noted, the chemical reagents and solvents were purchased from commercial sources, such as Sigma-Aldrich, Fluorochem as well as Acros, and were used without further purification. All reactions were monitored by thin-layer chromatography (TLC) on 0.25 mm silica gel plates (60GF-254). UV light was used to visualize the spots. ^1^H and ^13^C NMR spectra were recorded on a Bruker DRX spectrometer at 500 MHz, with δ given in parts per million (ppm) and J in hertz (Hz) and using TMS an internal standard. Multiplicity of ^1^H NMR signals was reported as singlet (s), doublet (d), triplet (t), quartert (q), and multiplet (m). High-resolution mass spectra (HRMS) were recorded using Fourier Transform Mass Spectrometry (FTMS) and Orbitrap XL Hybrid Ion Trap-Orbitrap Mass Spectrometer. Silica gel was used for column chromatography purification. C18 reverse-phase high performance liquid chromatography (HPLC) analysis was performed to determine the purity of target compounds.

**Scheme S1**. The synthetic route of compounds **MP1-MP5**

Reagents and conditions: a) TFA/DCM (v:v = 1:2), rt; b) (1) various carboxylic acids, HATU, DIPEA, DMF, rt; (2) LiOH, THF/H_2_O (v:v = 1:1), rt; c) HATU, DIPEA, DMF, rt

***tert-butyl (4-(7-((tert-butyldimethylsilyl)oxy)-2-oxo-2H-benzo[e][1,3]oxazin-3(4H)-yl)phenyl)carbamate (1)*** Compound **1** was prepared according to previously reported methods^32^ and obtained as 0.60 g of pale-yellow solid in a yield of 41%. ^1^H NMR (500 MHz, DMSO-*d*_6_) δ 9.46 (s, 1H), 7.50 (d, *J* = 8.5 Hz, 2H), 7.40 – 7.28 (m, 2H), 7.17 (d, *J* = 8.3 Hz, 1H), 6.69 (dd, *J* = 8.3, 2.4 Hz, 1H), 6.59 (d, *J* = 2.3 Hz, 1H), 4.77 (s, 2H), 1.49 (s, 9H), 0.99 – 0.95 (m, 9H), 0.22 (d, *J* = 0.8 Hz, 6H).

***3-(4-aminophenyl)-7-((tert-butyldimethylsilyl)oxy)-3,4-dihydro-2H-benzo[e][1,3]-oxazin-2-one (2)*** Compound **1** (0.29 g, 0.62 mmol) was dissolved in 2 mL of CH_2_Cl_2_ (DCM) and cooled to 0℃. 1 mL of trifluoroacetic acid (TFA) was added to the solution dropwise. Then, the resulting solution was warmed to room temperature and stirred for 2 h at room temperature. The solution was diluted with DCM (20 mL) and the pH value of the solution was adjusted to 7 by addition of saturated NaHCO_3(aq)._ The organic layer was washed with brine, dried over MgSO_4_, filtered, and concentrated to give compound **2** as 0.19 g of brown solid in a yield of 66%. ^1^H NMR (500 MHz, DMSO-*d*_6_) δ 7.15 (d, *J* = 8.4 Hz, 1H), 7.10 – 7.04 (m, 2H), 6.72 – 6.66 (m, 1H), 6.60 (d, *J* = 8.6 Hz, 2H), 6.56 (d, *J* = 2.4 Hz, 1H), 5.36 (s, 2H), 4.70 (s, 2H), 0.97 (d, *J* = 0.7 Hz, 9H), 0.22 (d, *J* = 0.8 Hz, 6H).

***General procedures for synthesis of compounds 3a-3e***

Respective carboxylic acid (1.0 mmol, 1.0 eq) and HATU (1.2 eq) were dissolved in 5 mL of DMF and the solution was cooled to 0℃ followed by the addition of DIPEA (3.0 eq). The resulting solution was stirred at 0℃ for 5 mins and compound **2** (1.0 eq) were added. The resulting solution was warmed to room temperature and stirred at room temperature overnight. Then, the solution was diluted with EtOAc, washed with water, 1N HCl_(aq)_, saturated NaHCO_3(aq)_, and brine. The organic layer was dried over MgSO_4_, filtered, and concentrated to give intermediate products, which were used without further purification for next step in which the respective intermediate products (1.0 eq) dissolved in 10 mL of THF/H_2_O (1:1) after which LiOH (2.1 eq) was added. The reaction mixtures were stirred at room temperature overnight. The organic solvents were removed under reduced pressure and the pH value of the resulting residues were adjusted to 4-6 by addition of 1N HCl while cooling on an ice bath. The crude products were collected by filtration.

***4-((4-(7-hydroxy-2-oxo-2H-benzo[e][1,3]oxazin-3(4H)-yl)phenyl)amino)-4-oxobutanoic acid (3a)*** Using the general procedure for the synthesis of compound **3a-3e**, compound **3a** was obtained as of brown solid in a yield of 60% . ^1^H NMR (500 MHz, DMSO-*d*_6_) δ 10.10 (s, 1H), 9.81 (s, 1H), 7.63 (d, *J* = 8.8 Hz, 2H), 7.37 (d, *J* = 8.8 Hz, 2H), 7.08 (d, *J* = 8.4 Hz, 1H), 6.60 (dd, *J* = 8.3, 2.4 Hz, 1H), 6.49 (d, *J* = 2.4 Hz, 1H), 4.74 (s, 2H), 2.60 - 2.57 (m,2H), 2.54 (d, *J* = 5.8 Hz, 2H).

***6-((4-(7-hydroxy-2-oxo-2H-benzo[e][1,3]oxazin-3(4H)-yl)phenyl)amino)-6-oxohexanoic acid (3b)*** Using the general procedure for the synthesis of compound **3a-3e**, compound **3b** was obtained as 95 mg of brown solid in a yield of 62%. ^1^H NMR (500 MHz, DMSO-*d*_6_) δ 9.99 (s, 1H), 9.78 (s, 1H), 7.64 (d, *J* = 8.6 Hz, 2H), 7.39 – 7.34 (m, 2H), 7.08 (d, *J* = 8.4 Hz, 1H), 6.60 (dd, *J* = 8.3, 2.3 Hz, 1H), 6.49 (d, *J* = 2.3 Hz, 1H), 4.74 (s, 2H), 2.33 (t, *J* = 7.2 Hz, 2H), 2.26 (t, *J* = 7.2 Hz, 2H), 1.65 – 1.52 (m, 4H).

***8-((4-(7-hydroxy-2-oxo-2H-benzo[e][1,3]oxazin-3(4H)-yl)phenyl)amino)-8-oxooctanoic acid (3c)*** Using the general procedure for the synthesis of compound **3a-3e**, compound **3c** was obtained as 40 mg of brown solid in a yield of 31%. ^1^H NMR (500 MHz, DMSO-*d*_6_) δ 11.98 (s, 1H), 9.98 (s, 1H), 9.78 (s, 1H), 7.64 (d, *J* = 8.7 Hz, 2H), 7.39 – 7.34 (m, 2H), 7.08 (d, *J* = 8.3 Hz, 1H), 6.60 (dd, *J* = 8.3, 2.4 Hz, 1H), 6.48 (d, *J* = 2.3 Hz, 1H), 4.74 (s, 2H), 2.31 (dd, *J* = 8.9, 5.9 Hz, 2H), 2.21 (t, *J* = 7.4 Hz, 2H), 1.60 -1.58 (m, 2H), 1.51 (t, *J* = 7.3 Hz, 2H), 1.33 - 1.30 (m, 4H).

***10-((4-(7-hydroxy-2-oxo-2H-benzo[e][1,3]oxazin-3(4H)-yl)phenyl)amino)-10-oxodecanoic acid (3d)*** Using the general procedure for the synthesis of compound **3a-3e**, compound **3d** was obtained as 53 mg of brown solid in a yield of 30%. ^1^H NMR (500 MHz, DMSO-*d*_6_) δ 9.98 (s, 1H), 9.80 (s, 1H), 7.64 (d, *J* = 8.9 Hz, 2H), 7.41 – 7.31 (m, 2H), 7.08 (d, *J* = 8.4 Hz, 1H), 6.60 (dd, *J* = 8.3, 2.4 Hz, 1H), 6.49 (d, *J* = 2.3 Hz, 1H), 4.74 (s, 2H), 2.31 (t, *J* = 7.5 Hz, 2H), 2.20 (t, *J* = 7.4 Hz, 2H), 1.63 – 1.56 (m, 2H), 1.50 (t, *J* = 7.0 Hz, 2H), 1.28 (d, *J* = 6.5 Hz, 8H).

***12-((4-(7-hydroxy-2-oxo-2H-benzo[e][1,3]oxazin-3(4H)-yl)phenyl)amino)-12-oxododecanoic acid (3e)*** Using the general procedure for the synthesis of compound **3a-3e**, compound **3e** was obtained as 83 mg of brown solid in a yield of 44%. ^1^H NMR (500 MHz, DMSO-*d*_6_) δ 9.98 (s, 1H), 9.80 (s, 1H), 7.64 (d, *J* = 8.6 Hz, 2H), 7.36 (d, *J* = 8.6 Hz, 2H), 7.20 (d, *J* = 8.5 Hz, 1H), 7.08 (d, *J* = 8.4 Hz, 1H), 6.60 (dd, *J* = 8.3, 2.4 Hz, 1H), 6.51 – 6.47 (m, 2H), 4.74 (s, 2H), 2.31 (t, *J* = 7.5 Hz, 2H), 2.19 (t, *J* = 7.3 Hz, 2H), 1.61 - 1.57 (m, 2H), 1.49 (d, *J* = 7.2 Hz, 2H), 1.32 – 1.23 (m, 12H).

Intermediate **4** and **VHL-L1** were synthesized according to reported methods^59^.

***(2S,4R)-1-[(2S)-2-amino-3,3-dimethylbutanoyl]-4-hydroxy-N-[(1S)-1-[4-(4-methyl-1,3-thiazol-5-yl)phenyl]ethyl]pyrrolidine-2-carboxamide hydrochloride (4)*** Light-green solid, yield: 57%. ^1^H NMR (500 MHz, DMSO-*d*_6_) δ 9.08 (s, 1H), 8.62 (d, *J* = 7.8 Hz, 1H), 8.13 (d, *J* = 5.2 Hz, 3H), 7.46 (d, *J* = 8.1 Hz, 2H), 7.39 (d, *J* = 8.0 Hz, 2H), 4.93 (p, *J* = 7.1 Hz, 1H), 4.55 (t, *J* = 8.4 Hz, 1H), 4.32 (s, 1H), 3.90 (d, *J* = 5.6 Hz, 1H), 3.74 (d, *J* = 11.1 Hz, 1H), 3.50 (dd, *J* = 11.0, 3.9 Hz, 1H), 2.47 (s, 3H), 2.12 (t, *J* = 10.5 Hz, 1H), 1.76 (ddd, *J* = 13.2, 9.3, 4.3 Hz, 1H), 1.39 (d, *J* = 7.0 Hz, 3H), 1.03 (s, 9H).

***(2S,4R)-1-((S)-2-acetamido-3,3-dimethylbutanoyl)-4-hydroxy-N-((S)-1-(4-(4-methylthiazol-5-yl)phenyl)ethyl)pyrrolidine-2-carboxamide (VHL-L1)*** White solid, yield: 26%. ^1^H NMR (500 MHz, DMSO-*d*_6_) δ 8.99 (s, 1H), 8.39 (d, *J* = 7.8 Hz, 1H), 7.90 (d, *J* = 9.3 Hz, 1H), 7.44 (d, *J* = 8.2 Hz, 2H), 7.39 (d, *J* = 8.1 Hz, 2H), 5.11 (d, *J* = 3.6 Hz, 1H), 4.93 (t, *J* = 7.2 Hz, 1H), 4.52 (d, *J* = 9.3 Hz, 1H), 4.43 (t, *J* = 8.1 Hz, 1H), 4.28 (s, 1H), 3.61 (d, *J* = 4.7 Hz, 1H), 2.46 (s, 3H), 2.02 (t, *J* = 10.4 Hz, 1H), 1.89 (s, 3H), 1.80 (ddd, *J* = 12.8, 8.4, 4.6 Hz, 1H), 1.38 (d, *J* = 7.0 Hz, 3H), 0.94 (s, 9H).

***General procedures for synthesis of compounds MP1-MP5***

A solution of respective carboxylic acid (0.28 mmol, 1.0 eq), HATU (1.2 eq) and DIPEA (3.0 eq) in 5 mL of DMF was cooled to 0℃. The resulting solution was stirred at 0℃ for 5 mins and compound **4** (1.0 eq) were added. The resulting solution was warmed to room temperature and stirred at room temperature overnight. Then, the solution was diluted with EtOAc, washed with water, 1 N HCl_(aq)_, saturated NaHCO_3(aq)_, and brine. The combined organic layer was dried over MgSO_4_, filtered, and concentrated to the crude products, which were further purified by column chromatography.

***N^1^-((S)-1-((2S,4R)-4-hydroxy-2-(((S)-1-(4-(4-methylthiazol-5-yl)phenyl)ethyl)carbamoyl)pyrrolidin-1-yl)-3,3-dimethyl-1-oxobutan-2-yl)-N^4^-(4-(7-hydroxy-2-oxo-2H-benzo[e][1,3]oxazin-3(4H)-yl)phenyl)succinimide (MP1)***

Using the general procedure for the synthesis of compounds **MP1-MP5**, compound **MP1** was obtained as 50 mg of brown solid in a yield of 23%. ^1^H NMR (500 MHz, DMSO-*d*_6_) δ 10.06 (s, 1H), 9.80 (s, 1H), 8.99 (s, 1H), 8.41 (d, *J* = 7.8 Hz, 1H), 7.94 (d, *J* = 9.2 Hz, 1H), 7.63 (d, *J* = 8.7 Hz, 2H), 7.45 (d, *J* = 8.2 Hz, 2H), 7.41 – 7.35 (m, 4H), 7.08 (d, *J* = 8.4 Hz, 1H), 6.60 (dd, *J* = 8.3, 2.4 Hz, 1H), 6.49 (d, *J* = 2.3 Hz, 1H), 5.12 (d, *J* = 3.5 Hz, 1H), 4.96 – 4.91 (m, 1H), 4.74 (s, 2H), 4.54 (d, *J* = 9.3 Hz, 1H), 4.44 (t, *J* = 8.0 Hz, 1H), 4.37 (t, *J* = 5.1 Hz, 1H), 4.29 (s, 1H), 2.67 – 2.52 (m, 4H), 2.46 (s, 3H), 2.06 – 1.98 (m, 1H), 1.80 (ddd, *J* = 12.9, 8.6, 4.7 Hz, 1H), 1.39 (d, *J* = 6.9 Hz, 3H), 0.95 (s, 9H). ^13^C NMR (126 MHz, DMSO-*d*_6_) δ 171.56, 171.09, 171.04, 169.93, 158.28, 151.97, 150.53, 150.07, 148.23, 145.16, 138.36, 137.26, 131.59, 130.15, 129.30, 127.06, 126.85, 126.40, 119.70, 112.08, 109.31, 69.26, 59.07, 57.02, 56.52, 50.06, 48.18, 38.11, 36.53, 35.84, 26.90, 22.92, 19.05, 16.46. HRMS (ESI), 783.3176 calcd for C_41_H_47_N_6_O_8_S [M+H]^+^, found 783.3163. Purity: 95.3%.

***N^1^-((S)-1-((2S,4R)-4-hydroxy-2-(((S)-1-(4-(4-methylthiazol-5-yl)phenyl)ethyl)carbamoyl)pyrrolidin-1-yl)-3,3-dimethyl-1-oxobutan-2-yl)-N^6^-(4-(7-hydroxy-2-oxo-2H-benzo[e][1,3]oxazin-3(4H)-yl)phenyl)adipamide (MP2)***

Using the general procedure for the synthesis of compounds **MP1-MP5**, compound **MP2** was obtained as 40 mg of light-yellow solid in a yield of 21%. ^1^H NMR (500 MHz, DMSO-*d*_6_) δ 9.99 (s, 1H), 9.80 (s, 1H), 8.99 (s, 1H), 8.40 (d, *J* = 7.8 Hz, 1H), 7.85 (d, *J* = 9.3 Hz, 1H), 7.65 – 7.62 (m, 2H), 7.44 (d, *J* = 8.3 Hz, 2H), 7.41 – 7.35 (m, 4H), 7.08 (d, *J* = 8.4 Hz, 1H), 6.60 (dd, *J* = 8.3, 2.3 Hz, 1H), 6.49 (d, *J* = 2.3 Hz, 1H), 5.12 (d, *J* = 3.6 Hz, 1H), 4.92 (q, *J* = 7.2 Hz, 1H), 4.74 (s, 2H), 4.53 (d, *J* = 9.3 Hz, 1H), 4.44 (t, *J* = 8.0 Hz, 1H), 4.29 (s, 1H), 3.62 (d, *J* = 4.0 Hz, 2H), 2.46 (s, 3H), 2.34 - 2.28 (m, 3H), 2.22 – 2.14 (m, 1H), 2.02 (t, *J* = 10.1 Hz, 1H), 1.80 (ddd, *J* = 12.9, 8.5, 4.6 Hz, 1H), 1.60 - 1.53 (m, 4H), 1.38 (d, *J* = 7.0 Hz, 3H), 0.95 (s, 9H). ^13^C NMR (126 MHz, DMSO-*d*_6_) δ 172.36, 171.69, 171.10, 170.07, 158.28, 151.97, 150.54, 150.07, 148.23, 145.15, 138.34, 137.33, 131.59, 130.16, 129.33, 127.06, 126.85, 126.41, 119.91, 119.76, 112.03, 109.32, 102.67, 102.55, 69.22, 59.02, 56.89, 55.40, 50.06, 48.18, 48.15, 38.19, 36.70, 35.67, 35.21, 26.95, 25.65, 25.34, 22.87, 16.46. HRMS (ESI), 811.3489 calcd for C_43_H_51_N_6_O_8_S [M+H]^+^, found 811.3471. Purity: 95.3%.

***N^1^-((S)-1-((2S,4R)-4-hydroxy-2-(((S)-1-(4-(4-methylthiazol-5-yl)phenyl)ethyl)carbamoyl)pyrrolidin-1-yl)-3,3-dimethyl-1-oxobutan-2-yl)-N^8^-(4-(7-hydroxy-2-oxo-2H-benzo[e][1,3]oxazin-3(4H)-yl)phenyl)octanediamide (MP3)***

Using the general procedure for the synthesis of compounds **MP1-MP5**, compound **MP3** was obtained as 20 mg of light-yellow solid in a yield of 25%. ^1^H NMR (500 MHz, DMSO-*d*_6_) δ 9.99 (s, 1H), 8.99 (s, 1H), 8.39 (d, *J* = 7.8 Hz, 1H), 7.81 (d, *J* = 9.3 Hz, 1H), 7.64 (d, *J* = 8.5 Hz, 2H), 7.44 (d, *J* = 8.0 Hz, 2H), 7.37 (dd, *J* = 12.3, 8.3 Hz, 4H), 7.08 (d, *J* = 8.4 Hz, 1H), 6.60 (dd, *J* = 8.2, 2.4 Hz, 1H), 6.49 (d, *J* = 2.2 Hz, 1H), 5.12 (s, 1H), 4.96 – 4.87 (m, 1H), 4.74 (s, 2H), 4.53 (d, *J* = 9.3 Hz, 1H), 4.43 (t, *J* = 8.0 Hz, 1H), 4.29 (s, 1H), 3.62 (d, *J* = 3.7 Hz, 2H), 2.46 (s, 3H), 2.31 (t, *J* = 7.5 Hz, 2H), 2.25 (dd, *J* = 10.6, 3.5 Hz, 1H), 2.13 (dt, *J* = 14.5, 7.3 Hz, 1H), 2.02 (t, *J* = 10.3 Hz, 1H), 1.84 – 1.75 (m, 1H), 1.63 – 1.56 (m, 2H), 1.57 – 1.43 (m, 2H), 1.38 (d, *J* = 7.0 Hz, 3H), 1.33 - 1.27 (m, 4H), 0.95 (s, 9H). ^13^C NMR (126 MHz, DMSO-*d*_6_) δ 172.51, 171.80, 171.10, 170.08, 158.30, 151.96, 150.53, 150.07, 148.22, 145.14, 138.37, 137.30, 131.59, 130.15, 129.29, 126.85, 126.39, 119.76, 109.30, 59.01, 56.80, 50.06, 48.16, 35.66, 28.94, 26.92, 16.46. HRMS (ESI), 839.3802 calcd for C_45_H_55_N_6_O_8_S [M+H]^+^, found 839.3794. Purity: 95.2%.

***N^1^-((2S)-1-((4R)-4-hydroxy-2-(((S)-1-(4-(4-methylthiazol-5-yl)phenyl)ethyl)carbamoyl)pyrrolidin-1-yl)-3,3-dimethyl-1-oxobutan-2-yl)-N^10^-(4-(7-hydroxy-2-oxo-2H-benzo[e][1,3]oxazin-3(4H)-yl)phenyl)decanediamide (MP4)***

Using the general procedure for the synthesis of compounds **MP1-MP5**, compound **MP4** was obtained as 15 mg of light-yellow solid in a yield of 15%. ^1^H NMR (500 MHz, DMSO-*d*_6_) δ 9.97 (s, 1H), 9.79 (s, 1H), 8.99 (s, 1H), 8.38 (d, *J* = 7.8 Hz, 1H), 7.79 (d, *J* = 9.3 Hz, 1H), 7.64 (d, *J* = 8.7 Hz, 2H), 7.44 (d, *J* = 8.2 Hz, 2H), 7.37 (dd, *J* = 12.7, 8.4 Hz, 4H), 7.08 (d, *J* = 8.4 Hz, 1H), 6.60 (dd, *J* = 8.3, 2.3 Hz, 1H), 6.48 (d, *J* = 2.4 Hz, 1H), 5.11 (d, *J* = 3.5 Hz, 1H), 4.92 (t, *J* = 7.3 Hz, 1H), 4.74 (s, 2H), 4.52 (d, *J* = 9.3 Hz, 1H), 4.43 (t, *J* = 8.0 Hz, 1H), 4.29 (s, 1H), 3.61 (d, *J* = 4.8 Hz, 2H), 2.46 (s, 3H), 2.31 (t, *J* = 7.4 Hz, 2H), 2.29 – 2.21 (m, 1H), 2.11 (dt, *J* = 14.4, 7.0 Hz, 1H), 2.06 – 1.98 (m, 1H), 1.84 – 1.76 (m, 1H), 1.59 (d, *J* = 5.1 Hz, 2H), 1.57 – 1.42 (m, 2H), 1.38 (d, *J* = 7.3 Hz, 2H), 1.32 - 1.23 (m, 8H), 0.94 (s, 9H). HRMS (ESI), 867.4115 calcd for C_47_H_59_N_6_O_8_S [M+H]^+^, found 867.4103. Purity: 97.7%.

***N^1^-((S)-1-((2S,4R)-4-hydroxy-2-(((S)-1-(4-(4-methylthiazol-5-yl)phenyl)ethyl)carbamoyl)pyrrolidin-1-yl)-3,3-dimethyl-1-oxobutan-2-yl)-N^12^-(4-(7-hydroxy-2-oxo-2H-benzo[e][1,3]oxazin-3(4H)-yl)phenyl)dodecanediamide (MP5)***

Using the general procedure for the synthesis of compounds **MP1-MP5**, compound **MP5** was obtained as 20 mg of light-yellow solid in a yield of 15%. ^1^H NMR (500 MHz, DMSO-*d*_6_) δ 9.97 (s, 1H), 9.79 (s, 1H), 8.99 (s, 1H), 8.39 (d, *J* = 7.8 Hz, 1H), 7.80 (d, *J* = 9.4 Hz, 1H), 7.67 – 7.61 (m, 2H), 7.44 (d, *J* = 8.1 Hz, 2H), 7.37 (dd, *J* = 12.6, 8.5 Hz, 4H), 7.08 (d, *J* = 8.4 Hz, 1H), 6.60 (dd, *J* = 8.2, 2.4 Hz, 1H), 6.48 (d, *J* = 2.2 Hz, 1H), 5.11 (d, *J* = 3.6 Hz, 1H), 4.97 – 4.84 (m, 1H), 4.74 (s, 2H), 4.52 (d, *J* = 9.3 Hz, 1H), 4.43 (t, *J* = 8.0 Hz, 1H), 4.28 (d, *J* = 6.0 Hz, 1H), 3.61 (d, *J* = 4.6 Hz, 2H), 2.46 (s, 3H), 2.31 (t, *J* = 7.5 Hz, 2H), 2.29 – 2.20 (m, 1H), 2.11 (dt, *J* = 14.0, 7.0 Hz, 1H), 2.06 – 1.98 (m, 1H), 1.80 (ddd, *J* = 13.0, 8.7, 4.7 Hz, 1H), 1.59 (d, *J* = 7.5 Hz, 3H), 1.55 – 1.42 (m, 2H), 1.38 (d, *J* = 7.0 Hz, 2H), 1.30 - 1.26 (m, 12H), 0.94 (s, 9H). ^13^C NMR (126 MHz, DMSO-*d*_6_) δ 172.53, 171.81, 171.09, 170.07, 158.28, 151.98, 151.94, 150.54, 150.07, 148.23, 145.15, 138.37, 137.30, 131.59, 130.16, 129.30, 127.07, 126.85, 126.40, 119.77, 109.32, 69.22, 59.05, 56.75, 50.06, 48.17, 38.24, 36.86, 35.66, 35.36, 29.38, 26.91, 25.92, 25.57, 22.91, 16.45. HRMS (ESI), 895.4428 calcd for C_49_H_63_N_6_O_8_S [M+H]^+^, found 895.4419. Purity: 95.0%.

***Compound DP308 was synthesized according to reported methods***^31^***.***

***(E)-N-(2-(diethylamino)-4-methylquinolin-6-yl)-3-(furan-2-yl) acrylamide (DP308)***

^1^H NMR (500 MHz, Chloroform-*d*) δ 10.08 (s, 1H), 8.05 (s, 1H), 7.83 (d, *J* = 9.0 Hz, 1H), 7.65 (d, *J* = 9.0 Hz, 1H), 7.54 (s, 1H), 7.43 (d, *J* = 15.4 Hz, 1H), 6.67 – 6.59 (m, 3H), 6.52 (d, *J* = 3.4 Hz, 1H), 3.63 (s, 4H), 2.60 (s, 3H), 1.30 (t, *J* = 7.3 Hz, 6H). HRMS (ESI+), 350.1863 cal. for C_21_H_23_N_3_O_2_ [M+H]^+^, found 350.1854.

**Scheme S2**. The synthetic route of compound **MN123**

Reagents and conditions: a) DCC, acetonitrile, rt; b) HBF_4_, NaNO_2_, NaN_3_, H_2_O, rt; c) CuSO_4_*5H_2_O, sodium ascorbate, H_2_O/MeOH (v:v = 1:10), rt

***(S)-N-(1-(4-bromophenyl)ethyl)propiolamide (7)*** Compound **5** (122 μL, 2.0 mmol) and *N,N*′-dicyclohexylcarbodiimide (DCC, 413 mg, 2.0 mmol) were dissolved in dry acetonitrile (10 mL) and cooled in an ice bath for 15 min. Then, compound **6** (400 mg, 2.0 mmol) was added and the resulting solution was stirred at room temperature for 2 hours. The resulting precipitation was removed by filtration. The solvent was removed under reduced pressure and the residue was used without further purification for the next step.

***4-azido-N-cyclopropylbenzamide (9)*** Concentrated HBF_4_ (2 mL) was added dropwise to a suspension of compound **8** (352 mg, 2.0 mmol) in H_2_O (5 mL) over 5 min. After cooling the resulting solution to 0°C, NaNO_2_ (268 mg, 4.0 mmol) was added portion-wise. The mixture was left stirring for 1 hour at room temperature. A freshly-prepared solution of NaN_3_ (325 mg, 5.0 mmol) in 3 mL of H_2_O was added dropwise to the reaction mixture and left stirring for 1 hour at room temperature. The reaction mixture was extracted with diethyl ether (3x20 mL). The combined organic layers were washed with brine, dried with MgSO_4_, filtered through silica gel, and concentrated under reduced pressure. The product was obtained as a yellow solid and used without further purification.

***(S)-N-(1-(4-bromophenyl)ethyl)-1-(4-(cyclopropylcarbamoyl)phenyl)-1H-1,2,3-triazole-4-carboxamide (MN123)*** Compound **7** (1.0 mmol) and compound **9** (1.0 mmol) were dissolved in MeOH (5 mL) each and added into a round bottom flask equipped with a stirring bar. Freshly prepared solutions of CuSO_4_*5H_2_O (25 mg, 0.1 mmol) and sodium ascorbate (40 mg, 0.2 mmol) in 0.5 mL of H_2_O were added to the reaction mixture, and left stirring overnight at room temperature. The volatiles were evaporated, and the residue was diluted with 30 mL of dichloromethane (DCM). A minimal amount of silica gel was added, the mixture was then evaporated under pressure and the product was purified using medium pressure liquid chromatography (MPLC) to afford **MN123** (181 mg, 40%) as a white solid. ^1^H NMR (500 MHz, DMSO-*d_6_*) δ 9.4 (s, 1H), 9.1 (d, J = 8.3 Hz, 1H), 8.6 (d, J = 4.2 Hz, 1H), 8.0 (q, J = 8.6 Hz, 4H), 7.5 (d, J = 8.5 Hz, 2H), 7.4 (d, J = 8.5 Hz, 2H), 5.2 (p, J = 7.2 Hz, 1H), 2.9 (tq, J = 7.8, 4.0 Hz, 1H), 1.5 (d, J = 7.1 Hz, 3H), 0.7 – 0.7 (m, 2H), 0.6 – 0.6 (m, 2H) ppm. ^13^C NMR (126 MHz, DMSO-*d_6_*) δ 166.2, 158.6, 144.0, 143.7, 138.0, 134.6, 131.1, 128.9, 128.5, 125.1, 119.9, 119.7, 47.6, 23.1, 21.9, 5.7 ppm. HRMS (ESI), 454.0873 calcd for C_21_H_21_BrN_5_O_2_ [M+H]^+^, found 454.0874. Purity: 98.9%.

Iron-Mediated dsDNA Damage Detection

Plasmids (400ng) were mixed with Fe(II) or Fe(III) and other indicated components to a desired final concentration. The reaction volume was toped up to 10 µl by adding nuclease-free water. After 30 min incubation at 37ºC, DNA damage was detected either by non-denaturing or denaturing agarose gel electrophoresis as described before^60^. After electrophoresis, the gel was stained with SYBR™ Gold Nucleic Acid Gel Stain (Invitrogen) for 20 min. Images were acquired with a G-BOX imager.

CRISPR/Cas9-Mediated Gene Knockout

Knockout of MIF, BRCA1, and 53BP1 in human cell lines was performed using a CRISPR/Cas9 system. The sequences of sgRNA used in this study are listed below.

MIF-sgRNA, 5’-ATCGCGGTGCACGTGGTCC-3’;

BRCA1-sgRNA, 5’- CACCGTTTCTATCATCCAAAGTAT-3’;

53BP1-sgRNA, 5’- GGACTGCTAGGAACGATAAA-3’;

The plasmids containing sgRNA and Cas9 were transfected into cells with Lipofectamine 2000 (Invitrogen) following the manufacture’s instruction. Cells were selected with puromycin (2 µg/ml, Invitrogen) for 3 days, and then a monoclonal cell line was generated by limiting dilution results in cell populations. Cells were maintained in DMEM with 10% (MIF-KO and 53BP1-KO) or 20% (BRCA1-KO) (v/v) FBS and 1% (v/v) penicillin/streptomycin in a humidified incubator at 37ºC, 5%CO_2_ for 3-4 weeks followed by immunoblotting analysis to verify target gene deletion.

P53-KO hTERT REP-1, xrs-5, xrs-5+Ku80 cell lines were generated and characterized previously^61,62^.

Cell Viability Assays

Cells were seeded in a 96-well plate at a density of 2,400 cells/well with 80 μl of medium. The next day, cells were treated with 10 μl of 10-fold concentrated five-fold serially diluted RSL3. Next, 10 μl of 100 μM compound was added to each well. The untreated control group added the same amount of medium. The final medium volume of each well is 100 μl. After 24 h, 20 μl of CellTirer 96 Aqueous One Solution reagent (Promega) was added to each well and incubated for around 2-3 h according to the manufacturer’s instruction. The optical density (OD) was determined at 490 nm using a Synergy H1 plate reader (BioTek). The untreated group was set to 100% cell viability, whereas the wells in the absence of cells were set to 0% cell viability. Sensitizing index was calculated using the equation listed below.

$$Sensitizing index=\frac{IC50 (RSL3 single treated group)}{IC50 (combination treated group)}$$

LDH Release Detection

hTERT REP-1 cells were seeded in 96-well plates at a density of 4,000 cells/well with 80 μl of medium. The cell treatment was the same as described above in the section on cell viability assays. The untreated control group added the same amount of medium, while the positive control group added 0.2% Triton X-100. After 24 h incubation, treatment medium was replaced by 100 µl of fresh media. Later, cells were lysed and LDH was detected by LDH-Glo™ kit (Promega) according to the manufacture’s instruction. The group in the absence of compounds was considered as 100% cell membrane integrity (0% LDH release), while the wells added Triton-X100 were set to 0% cell membrane integrity (100% LDH release).

Impedance-Based Real Time Detection of Cellular Viability

Cell impedance was measured using xCELLigence system Real-Time Cell Analyzer RTCA-MP (ACEA Biosciences). Normalization of CI values was performed in RTCA Software 1.2 (ACEA Biosciences). Cells were seeded in 96 well plates with gold microelectrodes attached to the bottom of the wells (E-plates, ACEA Biosciences). Attached cells act as electrical insulators and therefore influence the impedance signal. The readout measured by xCELLigence system are arbitrary cell index-values (CI) which can be normalized to 1 at a specific time point. Background signal due to the presence of media was measured before seeding and subtracted by the RTCA software. Finally, cell behavior was not affected by the presence of microelectrodes or the applied electrical signal.

In Vitro MIF Nuclease Assay for End Resection Analysis

dsDNA was prepared by annealing three oligonucleotides (Oligo A, 5’-TAATACGACTCA-CTATAGGGCTCGTGACCACCCTGACCTAGTTTTAGAGCTAGAAATAGCAAGTTAAAAT-AAGGCTAGTCCGTTATCAACTTGAAAAAGTGGCACCGAGTCGGTGCTTTT-3’; Oligo B, 5’-GACTAGCCTTATTTTAACTTGCTATTTCTAGCTCTAAAACTAGGTCAGGGTGGTCA-CGAGCCCTATAGTGAGTCGTATTA-3’; Oligo C, 5’-CGTTATCAACTTGAAAAAGTGGCA-CCGAGTCGGTGCTTTT-3’). Purified MIF protein (10 µM) was incubated with the pre-annealed dsDNA (20 ng) in the nuclease buffer (50 mM Tris-Cl, 5 mM MgCl_2_, pH 8.2) at 37ºC for indicated time. The reaction was terminated by adding Novex™ TBE-Urea Sample Buffer and separated on Novex™ 15% TBE-Urea Gel (Invitrogen). The gel was stained with 1X SYBR™ Gold Nucleic Acid Gel Stain (Invitrogen) for 20 min. The images were acquired with a G-BOX imager.

Co-Immunoprecipitation (co-IP)

Co-IP assay was performed under a native condition as previously described^22^. Cells were harvested by trypsinization and lysed with NETN lysis buffer [150 mM NaCl, 1 mM EDTA, 10 mM Tris-HCl, pH 8.0, 0.5% IGEPAL, and protease inhibitor cocktail (PIC, sigma)] on ice for half an hour. Afterwards, cell debris was pelleted by centrifuging at 15,000 g for 15 min. The supernatant was incubated with indicated antibodies and 25 µl of protein A/G PLUS-agarose beads (Santa Cruz Biotechnology) for overnight at 4ºC. Later, the beads were washed with NETN buffer for five times. The proteins bound on the beads was denatured with 1× SDS loading buffer and separated with a NuPAGE Protein Gel (Thermo Fisher Scientific). The gel was either send for mass spectrometry analysis or immunoblotting analysis.

Immunoblotting Analysis

Cells were lysed with RIPA buffer with 1x PIC, and PhosSTOP™ (sigma, only for phosphorylated protein detection). The samples were sonicated to reduce viscosity. The undissolved cell debris was pelleted by centrifugating at 16,000x g for 30 min at 4ºC. The supernatant was collected and the protein concentration was determined by Pierce™ BCA Protein Assay Kit (Thermo Fisher Scientific). After adding SDS protein loading dye and boiling, 30 µg of protein was separated on a NuPAGE Protein Gel and followed by transferring to a polyvinylidene difluoride (PVDF) membrane. After blocking membrane with 5% (w/v) skim milk, the membrane was incubated with desired primary antibodies (1:1000) at 4ºC for overnight, followed by washing and incubating with HRP-conjugated secondary antibody (1:2000). The blots were lighted up by ECL Select™ Western Blotting Detection Reagent (Cytiva). The images were acquired with a G-BOX imager.

Immunofluorescence Staining

For mitochondria and mitochondrial superoxide detection, cells were seeded at 50,000 cells/well in 24-well plates on glass coverslips, and treated with compounds as indicated. Thirty minutes before the end of the treatment, MitoTracker™ Deep Red FM (ThermoFisher Scientific) and MitoSOX™ (ThermoFisher Scientific) were added to the medium to a final concentration of 200 nM and 1.25 µM, respectively. Next, cells were fixed with 4% PFA for 15 min at RT.

For mitochondria and P53 detection, cells were seeded and treated as described above, but stained with MitoTracker™ Deep Red FM only. After fixation, cells were permeabilized with 2.5% TritonX-100 for 10 min and blocked with 2% BSA in PBS for 1h at RT. Later, cells were incubated with anti-P53 (1:25, MA5-12557, Invitrogen) for 1h at RT. After washing three times with 0.1% PBST, cells were incubated with Goat anti-Mouse Alexa Fluor™ Plus 488 Secondary Antibody (1:1000, A32723, Invitrogen) for 1h at RT.

For MIF, Ku70, Ku80, and γH2AX detection, cells were seeded and treated with RSL3 and the MIF specific fluorescence probe, ZP307 (10 µM), for 3h at 37ºC. After fixation, permeabilization, and blocking, cells were incubated with anti-Ku70, anti-Ku80, and anti- γH2AX antibodies. After intensive washing, cells were treated with proper secondary antibody for 1h at RT.

For all samples, coverslips were mounted onto slides with anti-fading mountant with NucBlue™ stain (Invitrogen). The pictures were acquired using a Leica SP8 confocal laser scanning microscope and analyzed by ImageJ.

Homology-Driven Repair (HDR) and Mutagenic Repair (MR) Detection

To determine if compounds affect the HDR and MR efficiency, a CRISPR/Cas9-mediated EGFP/EBFP conversion platform was employed. HEK.TLR^TetO.KRAB^ cells were seeded at 5x10^5^ cells/well in flat bottom, Cell+, 6-well plates (83.3920.300, SARSTEDT) with 2 ml medium without antibiotics. Before DNA transfection, cells were treated with desired compounds for 24h. The DNA mixture transfection was performed with polyethyleneimine (PEI) as described befor. Briefly, two tubes (A and B) were prepared for each transfection. For tube A, 1.8 µg Cas9 plasmid, 0.6 µg EGFP-gRNA plasmid, and 1 µg ODN.as oligonucleotide were mixed with 220 µl of 150 mM NaCl. For tube B, 18.75 µl of 1mg/ml PEI was diluted in 201.25 µl of 150 mM NaCl. Then, tube B was added dropwise to tube A and gently mixed well. After 20 min incubation at RT, the 400 µl of DNA-PEI complex was directly added to each well. The transfection mixture was replaced by regular culture medium after 6h without doxycycline (dox). At the third day of post-transfection, cells were sub-cultured with regular medium every 3 days for a week. After which, cells were sub-cultured with medium containing 0.2 µg/ml dox for a week to active transgene expression. The frequencies of EGFP- or EBFP-positive cells were determined by flow cytometry.

Lipid Peroxidation Detection

Cells were seeded on 6-well plates. The next day, cells were treated with compounds (10µM) for 3 h. Then, cells were stained with 2 µM of BODIPY™ 581/591 C11 (Invitrogen) for another 1 h at 37 ºC. Later, cells were harvested and washed three times with ice-cold PBS, followed by resuspended in FACS buffer (1% BSA in PBS). Lipid peroxidation was determined by NovoCyte Quanteon Flow Cytometer (Agilent) with a 488nM laser on B530/30 and B586/20 detectors. A minimum of 10k cells were analyzed per sample.

Cell Irradiation

Irradiation was performed using a Cesium-137 source (IBL 637 Cesium-137g-ray machine) with a dose rate of 0.59 Gy/min as previously described^63^. In short, cells were seeded on 6 well plates at a density of 5x10^5^ cells. Three hours prior to irradiation, cells were treated with indicated compounds and irradiated or sham-irradiated up to total doses of 30 Gy. Sham and irradiated cells were later stained with BODIPY™ 581/591 C11 (Invitrogen) for lipid peroxidation determination.

3D Spheroid Formation Assay

Cells were seeded onto a Nunclon™ Sphera™ 96-Well, Nunclon Sphera-Treated, U-Shaped-Bottom Microplate (Thermo Scientific) at a density of 2000 cells/well. After 3 days of incubation, the spheroids were treated with indicated compounds every 3 days. Pictures were acquired and the diameter of each tumor spheroid was determined by an inverted microscope (Nikon Eclipse Ti) with a NIS-elements software. The data were analyzed in GraphPad Prism 8 (Insightful Science).

Recombinant MIF Production and Purification

C-terminal His-tagged recombinant human MIF was expressed with pET-20b(þ) plasmid and Escherichia coli BL21 according to literature procedure^64^. After culturing Escherichia coli cells were pelleted by centrifugation at 4000 rpm for 20 min. The pellet was sonicated in 50 mM sodium shosphate (NaPi) buffer, pH 7.2, 20 mM NaCl, 10% glycerol and centrifuged (swing out) for 1h at 18k rpm and 4⁰C. The supernatant was loaded on combined HiTrap Q HP and HiTrap SP HP ion exchange columns (Cytiva, 2x5mL Q + 5mL SP), equilibrated with 50 mM NaPi buffer (pH 7.2), 20 mM NaCl, and 10% glycerol. The flowthrough, which contained MIF, was collected and 1.5 M pestle crushed ammonium sulphate (AS) was slowly added to the solution. After 1 hour of mixing at 4⁰C, the precipitation was centrifuged (swing out) for 10 min at 10k rpm and 4⁰C. The supernatant was collected and loaded on to a 5 mL Phenyl Sepharose High Performance column (Cytiva), calibrated with 50 mM NaPi buffer and 1.5 M AS, pH 7.8. MIF was eluted from the column with 50 mM NaPi buffer, pH 7.2, 20 mM NaCl, and 10% glycerol. The samples containing MIF were pooled and concentrated to ~5mL with 1K Microsep Advance Centrifugal Devices (swing out centrifuge, 3700 rpm). Subsequently, the sample was loaded on to HiLoad Superdex 75 PG 26/60 (GE Healthcare, USA) size-exclusion column and washed with 20 mM Tris, pH 7.5, 20 mM NaCl. Pierce BCA Protein Assay Kit (Thermo Fisher Scientific) was used to determine protein concentration. The resulting MIF was assessed by SDS gel electrophoresis, and no impurities were observed (>95%). The concentration of MIF was determined by BCA protein assay to be 1 mg/mL (70 µM). The purified protein was aliquoted and stored at -80°C. HRMS (ESI), calcd for monoisotopic mass 14657.3 Da, deconvoluted for monoisotopic mass 14657.2 Da. Deconvolution is done with UniDec ver.5.05.02^65^.

Microscale Thermophoresis (MST) Assay

MST experiments were performed on a Monolith NT.115 system (NanoTemper Technologies) using 100% LED and 40% IR-laser power. Laser on and off times were set at 30 s and 5 s, respectively. The 100 nM dye solution RED-tris-NTA was prepared by mixing 2 µl of the dye (5 µM) and 98 µl PBST. The protein concentration was adjusted to 140 nM, and 100 µl of this solution was added to the tube with dye and incubated at rt for 30 min. Meanwhile a two-fold dilution series of the compound MN132 was prepared in PBST containing 5% DMSO. Subsequently, 10 µl of labelled MIF was mixed with 10 µl of the samples with different concentration of MN123. The samples were centrifuged for 10 min at rt and the supernatant was transferred into fresh tubes. After that the standard treated capillaries (K002) were filled with the solutions and MST was measured.

MIF Nuclease Activity (MIF-FRET) Assay

The nuclease activity of MIF was measured using DNA-template MS33 as a substrate. For the assay, the MIF stock solution (300 μM) was diluted in FRET buffer (100 mM Tris, 5 mM MgCl2, 5mM KCl, pH 8.2) to get 50 nM intermediate solution. The enzyme activity was determined by premixing 50 μl of the MIF dilution with 25 μl of the inhibitors dissolved in FRET buffer. This mixture was pre-incubated for 15 min. Next, 25 μl of 200 nM MS33 solution in FRET buffer was added the mixture. The final concentration of MIF and MS33 was 25 nM and 50 nM, respectively. Subsequently, MIF-nuclease activity was monitored with excitation/emission of 480/520 nm, reflecting the unquenched fluorescence intensity of the substrate (cleaved MS33). The increase in fluorescence was observed over the 50 min of incubation using a BioTek Synergy H1 Hybrid plate reader. Increasing fluorescence signals with a linear slope, typically 10 to 40 min, were used to calculate the reaction velocities. The MIF-nuclease activity in the absence of an inhibitor was set to 100% enzyme activity, while the groups in the absence of MIF was set to 0% activity. The Michaelis–Menten kinetics was detected with the same procedure by different concentrations of MS33. Data were analyzed in GraphPad Prism 8.

MIF Tautomerase Activity Assay

Inhibition of the tautomerase activity and kinetics of MIF was measured using pyruvic acid (PP) as a substrate. A stock solution was prepared by mixing PP in 50 mM ammonium acetate buffer and adjusted to pH 6.0 using 1.0 M NaOH to provide a concentration of 20 mM. This solution was incubated overnight at 37°C to allow equilibration of the keto- and enol- forms and then was stored at 4°C. For the assay, MIF stock solution (10µL, 70 µM) was diluted in 20 mL of the boric acid buffer (435 mM H_3_BO_3_, 1 mM EDTA, pH 6.2) to provide 35nM solution. The enzyme activity was determined by premixing 192 μl of the MIF dilution with 8 μl of the inhibitors dissolved in DMSO (1 mM). This mixture was pre-incubated for 10 min. Next, 50 μl of the mixture was mixed with 50 μl of 2 mM PP solution in ammonium acetate buffer. Subsequently, MIF tautomerase activity was monitored by the formation of the borate−enol complex, which was measured by the increase in UV absorbance at 300 nm. The increase in UV absorbance was observed over the first 10 min of incubation using a BioTek Synergy H1 Hybrid plate reader. MIF tautomerase activity in the presence of blank DMSO was set to 100% enzyme activity. As the negative control, the enzyme was excluded from monitoring the non-catalyzed conversion of the substrate, which did not show a change in absorbance at 300 nm. Data from the first 3 min were used to calculate the initial velocities. All the graphs were prepared in GraphPad Prism 8.

Jump Dilution Assay

The solution of the enzyme (1.4 µM) was pre-incubated with a saturating concentration of inhibitor MN123 (25 μM) for 10 min. Then the enzyme-inhibitor mixture was diluted 20-fold with boric acid buffer, then 50 μl of the mixture was mixed with 50 μl of 2 mM PP solution in ammonium acetate buffer and recovery of the activity was measured over 20 min. In the control groups, no enzyme/inhibitor was added.

Quantification and Statistical Analysis

All data statistical analysis were preformed using GraphPad Prism 8 for Windows. Description of samples, definition of n, and performed statistic test are indicated in the figure legend. The statistical significance was reported in the relevant Figures. Error bars represent standard error of the mean (SEM) or standard deviation (SD) as indicated.

*
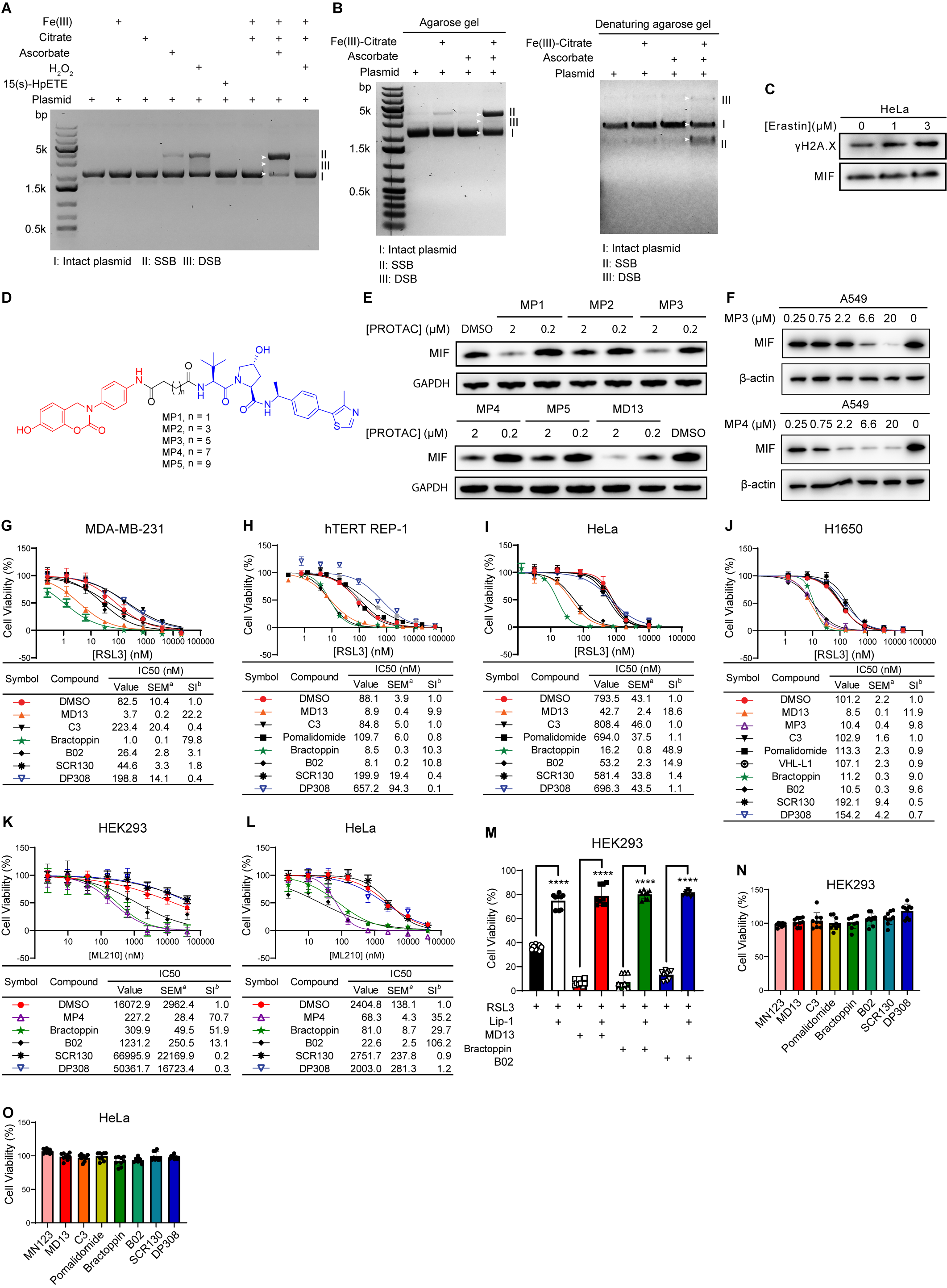
*

*Figure S1. Ferroptosis stimulates double-strand break, which mainly relies on homologous recombination to repair.*

*(A-B) Double-strand DNA damage induced by iron (III) plus reductants in vitro. Samples were separated by a undenaturing or a denaturing agarose gel. The concentration of each component in the reaction mixtures: plasmid 400 ng, Fe (III) 200 µM, Citrate 1mM; sodium ascorbate 200 µM, H_2_O_2_ 200 µM, 15(s)-HpETE 30 µM.*

*(C) Immunoblotting analysis of DSB induced Erastin in HeLa.*

*(D) Chemical structures of MP1-5. The red and blue color indicate the MIF binder and VHL ligand, respectively.*

*(E-F) Immunoblotting analysis of MP1-MP5 and MD13 induced MIF degradation.*

*(G-J) Dose-dependent toxicity of RSL3 in MDA-MB-231 (G), hTERT RPE-1 (H), HeLa (I), and H1650 (J) treated with or without indicated compounds (10 µM). Data are mean ± SEM. n≥3.*

*(K-L) Dose-dependent toxicity of ML210 in Hek293 (K) and HeLa (L) treated with or without indicated compounds (10 µM). Data are mean ± SEM. n≥3.*

*(M) The anti-ferroptosis efficiency of lipr-1 in cells treated with RSL3 only or in combination with indicated compounds. Data are mean ± SEM. ****p<0.0001, ordinary one-way ANOVA followed by Tukey’s test. n≥3.*

*(N-O) Cytotoxicity of indicated compounds (10 µM) on Hek293 and HeLa. Data are mean ± SEM. n≥3.*

*(A-C, E, and F) Uncropped images were shown in Figures S7-S10 as indicated.*

*
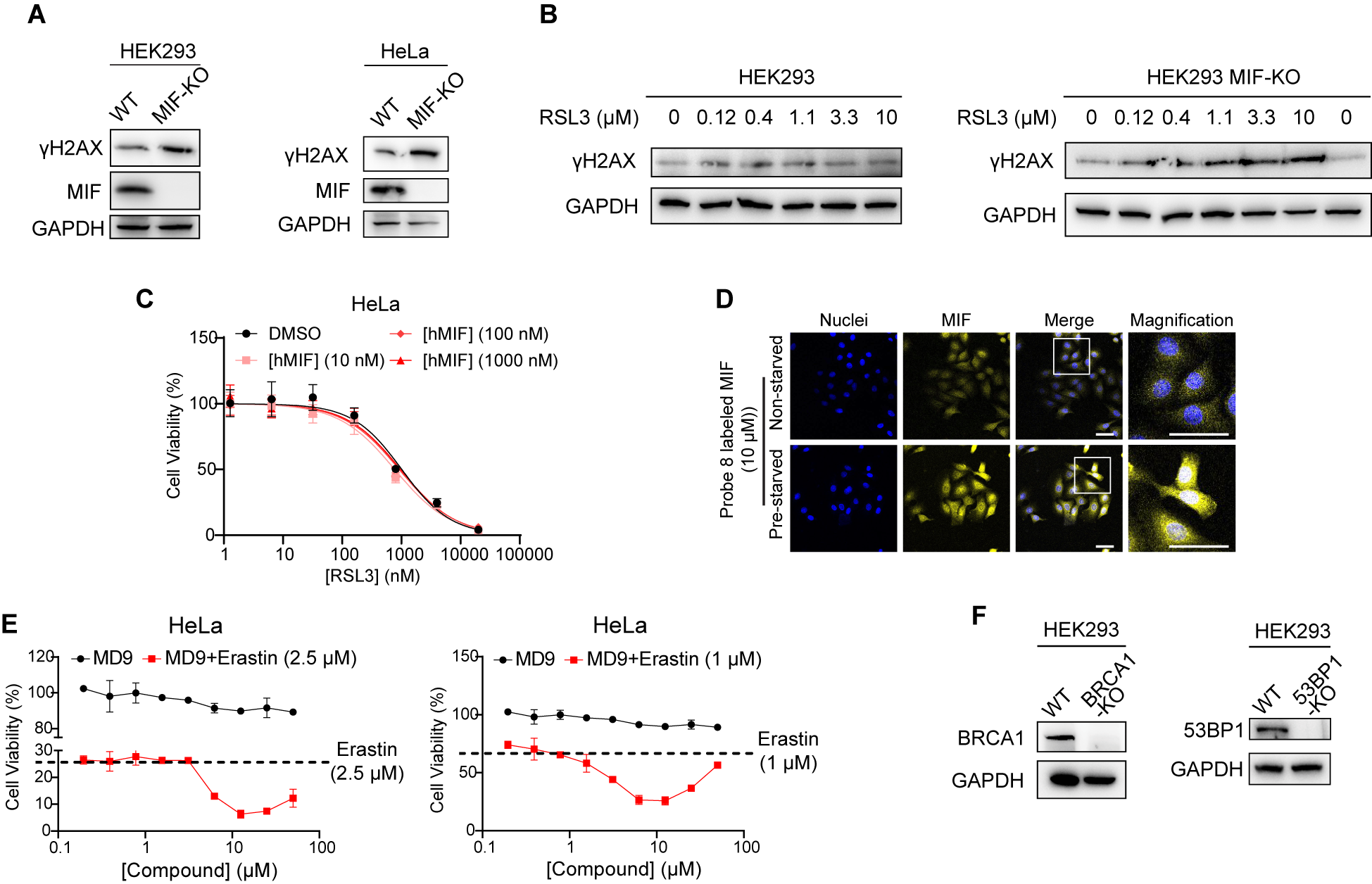
*

*Figure S2. MIF is important for cells against ferroptosis while bacterial produced recombinant MIF has no such function.*

*(A) Immunoblotting analysis of DSB level in MIF-KO Hek293 (left) and HeLa (right).*

*(B) Immunoblotting analysis of DSB level in WT (left) and MIF-KO (right) Hek293 induced by RSL3.*

*(C) Dose-dependent toxicity of RSL3 in HeLa treated with bacterial produced recombinant MIF.*

*(D) Representative images of A549 treated with the fluorescent probe-8-labeled recombinant MIF. Recombinant MIF is uptaken by cells and located in both cytoplasm and nucleus. The white color in the merged pictures indicates the overly of probe-8 labeled MIF and DAPI in the nucleus. Scale bars, 30 µM.*

*(E) Dose-dependent ferroptosis enhancing efficiency of MIF-PROTAC MD9 in HeLa treated with a high dose (left) and a low dose (right) of erastin.*

*(F) Immunoblotting validation of BRCA1-KO (left) and 53BP1-KO (right) in Hek293.*

*(A, B, and F) Uncropped images were shown in Figures S8 and S9 as indicated.*

*
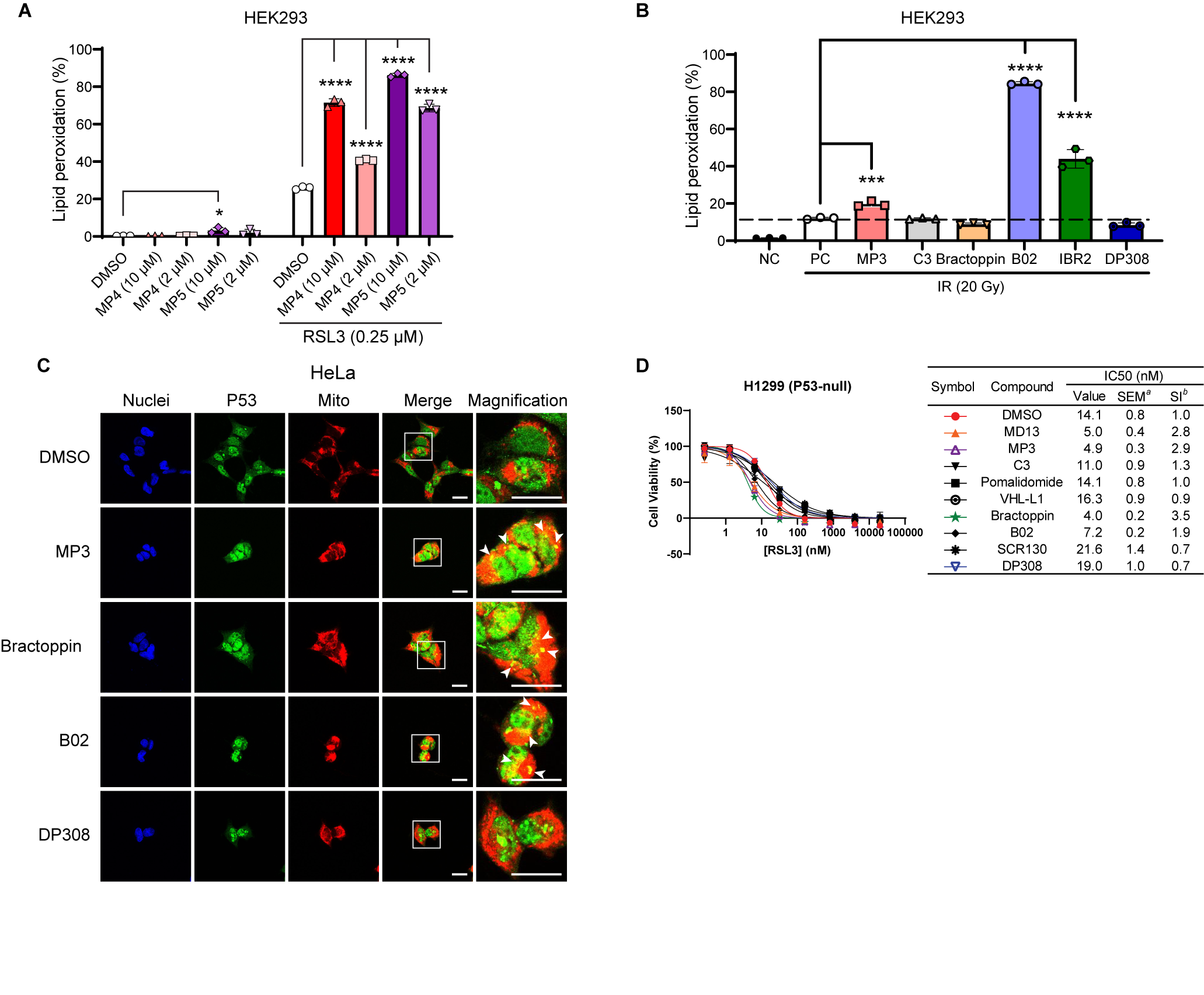
*

*Figure S3. HR inhibition stimulates excessive lipid peroxidation through mitochondrial P53.*

*(A) Lipid peroxidation induced either by indicated PROTACs or PROTACs in combination with RSL3 (0.25 µM, 4h) as indicated. Data are mean ± SEM. *P=0.0373, ****P<0.0001, two-way ANOVA with Dunnett's test, n=3.*

*(B) Lipid peroxidation induced either by IR (20Gy) in combination with compounds (10 µM) as indicated. Data are mean ± SEM. ***p<0.0002, ****p<0.0001, one-way ANOVA with Tukey's test, n=3.*

*(C) Representative images of P53 translocation to mitochondria in HeLa treated with indicated compounds (10 µM, overnight). Scale bars, 30 µM. The yellow color in the merged pictures indicates the colocalization of mitochondria and P53 in cytoplasm. Images are from one representative of three independent experiments.*

*(D) Dose-dependent toxicity of RSL3 in H1299 treated with or without indicated compounds (10 µM). Data are mean ± SEM, n=3; ^a^SEM: standard error of the mean; ^b^SI: sensitizing index.*

*
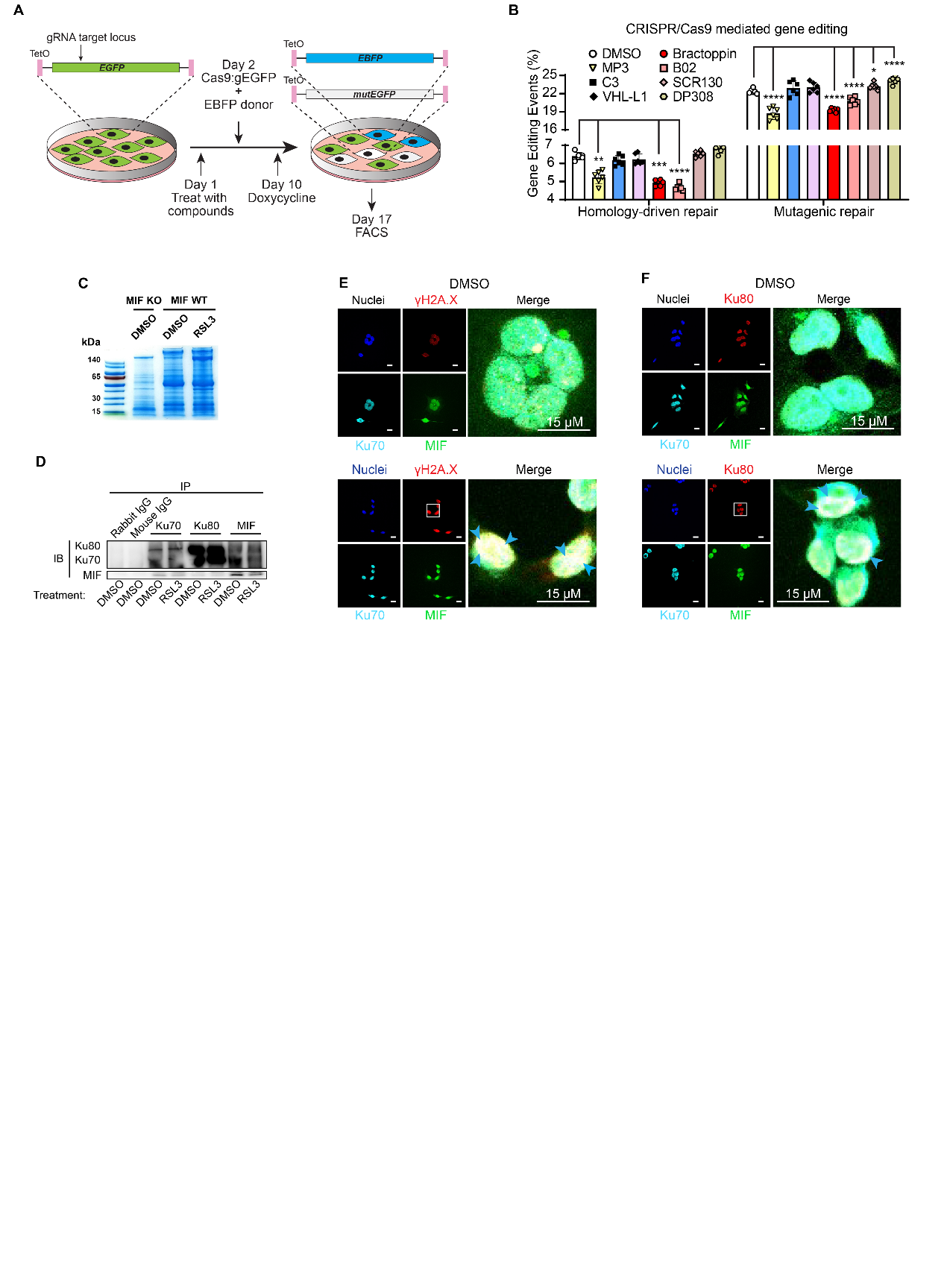
*

*Figure S4. MIF cooperates with Ku70 and Ku80 facilitating HR in ferroptosis.*

*(A) Experimental scheme of the GFP-to-BFP conversion assay in HEK.EGFP^TetO.KRAB^ for determination of compounds’ effect on DNA repairing pathway choice.*

*(B) Gene editing frequency determined by flow cytometry in HEK.EGFP^TetO.KRAB^ treated with indicated compounds (10µM) and transfected with an EGFP-sgRNA, Cas9-WT, and ssDNA template. Data are mean ± SEM. *p<0.0332, **p<0.0021, ***p<0.0002, ****p<0.0001, two-way ANOVA, n=6.*

*(C) Immunoprecipitation (IP) of endogenous MIF in parental and MIF-KO HeLa cells treated with or without RSL3 (4 µM, 2h) followed by electrophoresis.*

*(D) IP of endogenous MIF, Ku70, and Ku80 in Hek293 treated with or without RSL3 (4 µM, 2h)* *followed by immunoblotting detection of MIF, Ku70, and Ku80.*

*(E) Representative confocal images of colocalization of MIF, Ku70, and γH2A.X in Hek293.* *MIF was detected by a MIF specific fluorescent probe, ZP307. The white color (arrowed) in the merged pictures indicates the colocalization of MIF, Ku70, and γH2A.X.*

*(F) Representative confocal images of colocalization of MIF, Ku70, and Ku80 in Hek293. MIF was detected by ZP307. The white color (arrowed) in the merged pictures indicates the colocalization of MIF, Ku70, and Ku80.*

*(D) Uncropped images were shown in Figures S10 as indicated.*

*
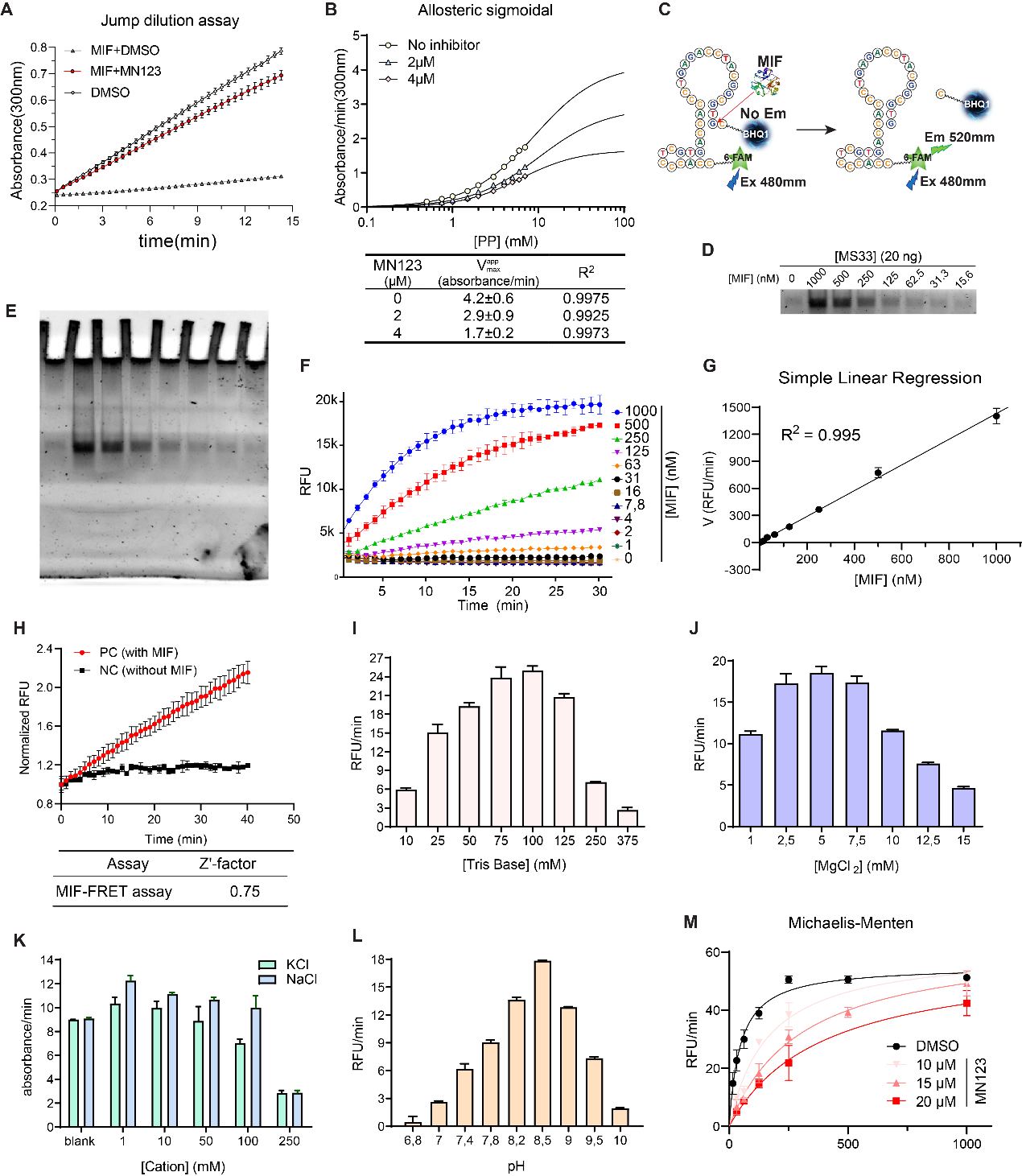
*

*Figure S5a. Optimization of the MIF-FRET assay for nuclease activity.*

*(A) Jump dilution assay of MIF tautomerase in the presence of MN123. Data are mean, n=3.*

*(B) Allosteric sigmoidal plots of MIF tautomerase activity in the presence of 0, 2, and 4 µM of MN123.*

*(C) Schematic representation of the MIF-FRET nuclease assay.*

*(D)* *MIF-mediated dose-dependent cleavage of MS33 detected by polyacrylamide gel electrophoresis.*

*(E) Uncropped image of Figure S5D.*

*(F) MIF-mediated dose-dependent cleavage of MS33 detected by a fluorescence plate reader.*

*(G) Simple linear regression quantification of MIF-mediated does-dependent cleavage of MS33 detected by a fluorescence plate reader.*

*(H) Z’-factor value for the MIF-FRET assay. Data are mean ± SEM. Assays were performed in 15 technical replicates.*

*(I-L) Optimization of the reaction buffer of the MIF-FRET assay with different concentration of tris base (I), MaCl_2_ (J), and Cations (K), as well as different pH (L).*

*(M) Michaelis-Menten plots of MIF nuclease in the presence of 0, 10, 15, and 20 µM of MN132. Data are mean, n=2.*

*
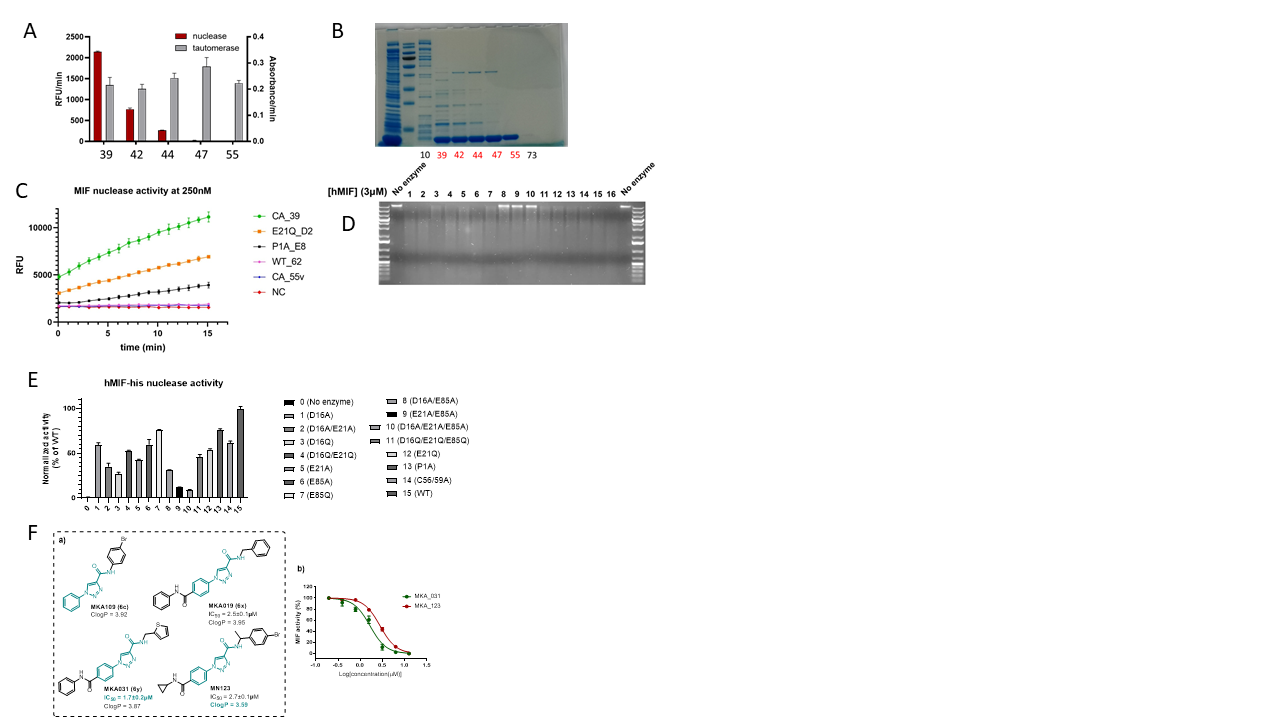
*

*Figure S5b. Optimization of the MIF-FRET assay and characterization of MN123.*

*(A and B) Nuclease and tautomerase activity assay on different MIF fractions. The concentration is normalized by BCA. The nuclease activity is decreasing while tautomerase activity stays the same. Unable to prove if the decrease in nuclease activity is due to actual activity or the absence of impurities.*

*(C) Fret assay. E21Q is supposed to abolish nuclease activity but it is not reproducible. The enzyme activity is fraction-dependent (the concentration is normalized by BCA protein assay)*

*(D and E) Mutations of the putative active site. The activity of mutants is not consistent (for example E21Q is the same as P1A, C56/59A and WT) C56A&C59A: Cysteine (C) replaced with alanine (A). Blocks oxidoreductase activity P1A: Proline (P) replaced with alanine (A). Blocks tautomerase activity*

*
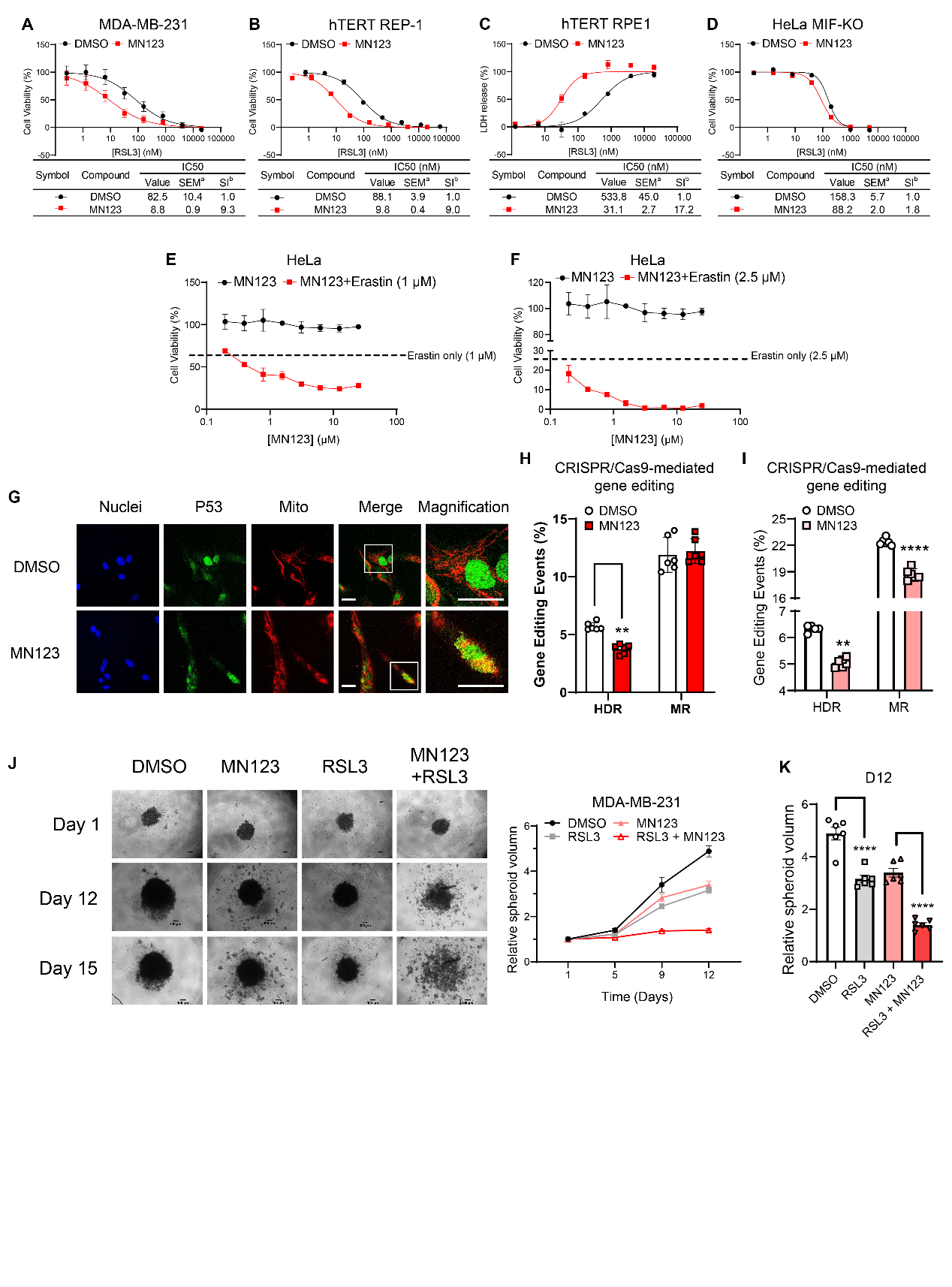
*

*Figure S6. MN123 enhances ferroptosis in various types of cells.*

*(A and B) Dose-dependent toxicity of RSL3 in MDA-MB-231 (A) and hTERT REP-1 (B) treated with or without MN123 (10 µM). Data are mean ± SEM. n≥3.*

*(C) LDH released by hTERT RPE-1 cells for analysis of dose-dependent cytotoxicity of RSL3 combined with or without MN123 (10 µM). Data are mean ± SEM. n≥3.*

*(D) Dose-dependent toxicity of RSL3 in HeLa MIF-KO (D) treated with or without MN123 (10 µM). Data are mean ± SEM. n≥3.*

*(E and F) Dose-dependent ferroptosis enhancing efficiency of MN123 in HeLa treated with a low dose (E) and a high dose (F) of erastin.*

*(G) Representative images of P53 translocation to mitochondria in hTERT REP-1 WT cells treated with MN123 (10 µM, overnight). Scale bars, 30 µM. The yellow color in the merged pictures indicates the colocalization of mitochondria and P53 in cytoplasm. Images are from one representative of three independent experiments.*

*(H and I) Gene editing frequency determined by flow cytometry in HEK.EGFP^TetO.KRAB^*

*(H) and H27 (I) treated with MN123 (10µM) and transfected with an EGFP-sgRNA, Cas9-WT, and ssDNA template. Data are mean ± SEM. **p<0.0021, two-way ANOVA, n=6.*

*(J) Representative images and quantifications of 3D spheroid formation assay in MDA-MB-231 treated with RSL3 (50 nM) with or without MN123 (10 µM). Data are mean ± SME, n=6.*

*(K) Quantification of (I) at day 12. Data are mean ± SME, one-way ANOVA with Tukey’s test, n=6.*

*
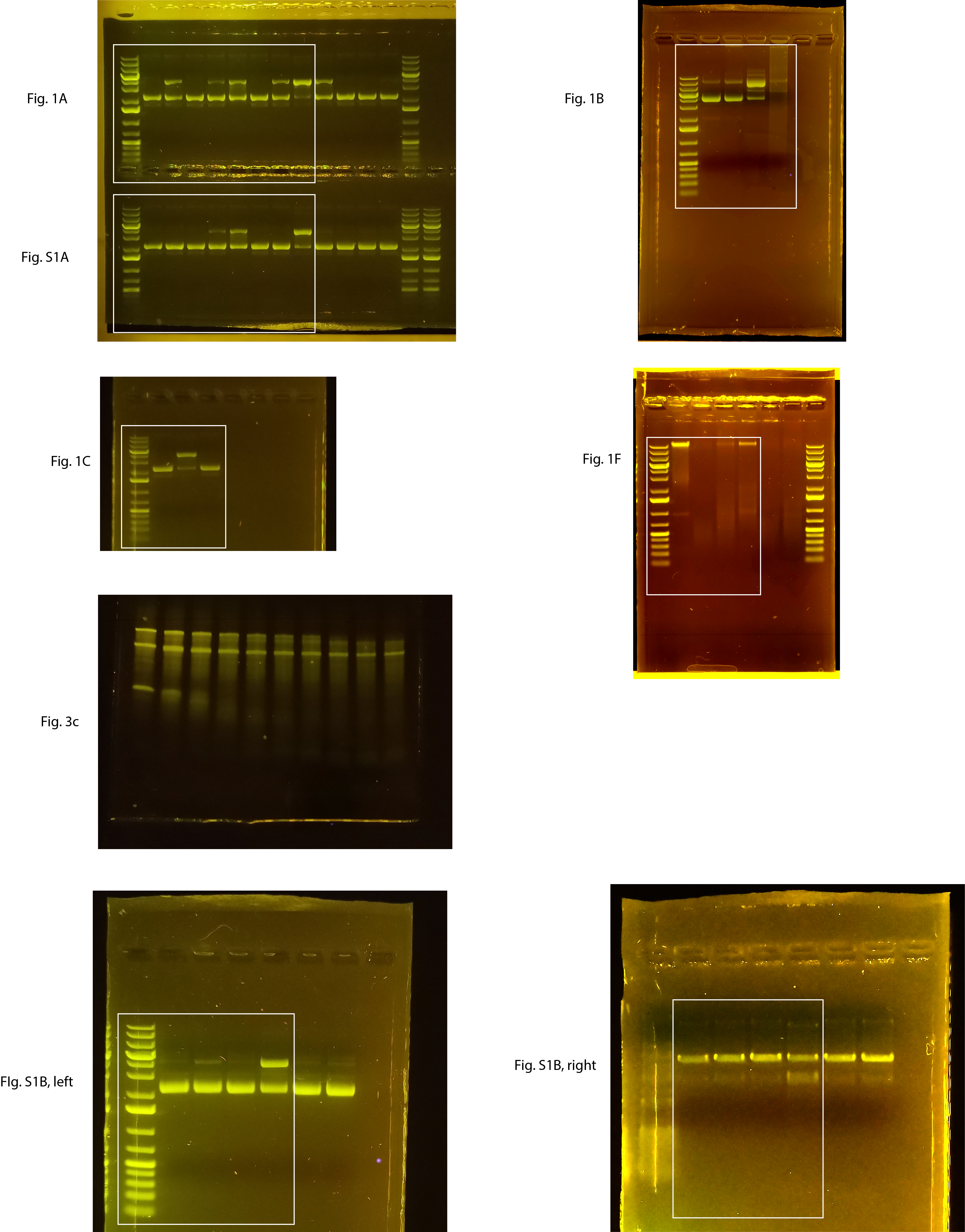
*

*Figure S7. Uncropped gels for the indicated Figures.*

*
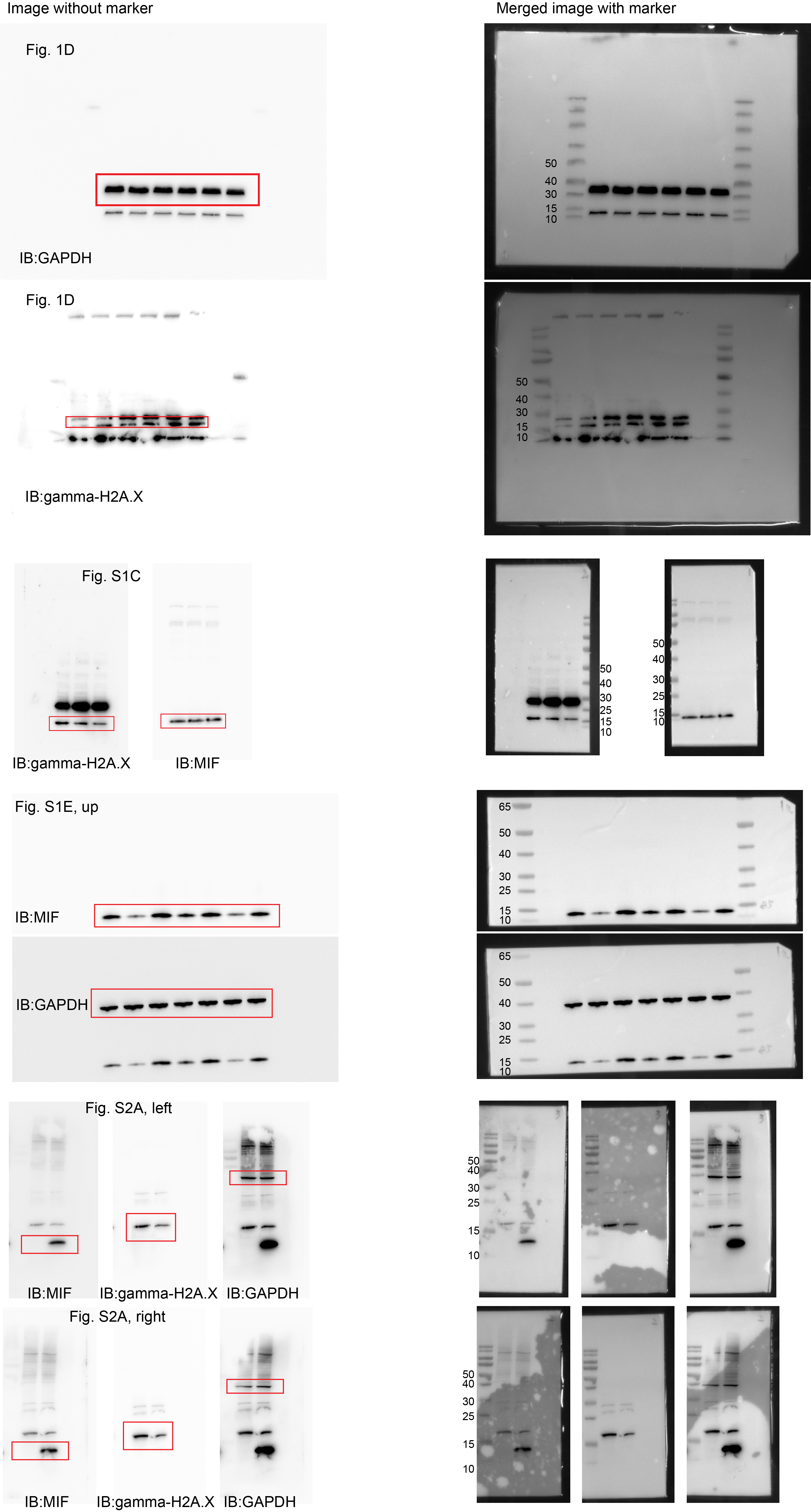
*

*Figure S8. Uncropped blots for the indicated Figures.*


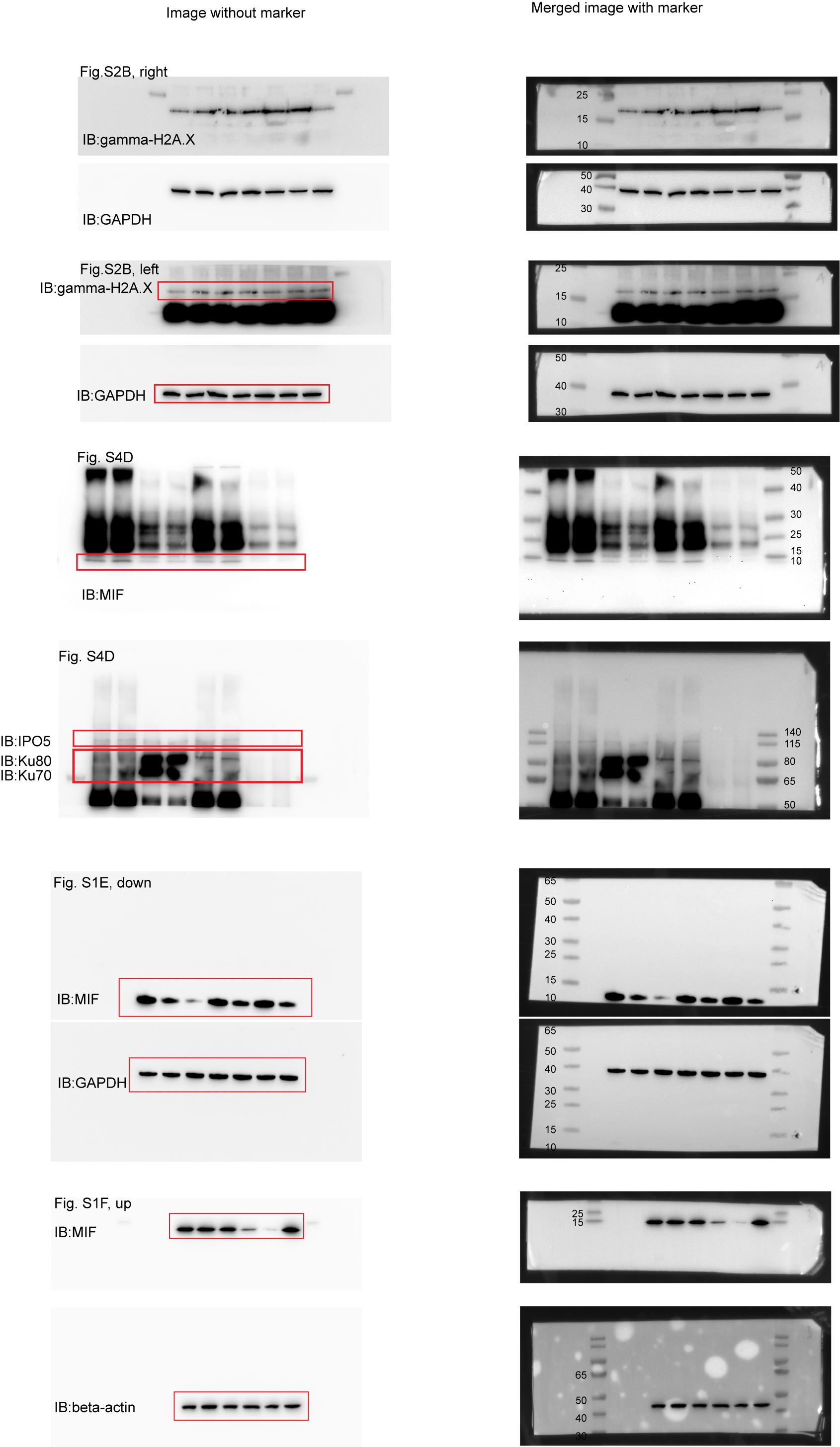


*Figure S9. Uncropped blots for the indicated Figures.*

*
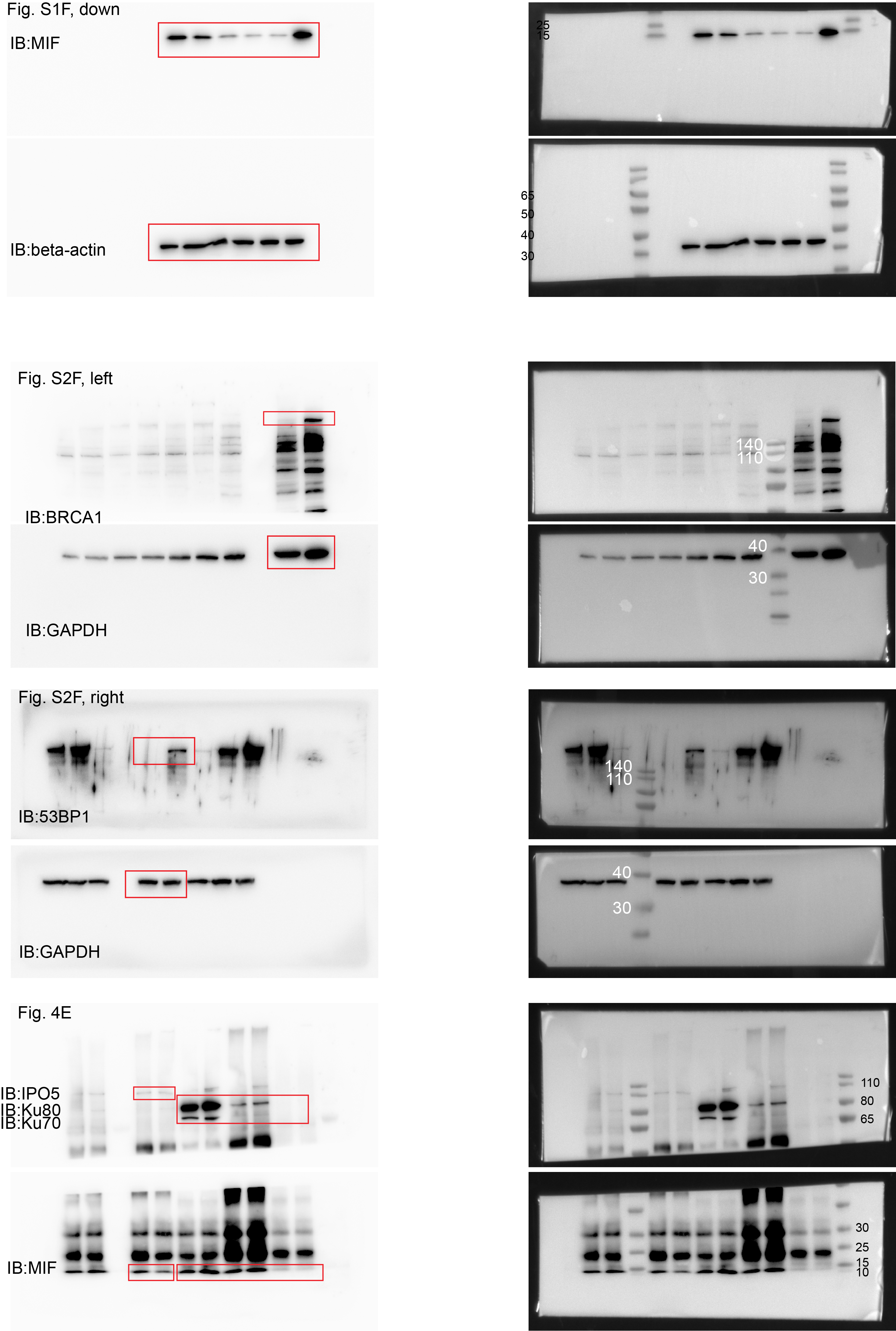
*

*Figure S10. Uncropped blots for the indicated Figures.*

**The NMR spectra of target compounds**

^1^H NMR of **MP1** in DMSO-*d_6_*


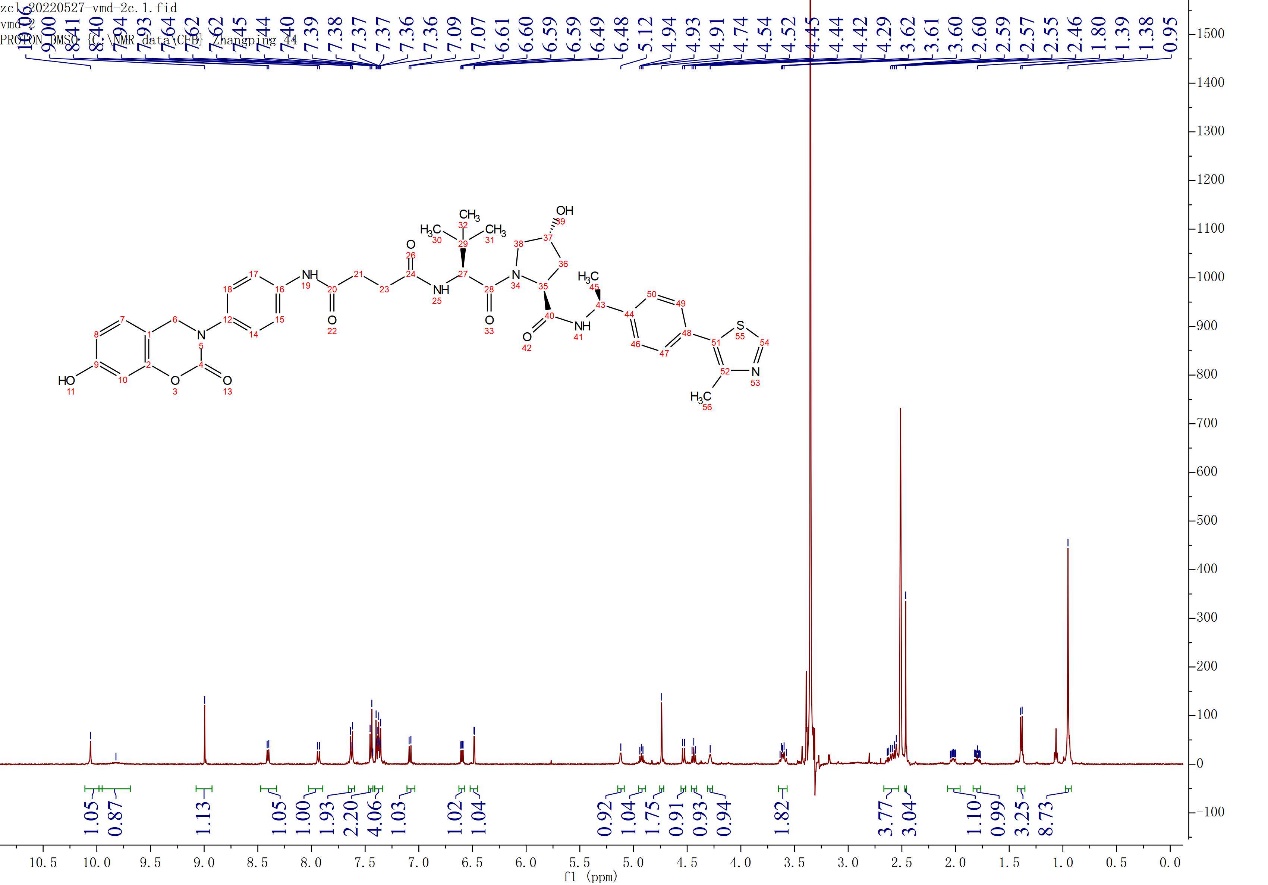


^13^C NMR of **MP1** in DMSO-*d_6_*


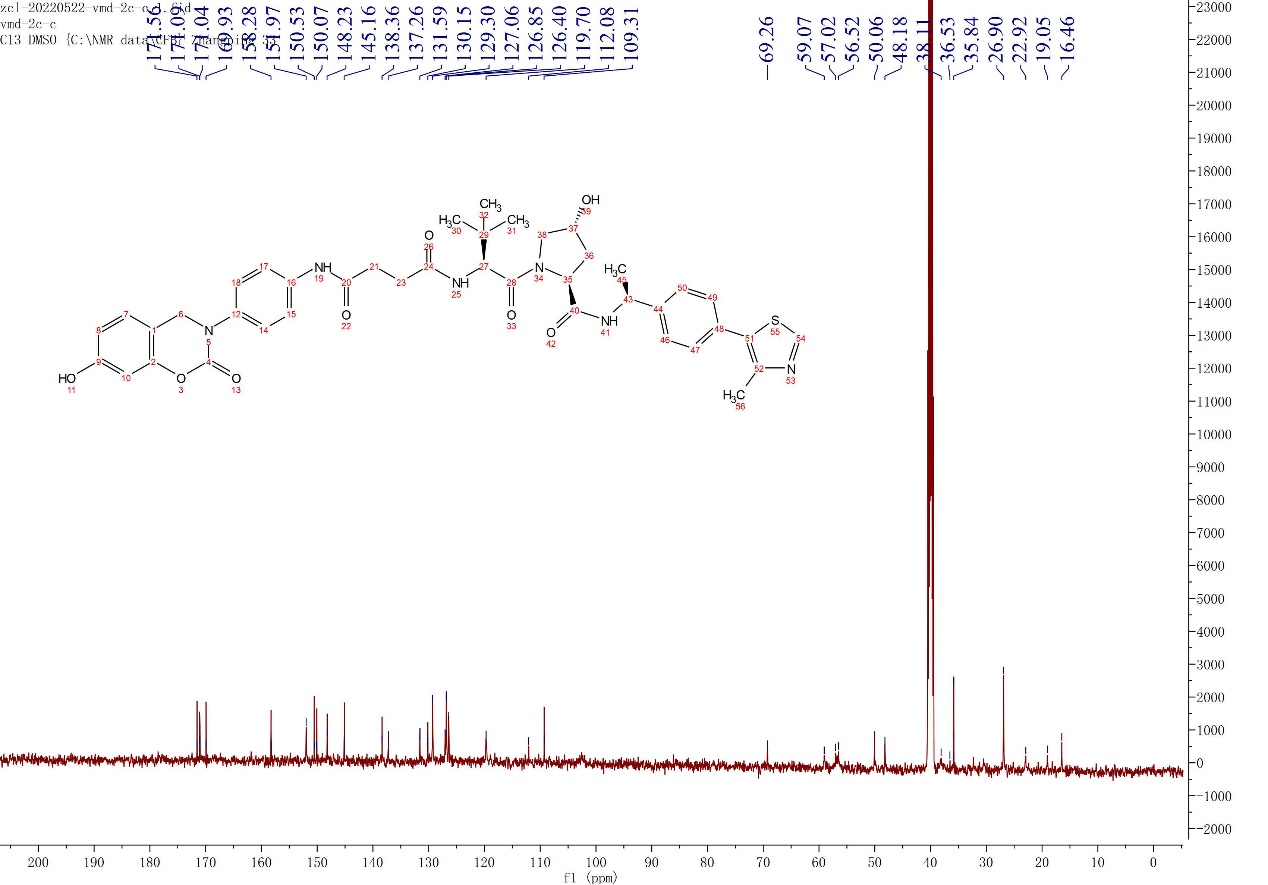


^1^H NMR of **MP2** in DMSO-*d_6_*


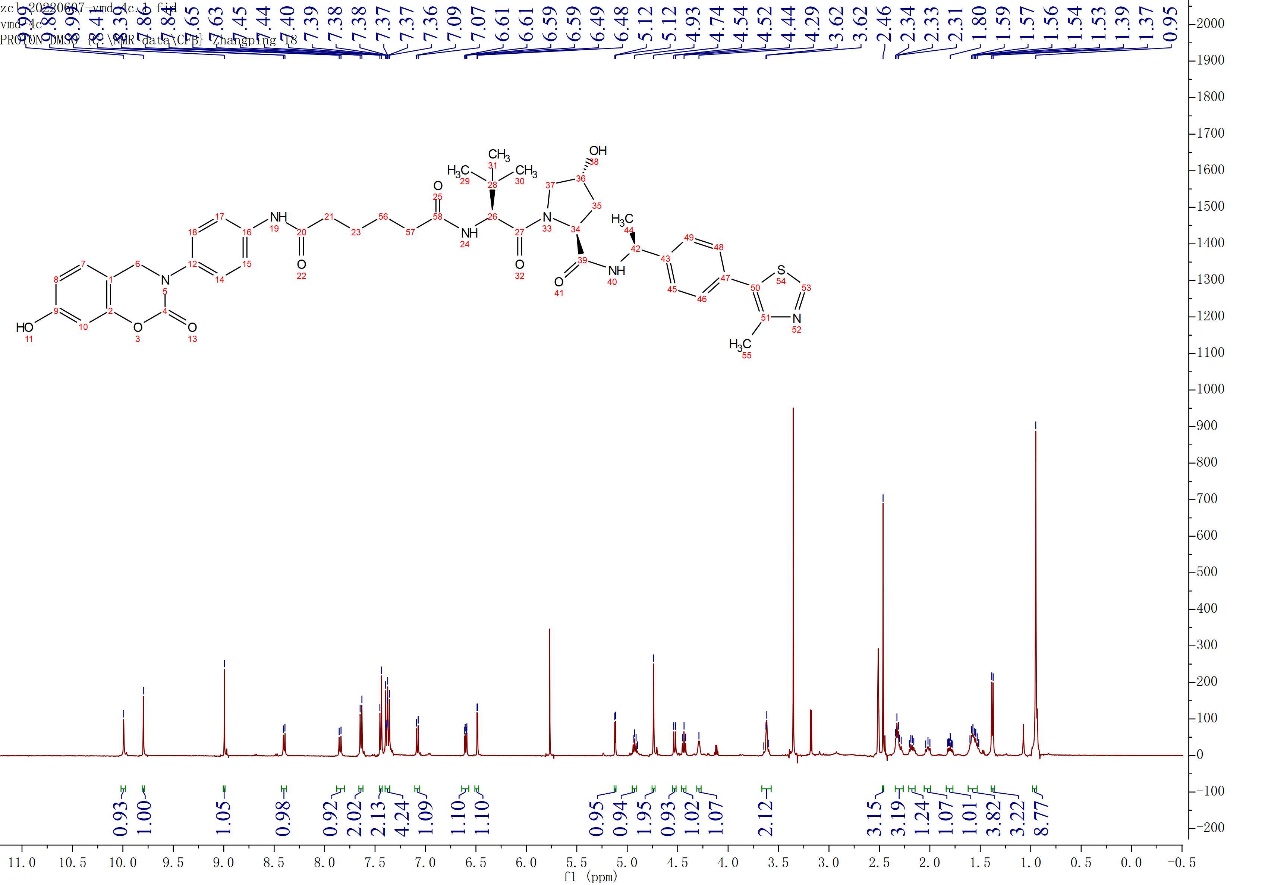


^13^C NMR of **MP2** in DMSO-*d_6_*


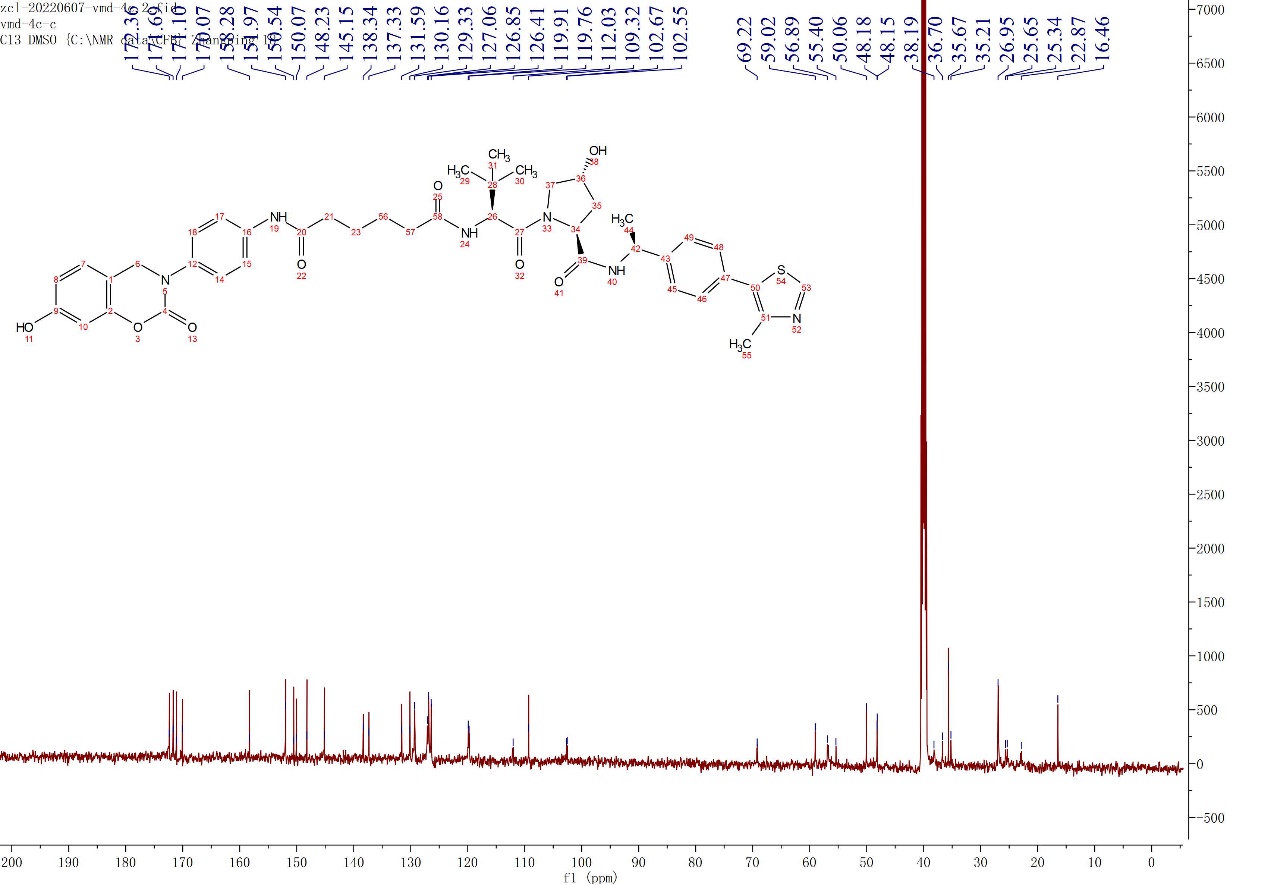


^1^H NMR of **MP3** in DMSO-*d_6_*


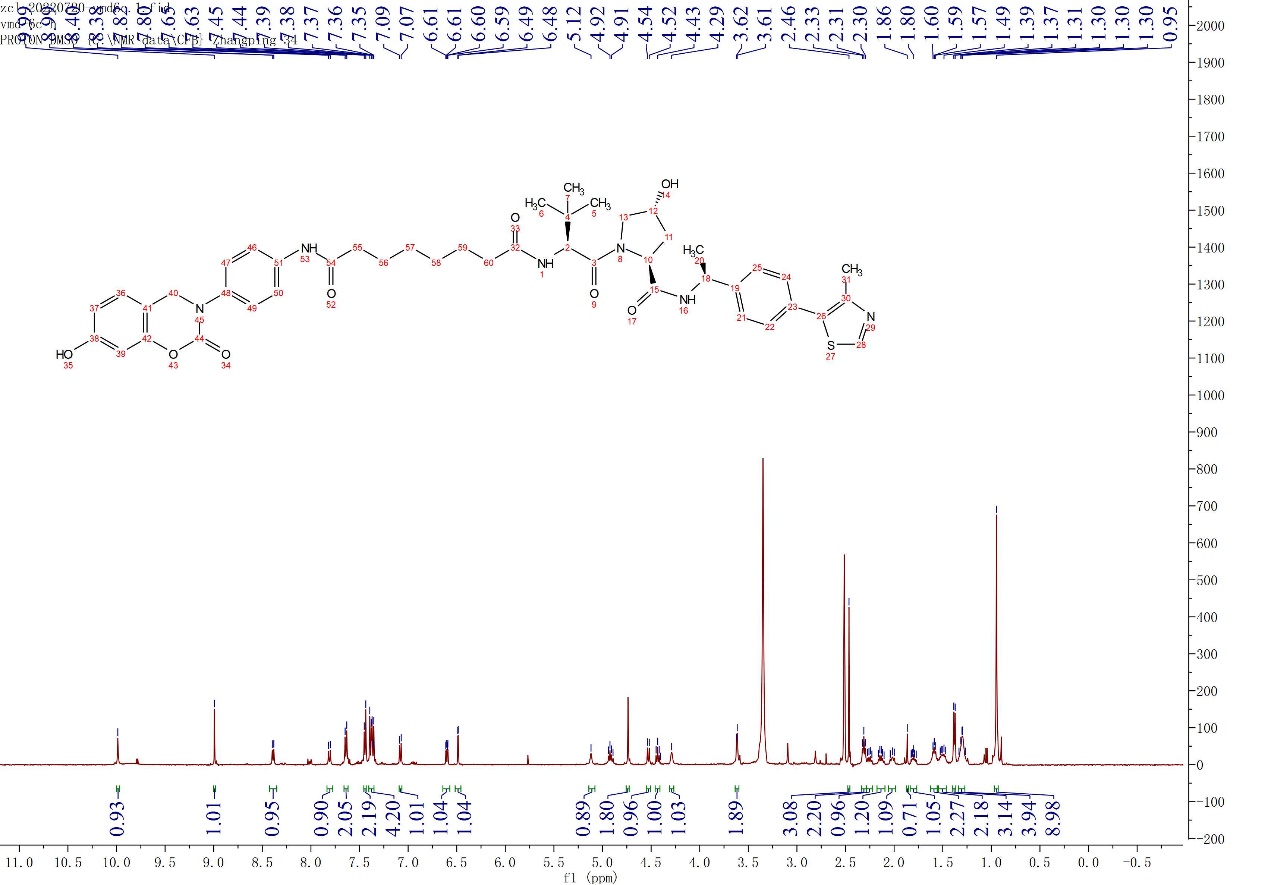


^13^C NMR of **MP3** in DMSO-*d_6_*


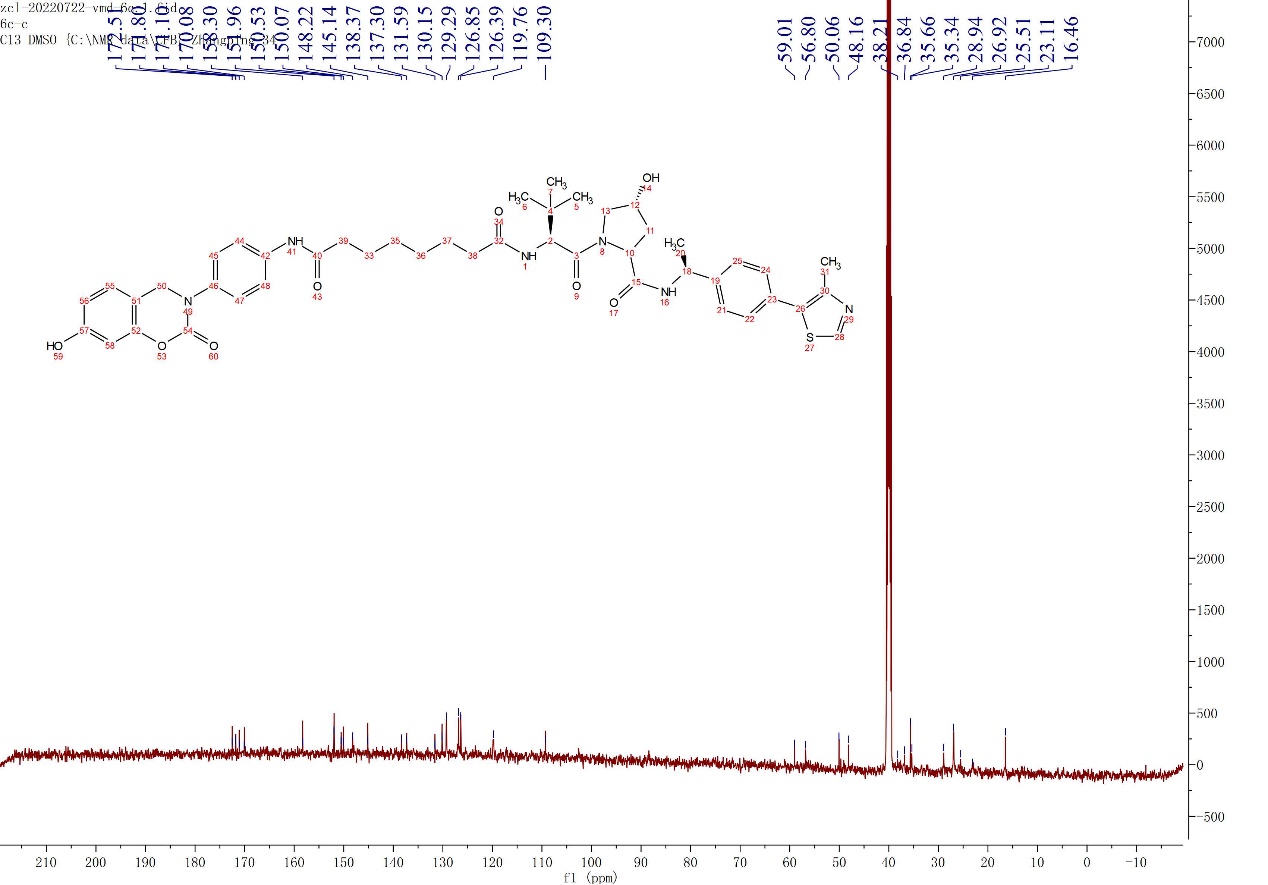


^1^H NMR of **MP4** in DMSO-*d_6_*


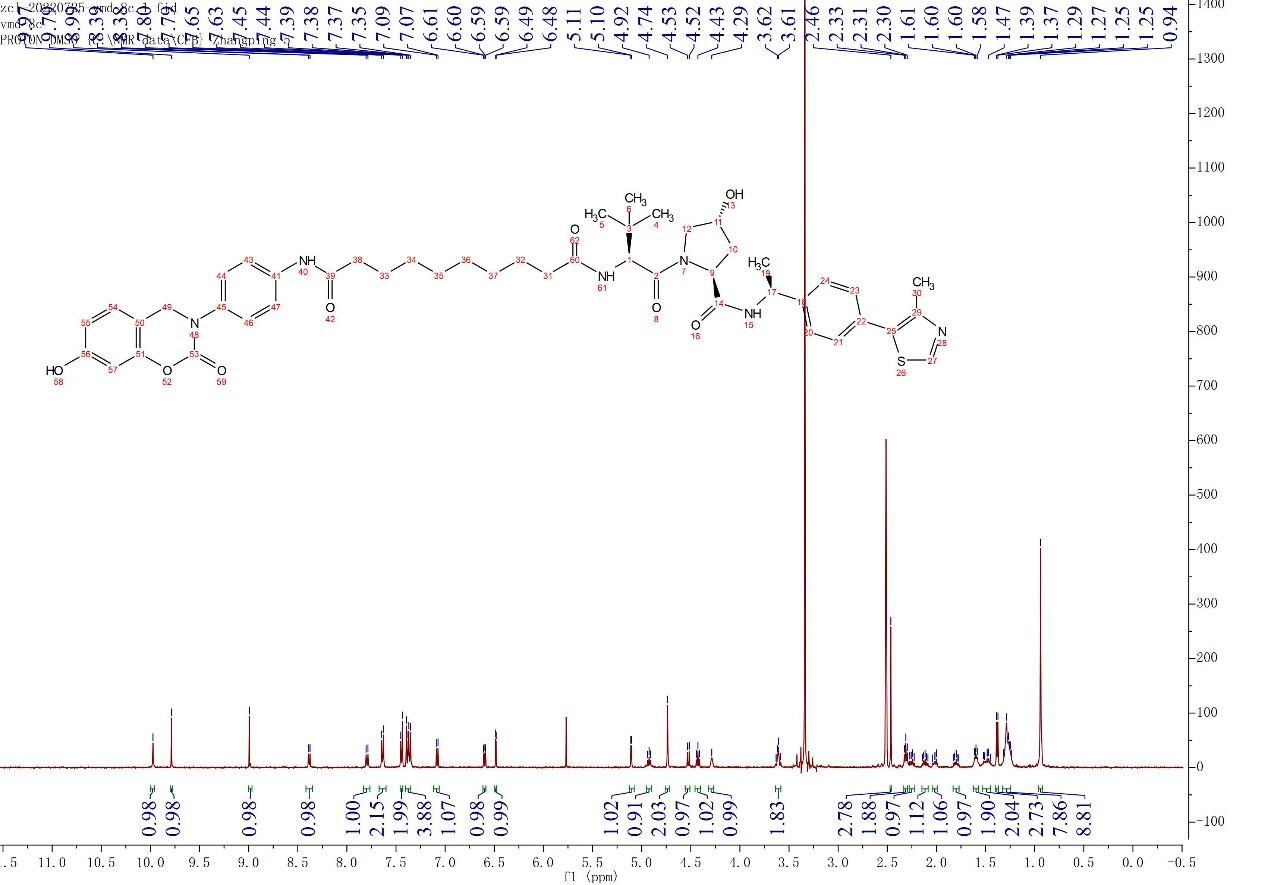


^1^H NMR of **MP5** in DMSO-*d_6_*


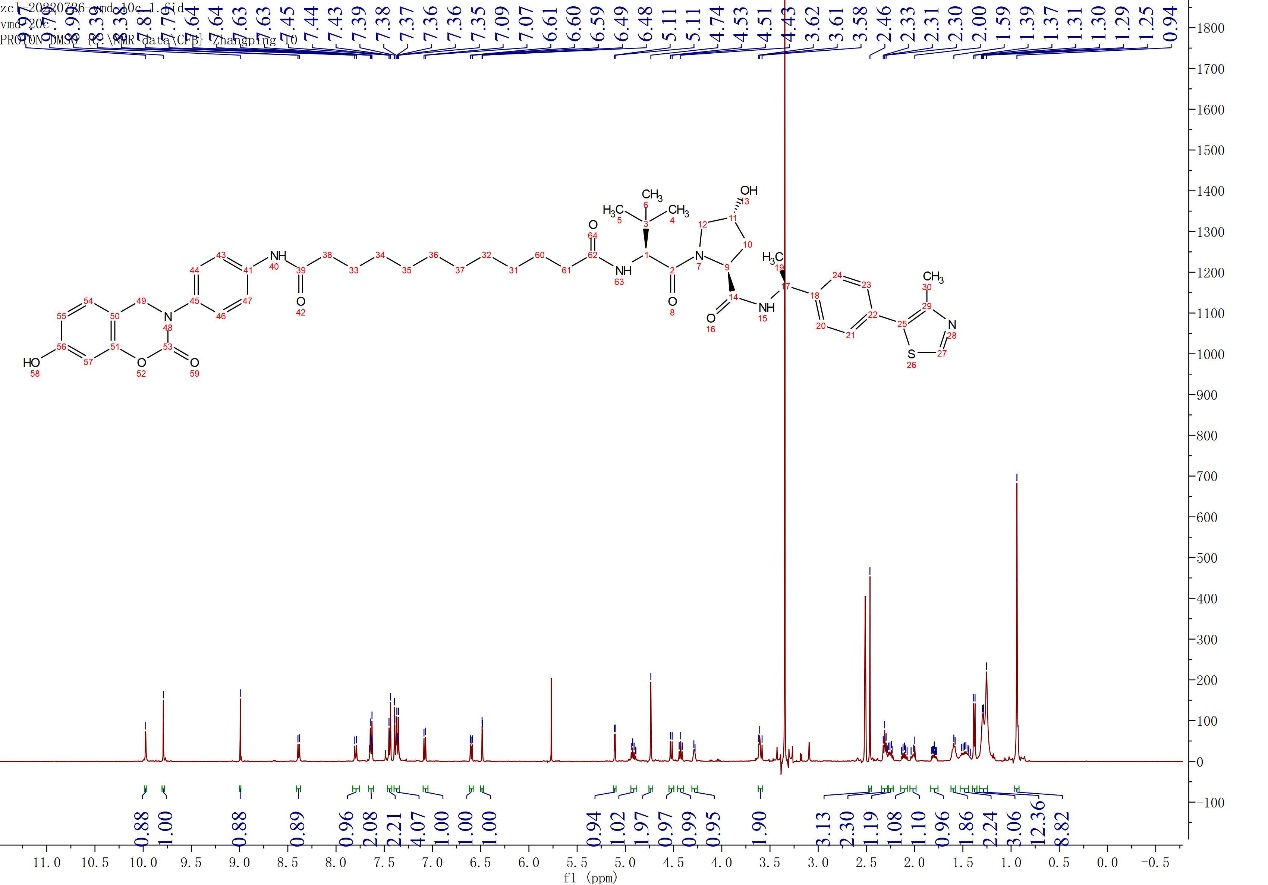


^13^C NMR of **MP5** in DMSO-*d_6_*


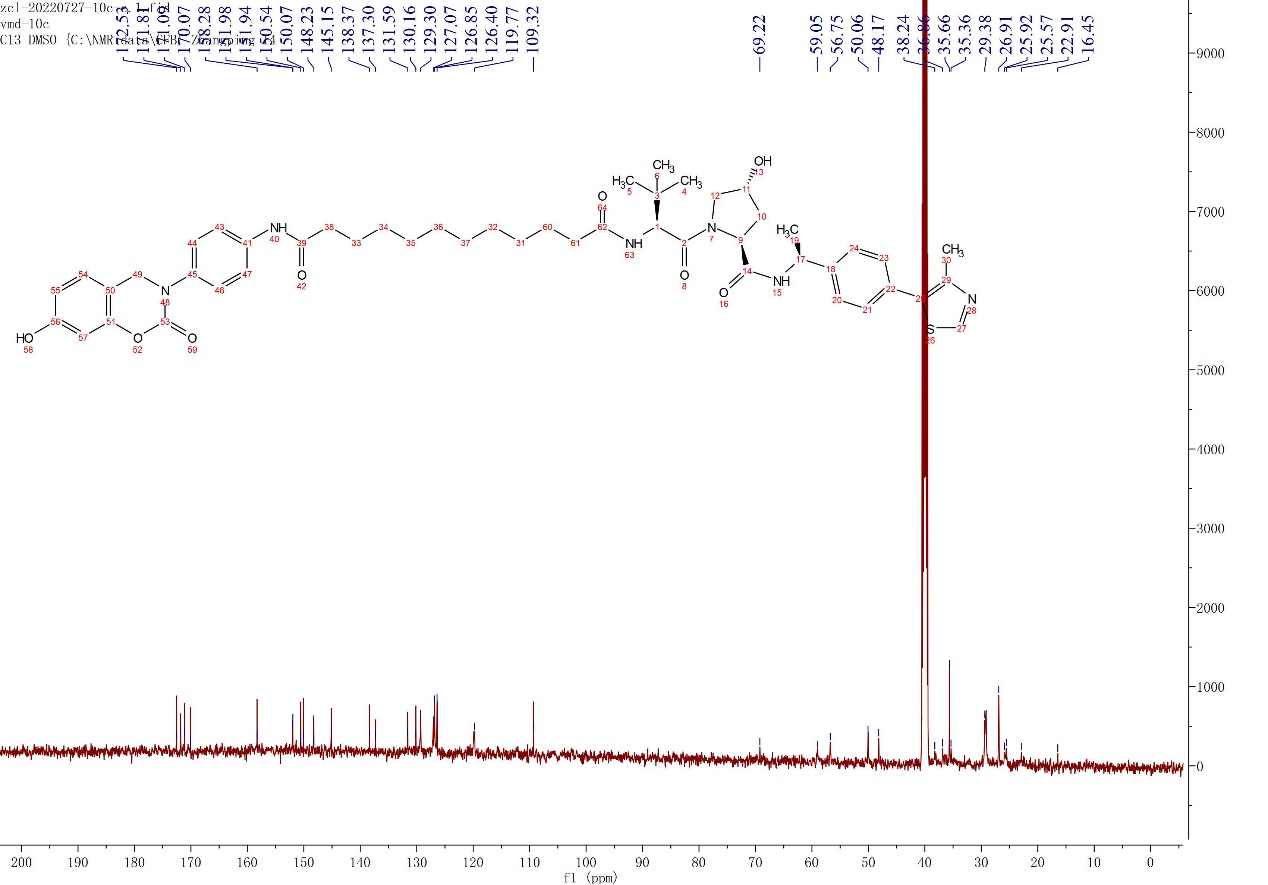


^1^H NMR of **DP308** in CDCL_3_


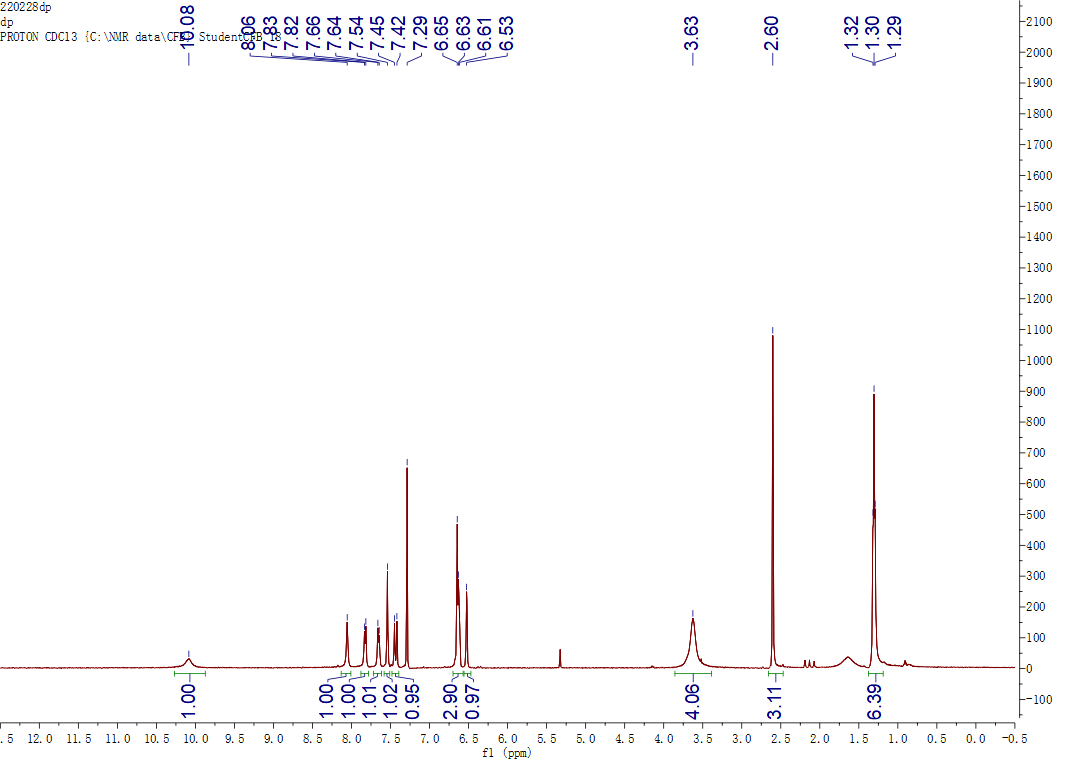


^1^H NMR of **MN123** in DMSO-*d_6_*

^13^C NMR of **MN123** in DMSO-*d_6_*

**The HPLC spectra of target compounds**

**MP1:** retention time: 4.29 min, purity: 95.3%.

**
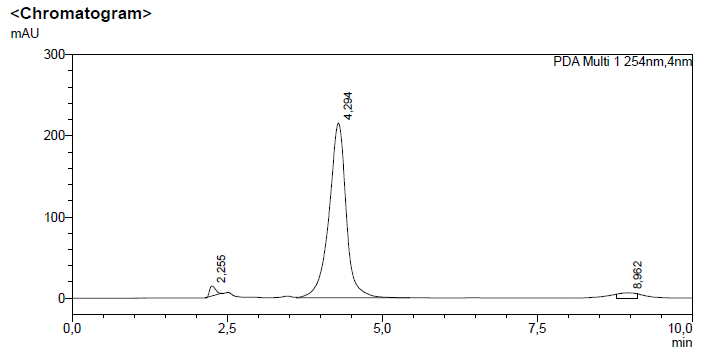
**

**MP2:** retention time: 4.77 min, purity: 95.3%.


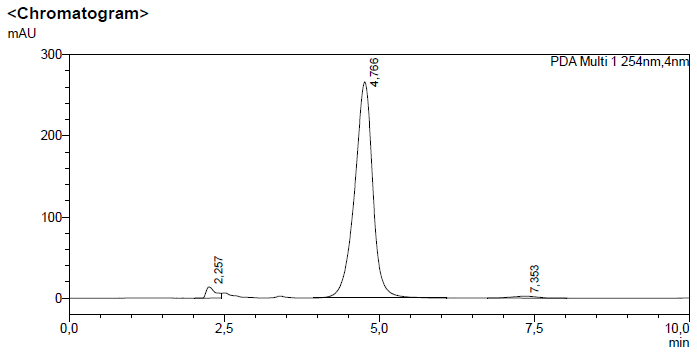


**MP3:** retention time: 4.21 min, purity: 95.2%.

**
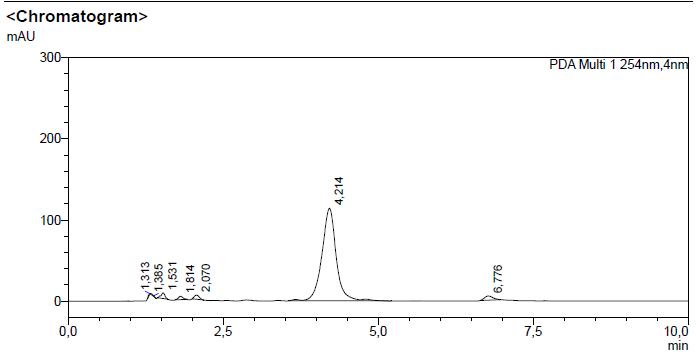
**

**MP4:** retention time: 7.35 min, purity: 97.7%.


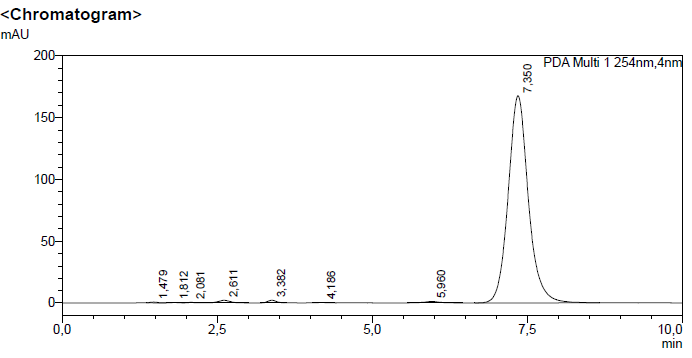


**MP5:** retention time: 3.89 min, purity: 95.0%.

**
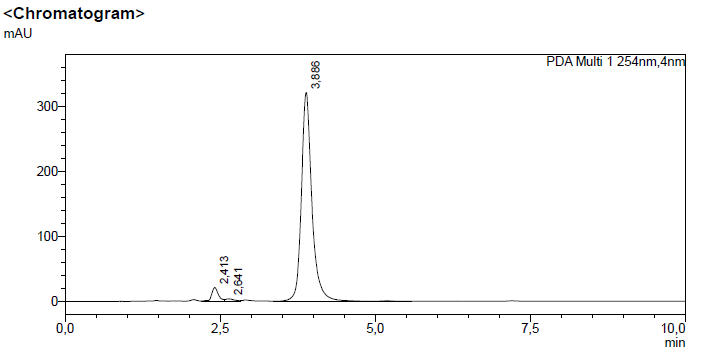
**

**The HRMS spectra of target compounds**

**MP1**


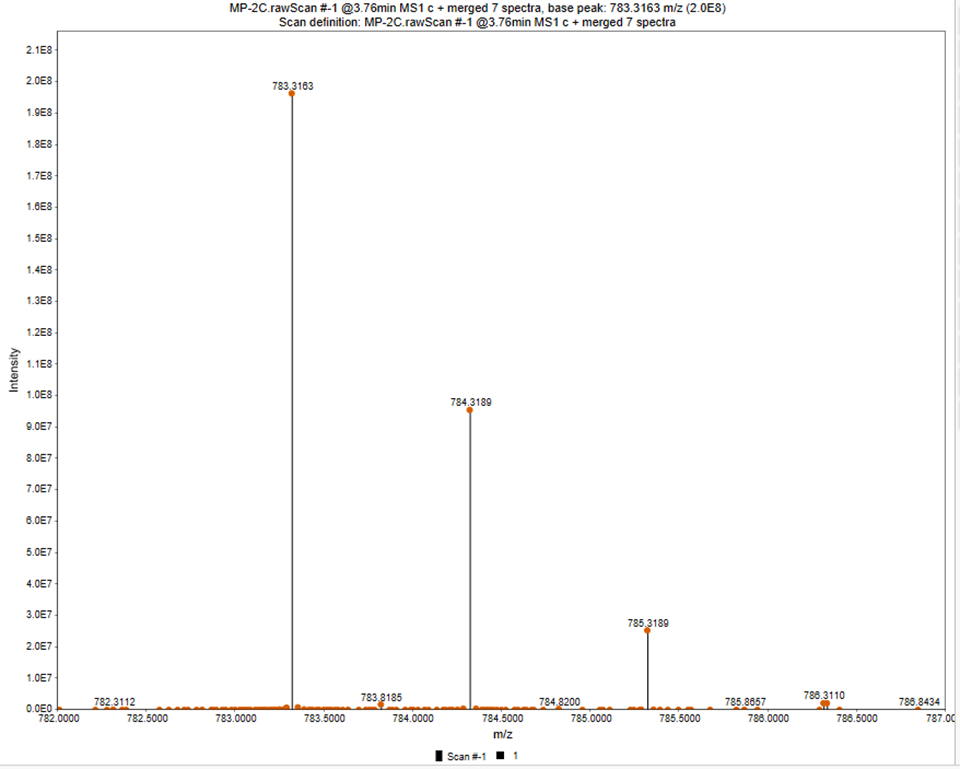


**MP2**


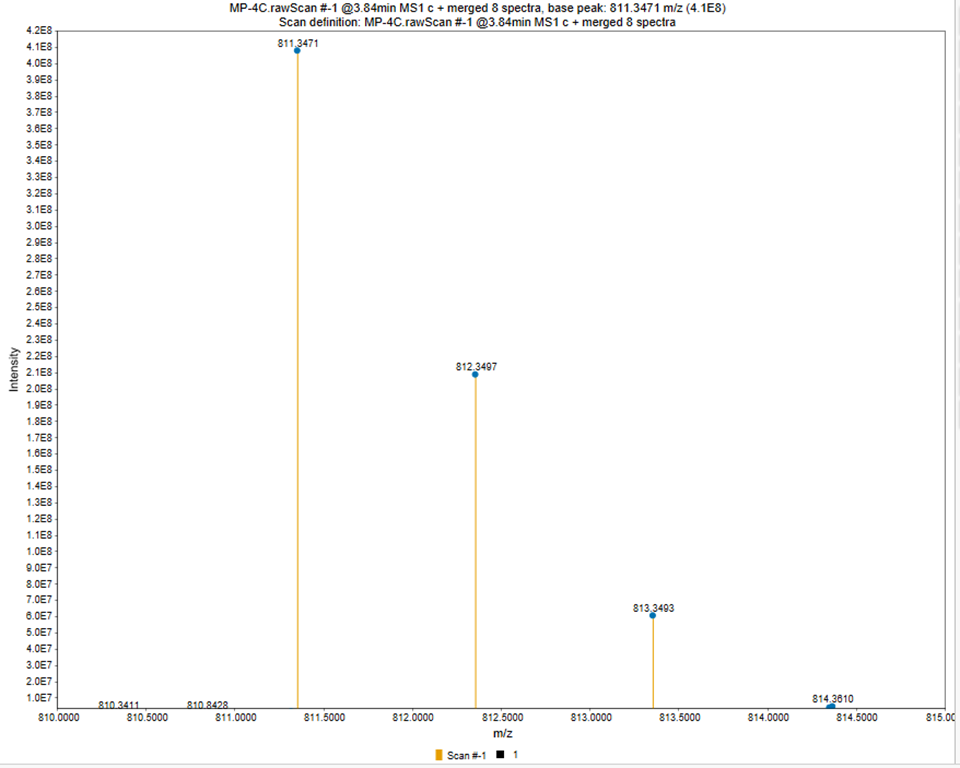


MP3


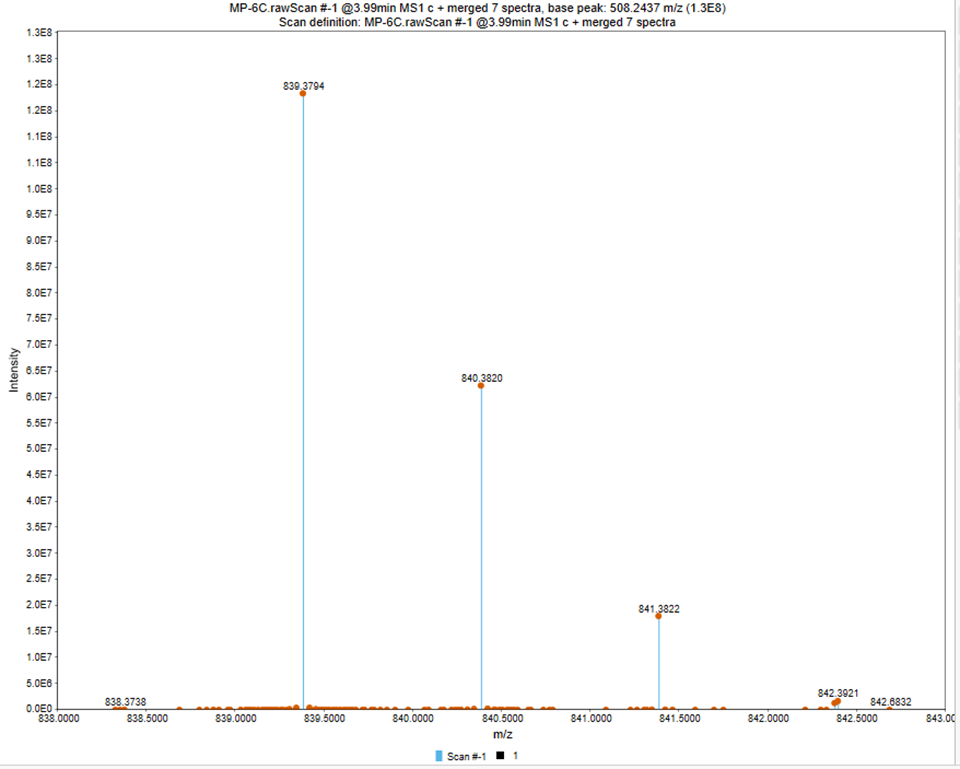


**MP4**


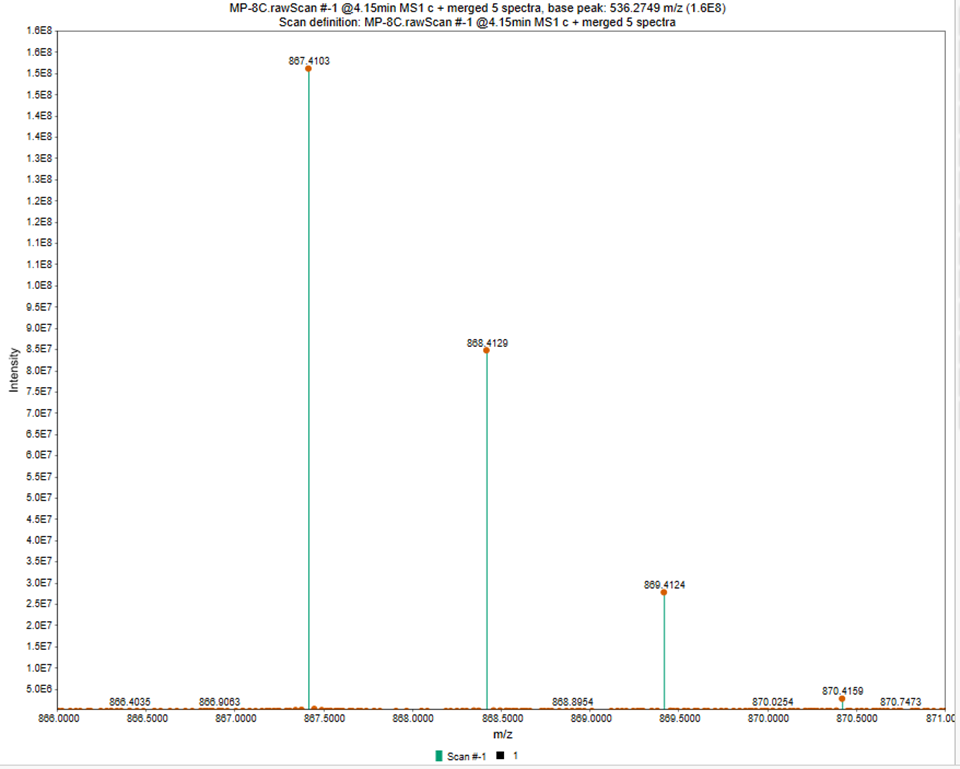


**MP5**


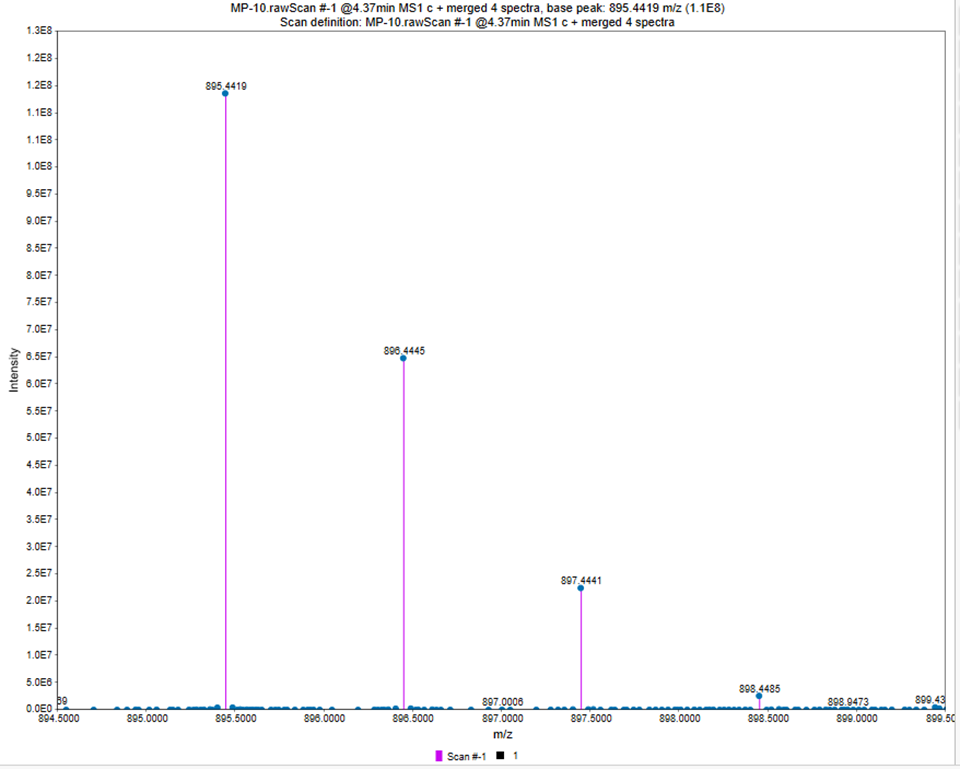

Supplement: Supplementary file 1 — Supporting Information [file ADVS-11-2403963-s001.docx]
